# Efficient differentiation of human iPSCs into Leydig-like cells capable of long-term stable secretion of testosterone

**DOI:** 10.1016/j.stemcr.2024.102392

**Published:** 2025-01-16

**Authors:** Katsuya Sato, Michiyo Koyanagi-Aoi, Keiichiro Uehara, Yosuke Yamashita, Masakazu Shinohara, Suji Lee, Anika Reinhardt, Knut Woltjen, Koji Chiba, Hideaki Miyake, Masato Fujisawa, Takashi Aoi

**Affiliations:** 1 Division of Stem Cell Medicine, Graduate School of Medicine, Kobe University, Kobe, Japan; 2 Division of Advanced Medical Science, Graduate School of Science, Technology and Innovation, Kobe University, Kobe, Japan; 3 Division of Urology, Graduate School of Medicine, Kobe University, Kobe, Japan; 4 Center for Human Resource Development for Regenerative Medicine, Kobe University Hospital, Kobe, Japan; 5 Department of Diagnostic Pathology, Graduate School of Medicine, Kobe University, Kobe, Japan; 6 The Integrated Center for Mass Spectrometry, Graduate School of Medicine, Kobe University, Kobe, Japan; 7 Division of Molecular Epidemiology, Graduate School of Medicine, Kobe University, Kobe, Japan; 8 Department of Life Science Frontiers, Center for iPS Cell Research and Application (CiRA), Kyoto University, Kyoto, Japan; 9 Division of Signal Pathways, Biosignal Research Center, Kobe University, Kobe, Japan

**Keywords:** iPS cell, Leydig cell, testosterone, differentiation, LOH syndrome

## Abstract

Late-onset hypogonadism (LOH) syndrome is characterized by age-related testosterone deficiency and negatively affects the quality of life of older men. A promising therapeutic approach for LOH syndrome is transplantation of testosterone-producing Leydig-like cells (LLCs) derived from human induced pluripotent stem cells (hiPSCs). However, previous studies have encountered obstacles, such as limited cell longevity, insufficient testosterone production, and inefficiency of differentiation. To address these issues, we developed a novel protocol that includes forced *NR5A1* expression, a cytokine cocktail promoting mesoderm differentiation, and a transitional shift from 3D to 2D cultures. The resultant cells survived on culture dishes for over 16 weeks, produced 22-fold more testosterone than the conventional method, and constituted a homogeneous population of LLCs with a differentiation efficiency exceeding 99% without purification. Furthermore, these LLCs were successfully engrafted subcutaneously into mice, resulting in increased serum testosterone levels. Our study will facilitate innovative therapeutic strategies for LOH syndrome.

## Highlights

• Developed a new protocol for generating hiPSC-derived Leydig-like cells
• Achieved 22-fold higher testosterone production compared to conventional methods
• Ensured over 16-week longevity and >99% differentiation efficiency
• Successful subcutaneous engraftment increased serum testosterone in mice

## Graphical abstract

**Figure:**
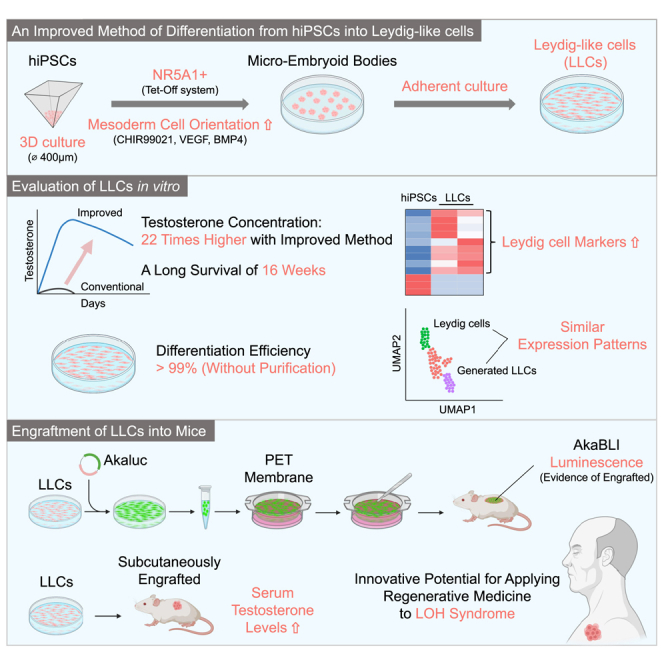

## Teaser

Aoi and workers present an innovative, efficient protocol for differentiating human iPSCs into Leydig-like cells capable of long-term stable testosterone secretion compared to conventional methods. This approach offers promising potential for regenerative therapies targeting late-onset hypogonadism syndrome.

## Introduction

The age-related decline in serum levels of the male hormone testosterone is a significant cause of late-onset hypogonadism (LOH) syndrome (Handelsman et al., 2015; Huhtaniemi, 2014; Wu et al., 2008). Clinical manifestations of LOH syndrome include muscle loss, a decreased bone mineral density, increased fat mass, sexual dysfunction, mood disorders, and fatigue, all of which lead to a diminished quality of life in older men (Basaria, 2014). LOH syndrome has also been associated with metabolic syndrome and diabetes (Stellato et al., 2000; Tsujimura et al., 2013) and is emerging as a significant public health concern. Testosterone is mainly secreted by Leydig cells located in the interstitium of the testes (Chamindrani Mendis-Handagama and Siril Ariyaratne, 2001). A decline in the number of Leydig cells with age leads to reduced blood testosterone levels, which in turn causes LOH syndrome (Mularoni et al., 2020).

Currently, testosterone replacement therapy (TRT) is the predominant treatment for LOH syndrome globally (Khera et al., 2016; Shin and Park, 2019). Although TRT effectively improves symptoms, it places a high compliance burden on patients, as its effects are not sustained, so regular treatment must be continued. For example, injectable therapy requires injections every 2–4 weeks (Bhasin et al., 2010). Skin patches cause skin rashes in many patients (Bhasin et al., 2010), and ointments may cause skin rashes and secondary exposure to others (Bhasin et al., 2010). Oral medications require high doses of testosterone due to metabolic effects, and there are concerns about the burden on the liver (Bhasin et al., 2010). For these reasons, alternative treatments to TRT are desirable.

The transplantation of Leydig cells is viewed as a promising novel therapeutic alternative to TRT. If the transplanted Leydig cells remain viable within the body, their efficacy can last for the lifetime of the recipient, or at least significantly longer than the transient efficacy of a single testosterone injection. However, Leydig cells, which are terminally differentiated and do not proliferate *in vitro* (Chen et al., 2009; Haider, 2004), face supply scarcity, hampering their potential use in cell transplantation procedures.

A previous study reported that targeted activation of *NR5A1, GATA4*, and *DMRT1* could convert human foreskin fibroblasts into functional Leydig-like cells (LLCs); however, its reprogramming efficiency was approximately 7% (Huang et al., 2019). In addition, a method for isolating human stem Leydig cells (hSLCs) from human testes and generating proliferating Leydig cells from these hSLCs has also been reported, but ethical issues surrounding the procurement of hSLCs and the clinical application of this method pose significant challenges (Feng et al., 2021).

Several groups, including ours, have investigated methods for generating testosterone-secreting LLCs from stem cells (Chen et al., 2019, Ishida et al., 2021; Ji et al., 2020; Li et al., 2019; Shin et al., 2021, Yang et al., 2015). Based on reports that *NR5A1* is a master regulator required for Leydig cell differentiation (Luo et al., 1994; Yang et al., 2015), we successfully generated functional testosterone-secreting LLCs by forced expression of *NR5A1* in human induced pluripotent stem cells (hiPSCs) and employed three-dimensional (3D) culture to form embryoid bodies (EBs) (Ishida et al., 2021). However, the generated cells could be maintained for approximately 7 weeks only, and given that the Tet-On system was utilized to maintain the expression of *NR5A1*, it was necessary to consistently administer doxycycline to the cells. Another group reported a differentiation induction method to induce differentiation of LLCs from hiPSCs without gene overexpression, using only the addition of compounds (Chen et al., 2019). However, the protocol consisted of six rather complex steps, and the generated cells produced a smaller amount of testosterone than our protocol, with the differentiation induction efficiency being low (about 50%). In addition, there are issues regarding the selection of cell transplantation sites. For example, if LLCs can be engrafted subcutaneously, the wound associated with transplantation would be small and simple to induce, and if the cells become cancerous, they could be easily removed. However, current methods of engraftment of cells into the interstitium of the testis (Chen et al., 2019; Curley et al., 2019; Yang et al., 2015, 2020) or into the peritoneal cavity (Feng et al., 2021) do not provide this advantage and thus present a significant challenge.

In this study, we established a novel and improved protocol that involves the use of a Tet-Off system (no need for doxycycline to maintain forced expression of exogenous genes) for the overexpression of *NR5A1* in hiPSCs, the addition of cytokine cocktails to enhance mesoderm differentiation, and the transitional shift from 3D to two-dimensional (2D) culture system. This approach has enabled us to generate LLCs that secrete testosterone at concentrations approximately 22 times higher than those achieved with the conventional method (Ishida et al., 2021), with these LLCs having a lifespan exceeding 16 weeks in culture dishes. We also successfully achieved an LLC differentiation efficiency of nearly 100% without the need for purification. Contrary to the previous approach of injecting LLCs into the testis, with a clinical perspective in mind, we also successfully elevated serum testosterone levels by engrafting clusters of LLCs into the subcutaneous tissues of castrated mice, an approach that is more readily applicable in clinical practice.

## Results

### Establishment of a novel method of LLC differentiation with high testosterone secretion and long-term viability

**Figure 1:**
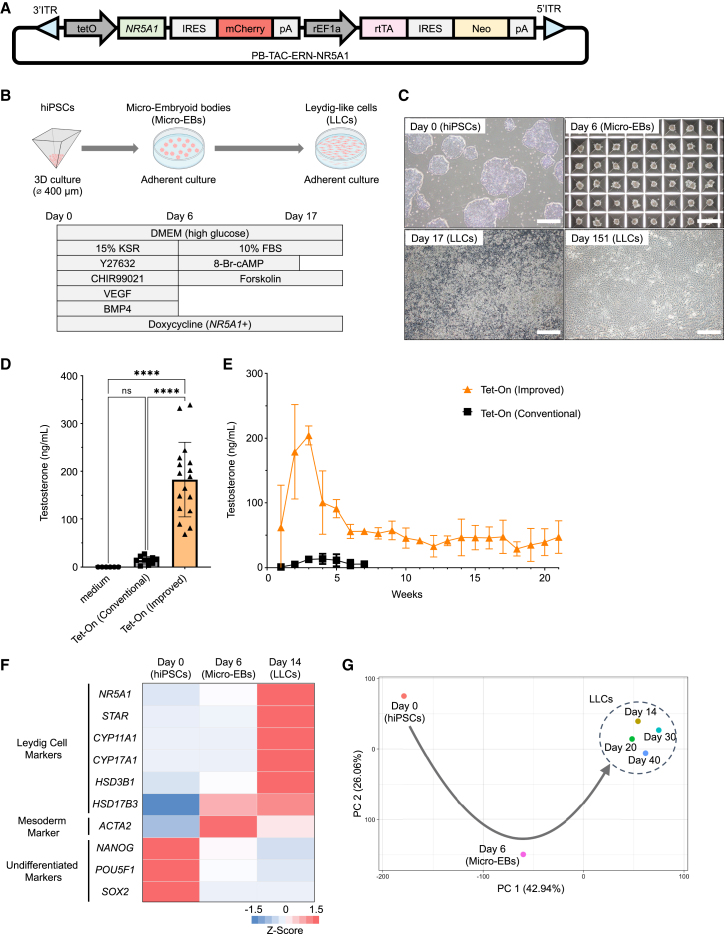
Improved method of generating hiPSC-derived LLCs (A) Doxycycline-inducible *NR5A1* piggyBac vector. *NR5A1* and mCherry were combined with an IRES such that when exogenous *NR5A1* was expressed, the fluorescent protein mCherry was co-expressed. (B) Schematic representation of the improved differentiation protocol for generating LLCs from hiPSCs. This protocol is characterized by the constitutive forced expression of *NR5A1*, switching from 3D to adherent culture, and addition of compounds to promote mesoderm differentiation. (C) Morphological changes in cells generated using the improved method of differentiation induction. Cells at day 0 (hiPSCs), day 6 (micro-EBs), day 17, and day 151 (LLCs) were observed using phase-contrast microscopy. Scale bars: 500 μm. (D) Testosterone concentrations in the culture supernatant were measured using the electrochemiluminescence immunoassay (ECLIA) method. Testosterone concentrations represent the peak levels observed during the measurement period. The testosterone concentration peaked value on days 21–37 (median: day 28) for the conventional method and on days 15–26 (median: day 18) for the improved method. The findings are expressed as the mean ± standard deviation (SD) from independent experiments, with sample sizes of *n* = 6 for the control (medium) group, *n* = 10 for the conventional method group, and *n* = 17 for the improved method group. Statistical significance is denoted by ^∗∗∗∗^ for *p* < 0.0001. (E) The comparison of changes in testosterone concentrations in culture supernatants during differentiation induction between the improved and the conventional methods. Mean value ± SD (*n* = 3 independent experiments). (F) A heatmap illustrating changes in the expression of Leydig cell markers, a mesoderm marker, and undifferentiated markers using NGS. The gene expression was depicted with color-coded representations derived from *Z* scores calculated from transcript per million (TPM) values. (G) A principal component analysis of NGS data from day 0 (hiPSCs), day 6 (micro-EBs), and LLCs (days 14, 20, 30, and 40) is presented. The horizontal axis represents the score of principal component 1, whereas the vertical axis represents the score of principal component 2.

Based on a previously reported protocol in which *NR5A1* was overexpressed using the Tet-On system (Figure 1A) (Ishida et al., 2021), we optimized the method using testosterone concentrations in the culture supernatant as an indicator by improving four points (1)–(4). The protocol of the improved method and the cell morphological changes during differentiation are shown in Figures 1B and 1C, respectively.

(1) Although we used a 96-well plate with a diameter of 7 mm per well from days 0 to 6 to form EBs in the conventional method (Figures S1A and S1B), we employed a plate with numerous open microwells 400 μm diameter to increase the number of micro-EBs generated in the improved method (Figures 1B and 1C).
(2) Because Leydig cells are mesodermal cells (Merchant-Larios and Moreno-Mendoza, 1998), we added CHIR99021, VEGF, and BMP4, which are known to enhance mesoderm differentiation (Ohta et al., 2016), from days 0 to 6.
(3) Contrary to the conventional method, which continued to culture EBs in 3D culture (Ishida et al., 2021), we intentionally transitioned the mesodermal lineage micro-EBs from 3D culture to adherent culture on day 6 (Figures 1B and 1C). The EBs then adhered to the bottom of the culture dish and lost their spherical shape, and each individual cell migrated and divided into the surrounding spaces (Video S1).
  Video S1. Differentiation of mesodermal embryoid bodies into LLCs Micro-EBs transitioned from a 3D culture to an adherent culture to induce differentiation into LLCs on day 6. Images were captured every 5 min using a time-lapse phase-contrast microscope. Scale bar: 200 μm.
(4) From day 6 onward, we supplemented the medium with 8-bromoadenosine-3′,5′-cyclic monophosphate (8-Br-cAMP) and forskolin, as in the conventional method (Figures 1B and S1A) (Ishida et al., 2021; Yang et al., 2015).

The LLCs generated by the improved method had an average peak testosterone concentration in the culture supernatant that was approximately 12.8 times higher than that of the LLCs generated by the conventional method (Figure 1D; Table S1). Unlike the LLCs derived from the conventional method, which died after approximately 7 weeks due to loss of their embryoid shape, those derived via the improved method survived on culture dishes for at least 21 weeks while secreting testosterone stably (Figure 1E).

### Comprehensive gene expression changes during the process of differentiation into LLCs and differentiation efficiency

Next, we analyzed the changes in comprehensive gene expression during the differentiation process from hiPSCs to LLCs in the current method using RNA sequencing. At the onset of differentiation induction (day 0), we observed the expression of undifferentiated marker genes, such as *POU5F1*, *SOX2*, and *NANOG* (Figure 1F). As differentiation proceeded, we detected a significant increase in the expression of Leydig cell markers, such as *STAR*, *CYP11A1*, *CYP17A1*, *HSD3B1*, and *HSD17B3*, by day 14. This result was consistent with the increased testosterone secretion in LLCs after day 14 of differentiation induction (Figure 1E). A principal component analysis (PCA) revealed that the LLCs generated on days 14, 20, 30, and 40 detected similar global gene expression patterns, suggesting that Leydig cell differentiation was likely accomplished by day 14 (Figure 1G).

To examine the characteristics of the genes that showed an upregulated expression in LLCs on day 14 compared to induced pluripotent stem cells (iPSCs), we selected 239 genes (higher expression in LLCs with a fold change >30) and performed a pathway analysis (Figures S2A and S2B; Table S2). This analysis for these genes suggested that, in addition to Leydig cell markers, the expression of genes related to steroidogenesis and androgen synthesis, which are characteristic functions of Leydig cells, increased.

Because of their pluripotency, iPSCs can differentiate into various cell types, including unintended cell types (Takahashi and Yamanaka, 2006). Therefore, it is essential to achieve high differentiation efficiency when generating specific cell types from iPSCs for clinical applications. Previous reports have shown that <50% of LLCs from human iPSCs express Leydig cell markers (Chen et al., 2019). Furthermore, even with our conventional differentiation induction protocol, the generated cell population produced not only testosterone but also aldosterone and cortisol, suggesting the presence of non-target cells, such as adrenal cortex cells (Ishida et al., 2021).

We performed immunofluorescence to determine the differentiation efficiency of LLCs in the cell population generated using the current method (Figures S2C and S2D). All of the examined biomarkers for Leydig cells (HSD17B3, LHCGR, STAR, and CYP17A1) showed high positivity rates, particularly HSD17B3 and LHCGR, with a positivity rate of over 99%. A flow cytometry analysis also revealed high positivity rates for biomarkers of Leydig cells, with LHCGR exhibiting the highest rate at 98.31% (Figures S2E and S2F). These results demonstrated that our current protocol can effectively differentiate hiPSCs into LLCs, resulting in a homogeneous cell population without the need for purification.

### Generation of LLCs using the forced expression of *NR5A1* via the Tet-Off system

**Figure 2:**
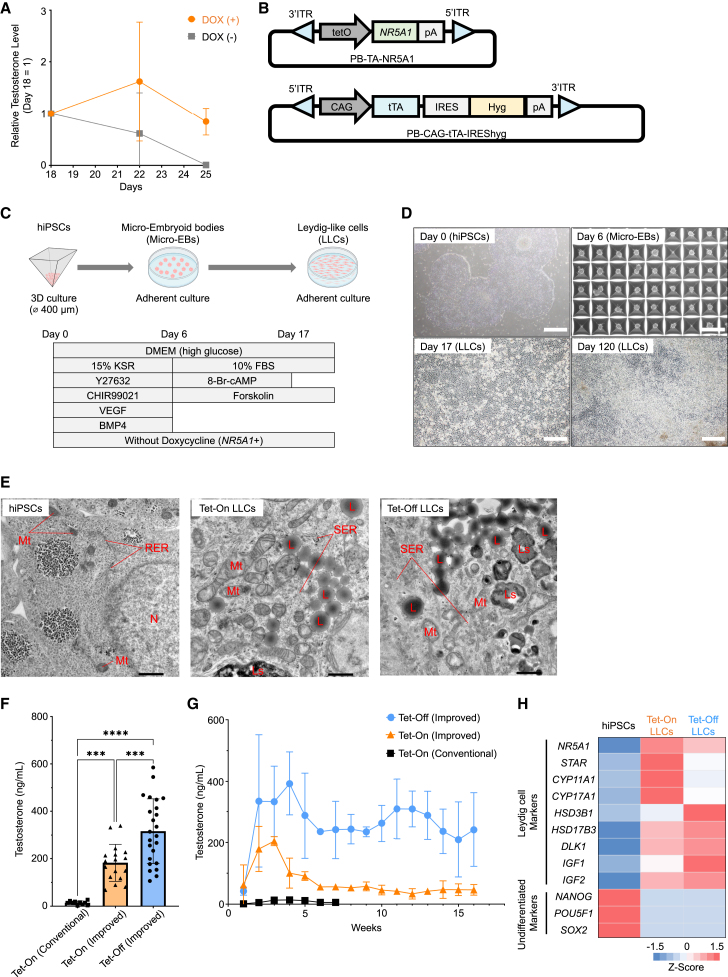
Generation of hiPSC-derived LLCs using the Tet-Off System (A) LLCs generated by the forced expression of *NR5A1* using the Tet-On system exhibited a decrease in testosterone production in the absence of doxycycline. In the DOX (+) group, 1.5 μM doxycycline was continuously added to the medium, whereas in the DOX (−) group, doxycycline was removed after day 18. The concentration of testosterone in the culture supernatant on day 18 is represented as a ratio of 1. Mean value ± SD (*n* = 3 independent experiments). (B) Construction of a piggyBac vector for the *NR5A1* expression in the absence of doxycycline (using the Tet-Off system). (C) A schematic representation of the protocol for inducing the differentiation of hiPSCs to LLCs is shown. To induce *NR5A1* expression using the Tet-Off system, doxycycline was removed during the differentiation-induction process. (D) Morphological changes in the cells were captured by phase-contrast microscopy on day 0 (hiPSCs), day 6 (micro-EBs), day 17, and day 120 (LLCs). Scale bars: 500 μm. (E) Electron micrographs of hiPSCs (left), Tet-On LLCs (middle), and Tet-Off LLCs (right) are shown. The abbreviations for the organelles are as follows: N, nucleus; Mt, mitochondria; L, lipid droplets; LS, lysosomes; SER, smooth endoplasmic reticulum; RER, rough endoplasmic reticulum. Scale bars: 1,000 nm. (F) The testosterone concentration in the culture supernatant was measured using the ECLIA method. Testosterone concentrations represent the peak levels observed during the induction period. Peak testosterone concentrations were recorded on days 21–37 (median: day 28) for the Tet-On conventional group, on days 15–26 (median: day 18) for the Tet-On improved group, and on days 18–90 (median: day 32) for the Tet-Off improved group. Results are represented as the mean ± SD from independent experiments, with sample sizes of *n* = 10 for the Tet-On conventional group, *n* = 17 for the Tet-On improved group, and *n* = 24 for the Tet-Off improved group. Statistical significance was denoted by ^∗∗∗∗^ for *p* < 0.0001 and ^∗∗∗^ for *p* < 0.001. (G) Testosterone concentrations in the culture supernatants during differentiation induction were compared between the groups. Mean value ± SD (*n* = 3 independent experiments). (H) A heatmap displays variations in expression of Leydig cell markers and undifferentiated markers using NGS, represented by color-coded scales derived from *Z* scores calculated from transcripts per million (TPM) values. The analysis included hiPSCs on day 0, Tet-On LLCs on day 30, and Tet-Off LLCs on day 35.

We previously generated LLCs by continuously expressing *NR5A1*, a master regulator for differentiation into Leydig cells (Luo et al., 1994; Yang et al., 2015), using the Tet-On system (Ishida et al., 2021). To investigate whether or not the continuous expression of *NR5A1* is necessary for LLCs to continue to secrete testosterone, we removed doxycycline from the culture medium after differentiation and examined the amount of testosterone produced. We monitored the expression of exogenous *NR5A1* by fluorescence observation of mCherry (Figure 1A) and confirmed a decrease in its expression 11 days after doxycycline removal (Figure S3A). In addition, the amount of testosterone also decreased and reached almost zero after 1 week (Figure 2A). These results suggest that *NR5A1* is essential not only for differentiation into Leydig cells but also for maintaining the Leydig cell function.

Considering the prospective application of LLCs in future transplantation therapies, it is undesirable to have to continue adding doxycycline in order to sustain the expression of *NR5A1*. Therefore, we opted to utilize the Tet-Off system to induce the expression of *NR5A1* without the addition of doxycycline (Figure 2B) (Gossen and Bujard, 1992). We attempted to induce differentiation into LLCs using the same protocol as the Tet-On system, except that doxycycline was added until the start of differentiation induction and then removed (Figures 1B and 2C). In differentiation using the Tet-Off system, micro-EBs were formed by day 6, and the cell morphologies of differentiated cells after switching to adherent culture were similar to those generated using the Tet-On system (Figures 1C and 2D). Previous reports have indicated that rat Leydig cells are characterized by well-developed organelles such as lipid droplets and smooth endoplasmic reticulum, reflecting their function in synthesizing testosterone from cholesterol (Chen et al., 2009). We performed electron microscopy to analyze three cell types: hiPSCs, LLCs generated by the Tet-On system (Tet-On LLCs), and LLCs generated by the Tet-Off system (Tet-Off LLCs). Electron microscopy showed that organelles such as lipid droplets (L) and smooth endoplasmic reticulum were abundantly present in the cytoplasm of both Tet-On LLCs (Figure 2E middle panel) and Tet-Off LLCs (Figure 2E right panel), but not in the hiPSC stage (Figure 2E left panel). These data suggest that LLCs are similar to Leydig cells in terms of their internal cellular structure.

Unexpectedly, the concentration of testosterone in the culture supernatant was significantly higher in Tet-Off LLCs than Tet-On LLCs, and the concentration of testosterone in the culture supernatant of Tet-Off LLCs was approximately 22 times higher than that of LLCs generated by our conventional method (Table S1; Figure 2F). Testosterone secretion by 1 million Tet-Off LLCs surpassed 200 ng over 24 h (Figure S3B). In addition, Tet-Off LLCs demonstrated the ability to survive for more than 16 weeks while secreting large amounts of testosterone on culture dishes (Figure 2G).

We also performed semiquantitative reverse-transcription polymerase chain reaction to examine the expression of Leydig cell marker genes in Tet-On and Tet-Off LLCs (Figure S3C). The results showed that both LLCs expressed steroidogenic enzyme genes (*STAR*, *CYP11A1*, *CYP17A1*, *HSD3B1*, and *HSD17B3*), all of which are required for the synthesis of steroid hormones from cholesterol. A comprehensive gene expression analysis using next-generation sequencing (NGS) detected the expression of Leydig cell markers in LLCs generated by both the Tet-On and Tet-Off systems (Figure 2H). A WikiPathways analysis was employed to examine the 239 genes that exhibited an increased expression in Tet-Off LLCs compared to that in hiPSCs (Figures S3D and S3E; Table S2). The results revealed that the pathway related to “androgen synthesis” was the top pathway, which was consistent with the analysis of Tet-On LLCs (Figure S2B). A PCA showed that Tet-On and Tet-Off LLCs were located close to each other, indicating that their comprehensive gene expression patterns were similar (Figure S3F). There were subtle differences between Tet-On and Tet-Off LLCs in each experiment (Figures 2F–2H), which may be due to variations in *NR5A1* gene transfer efficiency and promoter activity between the experimental systems. These variations likely resulted in the differences in the expression levels of various Leydig cell markers and testosterone concentrations.

### Single-cell RNA sequencing revealed similarities between the generated LLCs and authentic Leydig cells

**Figure 3:**
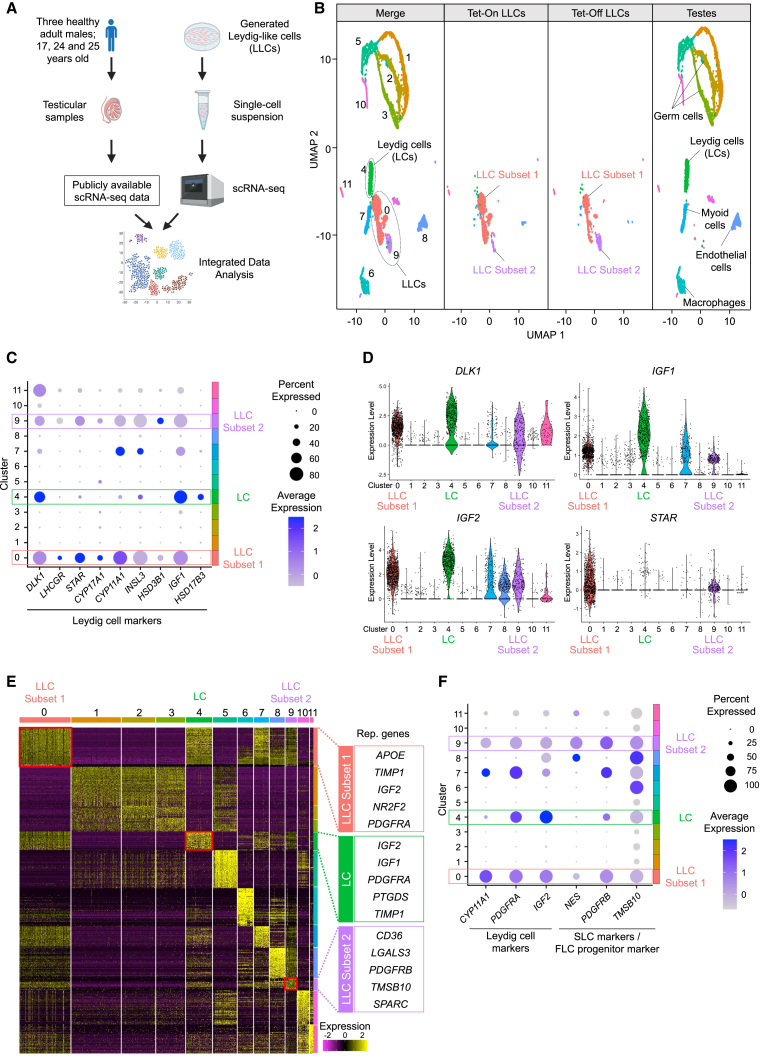
Genetic analyses of LLCs using scRNA-seq (A) Steps for single-cell analyses: the data acquired from the generated LLCs were merged with previously published testicular data from healthy males, and the integrated data were then analyzed. (B) UMAP plots were created from the integrated data to visualize the distribution of the gene expression profiles. Presented from left to right are the UMAP plots of the integrated data, Tet-On LLCs on P0 day 14, Tet-Off LLCs on P2 day 18, and testes. (C) Gene expression characteristics of Leydig cells were compared among the identified clusters and represented using dotplots. (D) Gene expression characteristics of Leydig cells were compared among the identified clusters and represented using violin plots. (E) A heatmap was used to visualize the gene characteristics of each cluster, with the vertical axis representing genes and the horizontal axis corresponding to the clusters. Genes with an elevated expression appear yellow, whereas those with a decreased expression appear purple. Representative genes that are characteristically expressed in the LC, LLC subset 1, and LLC subset 2 clusters are listed on the right side (see also Table S3). (F) Genes differentially expressed between LLC subsets 1 and 2 were identified and visualized using dotplots. The Leydig cell markers *CYP11A1*, *PDGFRA*, and *IGF2* were highly expressed in subset 1. In contrast, the SLC markers *NES* and *PDGFRB*, as well as *TMSB10*, a gene involved in fetal Leydig cell (FLC) differentiation, were upregulated in subset 2.

To assess the molecular similarity of our generated Tet-On and Tet-Off LLCs to authentic Leydig cells, we performed a single-cell RNA sequencing (scRNA-seq) analysis. We integrated publicly accessible scRNA-seq data from testicular samples obtained from three healthy male subjects using scRNA-seq data from our generated cells (Figure 3A). Using the integrated dataset, we generated uniform manifold approximation and projection (UMAP) plots and then identified cell clusters based on marker genes distinctive of Leydig cells (cluster 4), myoid cells (cluster 7), macrophages (cluster 6), germ cells (clusters 1, 2, 3, 5, and 10), and vascular endothelial cells (cluster 8) (Figures 3B and S4A). Our generated Tet-On and Tet-Off LLCs were divided into LLC subset 1 (cluster 0) and LLC subset 2 (cluster 9), which were close to authentic Leydig cells on UMAP, indicating their similar gene expression patterns. We selected genes characteristic of Leydig cells and illustrated the expression status of these genes in each cluster using a dotplot (Figure 3C) and violin plots (Figure 3D). The expression patterns of Leydig cell marker genes in LLC subsets 1 and 2 were similar to those of authentic Leydig cells. The heatmap further demonstrated concordance in the gene expression status between LLCs and authentic Leydig cells (Figure 3E).

We also extracted the top 50 most distinctive genes for each cluster (Table S3), and the representative genes are shown in Figure 3E right panels. In LLC subset 1, genes such as *TIMP1*, *IGF2*, and *PDGFRA* as well as authentic Leydig cells exhibited a high expression, whereas markers such as *PDGFRB* (Li et al., 2022a; Odeh et al., 2014), a marker for stem Leydig cells (SLCs), and *TMSB10* (Inoue et al., 2022), a marker for fetal Leydig cell (FLC) progenitors, were elevated in LLC subset 2. In addition, when we examined the genes that showed a differential expression between LLC subsets 1 and 2, we found that the expression of *NES*, a characteristic marker of SLCs (Davidoff et al., 2004; Jiang et al., 2014; Yao et al., 2022), was higher in subset 2 than in subset 1 (Table S4; Figure S4B). A dotplot analysis showed that the expression of *PDGFRB*, *NES*, and *TMSB10* was more prominent in LLC subset 2 than in subset 1 (Figure 3F). We compared the publicly available single-cell analysis data of human fetal testicular cells (Chitiashvili et al., 2020) with the data from LLCs, and UMAP showed that these cell groups were positioned apart (data not shown). Although LLCs expressed SLC markers and FLC progenitor markers more than Leydig cells (Figure 3F), we found that the gene expression profiles of fetal testicular cells and LLCs were not identical. We performed a dotplot analysis to further investigate the markers related to cell division. It revealed that proliferation markers, such as *MKI67* and *PCNA*, were expressed at higher levels in LLCs than in authentic Leydig cells (Figure S4C). The top 100 genes that were differentially expressed between LLCs and Leydig cells were identified (Table S4). A pathway analysis based on this gene list revealed that when compared to Leydig cell, pathways related to steroidogenic enzymes were significantly upregulated in LLC subset 1, and pathways related to cell division were significantly upregulated in LLC subset 2.

In addition, we analyzed cell samples at each time point of differentiation induction (on days 0, 6, 14, and 28) and created a UMAP (Figure S4D). As differentiation induction progressed, *DLK1*, a Leydig cell marker gene, was upregulated (Figure S4E). We also assessed the expression of *SOX17* (an endodermal marker), *ACTA2* (a mesodermal marker), and *SOX1* (an ectodermal marker). We observed that *SOX17* and *SOX1* were not expressed during differentiation induction, whereas *ACTA2* expression was increased on day 6, which was consistent with the results of the NGS analysis (Figure 1F). Given that Leydig cells are derived from the mesoderm, upregulation of *ACTA2* expression during differentiation induction is biologically plausible.

### Measurement of the differentiation efficiency and undifferentiated marker genes in Tet-Off LLCs and the analysis of passaging and freeze-thaw cycles

**Figure 4:**
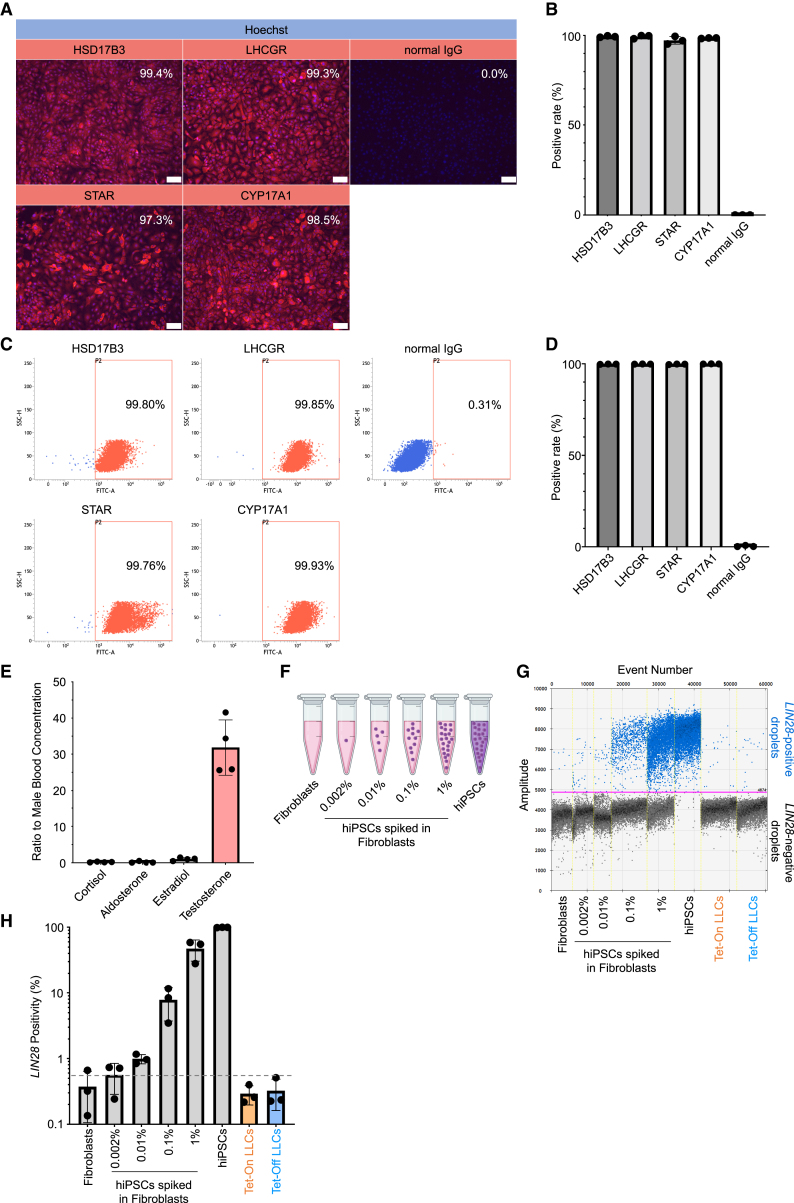
Evaluation of Tet-Off LLCs by immunostaining, flow cytometry, and ddPCR (A) Representative images for the expression of Leydig cell markers in differentiated LLCs on day 7 after two passages using immunofluorescence assays. Scale bars: 100 μm. (B) Statistical analyses of the immunofluorescence assay. Positivity was quantitatively evaluated using the ImageJ software program. Mean value ± SD (*n* = 3 independent experiments). (C) Representative flow cytometry histograms for Leydig cell markers in differentiated LLCs on day 4 after one passage. (D) Statistical analyses of flow cytometry. Mean value ± SD (*n* = 3 independent experiments). (E) Cortisol, aldosterone, estradiol, and testosterone concentrations were measured in the culture supernatant on days 38–42 of cell culture. These concentrations were divided according to the corresponding upper reference limits of male blood concentrations. Mean value ± SD (*n* = 4 independent experiments). (F) Detection of *LIN28A* using ddPCR. hiPSCs were used as positive controls, while fibroblasts were used as negative controls. Fibroblast samples were mixed with increasing concentrations of hiPSCs (0.002%, 0.01%, 0.1%, and 1%). (G) Quantification of positive droplets using ddPCR. The magenta line indicates the fluorescence positivity boundary of the droplet, classifying the droplet as *LIN28A* positive or *LIN28A* negative. The blue dots represent droplets containing at least one copy of *LIN28A* being evaluated. (H) Statistical analyses of the ddPCR assay. The vertical axis represents the percentage of *LIN28A*-positive droplets. The dotted line indicates the rate of *LIN28A* positivity in the samples containing 0.002% hiPSCs. Mean ± SD, *n* = 3 independent experiments.

As in the Tet-On system, we investigated whether or not the induction efficiency was also high when the Tet-Off system was used. Both immunofluorescence (Figures 4A and 4B) and flow cytometry analyses (Figures 4C and 4D) showed that the percentage of cells expressing the Leydig cell markers exceeded 97%, indicating that even with the forced expression of *NR5A1* by the Tet-Off system, hiPSCs can be effectively differentiated into homogeneous LLC populations without further purification. Furthermore, the cells generated using the conventional method contained aldosterone and cortisol in their culture supernatant (Ishida et al., 2021). In contrast, the LLCs generated by the current method secreted little aldosterone or cortisol in their culture supernatants. The testosterone concentration in the supernatant was approximately 30-fold higher than the normal level in healthy male blood, whereas the concentrations of cortisol, aldosterone, and estradiol were 0.19, 0.17, and 1.01 times higher than the normal levels in healthy male blood, respectively (Table S5; Figure 4E). These results indicate that our current method induces the expression of *NR5A1* in hiPSCs while promoting intensive differentiation into Leydig cells without concomitant differentiation into adrenocortical cells or other gonadal cells.

The presence of residual undifferentiated cells within the final cellular product derived from hiPSCs can lead to uncontrolled proliferation and formation of teratomas (Knoepfler, 2009; Koyanagi-Aoi et al., 2013). To quantify the presence of undifferentiated cells within the generated cell population, we employed the *LIN28A*/digital droplet PCR (ddPCR) method (Kuroda et al., 2015) using *LIN28A* as a marker for undifferentiated cells (Yu et al., 2007). As in previous reports, fibroblasts were used as negative controls for *LIN28A*, and hiPSCs were used as positive controls (Kuroda et al., 2015). Fibroblast samples as negative controls were mixed with increasing concentrations of hiPSCs (0.002%, 0.01%, 0.1%, and 1%, Figure 4F). The results demonstrated that the *LIN28A*-positive droplet rates of both the generated Tet-On LLCs and Tet-Off LLCs were below the 0.002% threshold for the hiPSC contamination rate, indicating a low risk of teratoma formation after transplantation (Figures 4G and 4H).

In our current method, EBs derived from hiPSCs lose their shape after transition from a 3D culture to an adherent culture, and LLCs reach confluence at least 17 days after the start of induction. To examine whether or not the generated LLCs could be passaged and expanded while maintaining their function, we performed passaging after the cells reached confluence (Figure S5A). LLCs were able to proliferate while maintaining their cell morphology and testosterone secretion function for at least three passages (Figures S5B–S5D). Furthermore, we confirmed that LLCs could also be cryopreserved, thawed, and cultured again while maintaining their testosterone secretion (Figure S5E). When considering clinical applications, these features may facilitate the storage of cells intended for transplantation, making it possible to treat these cells as off-the-shelf products.

### Response of LLCs to gonadotropic hormones

We assessed whether the addition of gonadotropic hormones, such as luteinizing hormone (LH) and human chorionic gonadotropin (hCG), to the culture supernatant would increase testosterone secretion by LLCs. The testosterone concentration secreted within 3 h after the addition of LH or hCG showed no significant difference from the control in both Tet-On LLCs (Figure S6A) and Tet-Off LLCs (Figure S6B). Thus, we conclude that our induced LLCs secrete testosterone autonomously, without regulation by gonadotropic hormones. To investigate why LHCGR does not respond to the gonadotropic hormone LH, even though the expression is detected, we considered the possibility that LHCGR, the receptor for LH and hCG, is not localized on the cell surface. We performed immunostaining for LHCGR, with or without membrane permeabilization. The results indicated that nearly 100% of the cells were positive for LHCGR when permeabilized, whereas the percentage of LHCGR-positive cells decreased to approximately 30% without permeabilization (Figures S6C and S6D). These findings suggest that the limited expression of LHCGR on the cell surface leads to the inability of LLCs to respond to gonadotropic hormones.

### Engraftment of LLCs into immunocompromised mice

**Figure 5:**
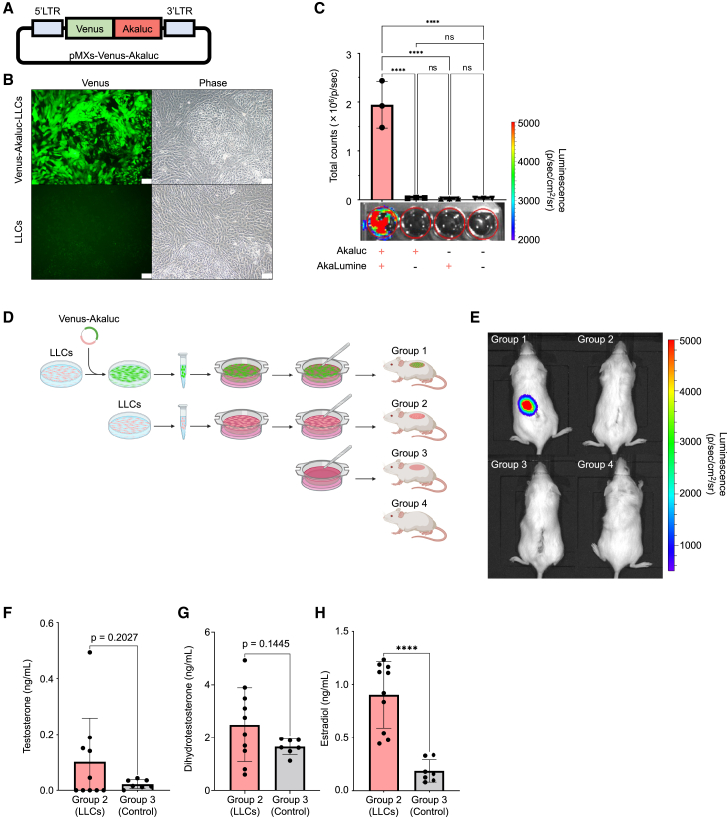
Transplantation of LLCs into immunodeficient mice (A) Diagram of a retroviral vector to introduce Venus-Akaluc expression into Tet-Off LLCs. (B) Fluorescence (left) and phase-contrast (right) images of LLCs after retroviral transduction with Venus-Akaluc (upper) and control LLCs (lower). Scale bars; 100 μm. (C) When the substrate AkaLumine was added to the culture wells of LLCs transfected with Venus-Akaluc, luminescence of AkaBLI was detected after 3 min by the substrate enzyme reaction. Images of each group are shown. Quantification of the AkaBLI luminescence was performed in each well. Total counts are the sum of all counts for all pixels inside the region of interest (ROI), presented as the mean ± SD, *n* = 3 independent experiments. Statistical significance is denoted by ^∗∗∗∗^ for *p* < 0.0001. (D) A schematic diagram of the cell transplantation process in mice is shown. Group 1 mice were transplanted subcutaneously using PET membranes to which Venus-Akaluc-transfected LLCs were attached; group 2 mice were transplanted subcutaneously using PET membranes to which Venus-Akaluc-untransfected LLCs were attached; group 3 mice were implanted subcutaneously with PET membranes alone, with no transplanted cells; and group 4 mice did not have any implants. (E) Three days after transplantation, the substrate AkaLumine was injected intraperitoneally in mice; 10 min later, AkaBLI luminescence was detected subcutaneously in mice in the Venus-Akaluc-LLC live-transplantation group (group 1). Images of each group are shown. *n* = 3 independent experiments. (F–H) The results of the blood hormone levels taken 3 days after transplantation are shown for both the group of mice in which LLCs were adhered to the PET membrane and then transplanted (group 2 in Figure 5D) and the control group that received only the membrane transplant (group 3). Statistical significance was denoted by ^∗∗∗∗^ for *p* < 0.0001. Data are presented as mean ± SD from independent experiments, with a sample size of *n* = 10 for group 2 and *n* = 7 for group 3.

To investigate whether or not the induced LLCs function as testosterone-secreting cells *in vivo*, we transplanted them into immunodeficient mice. First, we suspended the cells in medium, injected them subcutaneously or intraperitoneally, and measured the blood testosterone concentration. However, no significant increases were observed. Next, we generated LLCs that expressed the fluorescent protein Venus (Nagai et al., 2002) and the bioluminescent enzyme Akaluc (Iwano et al., 2018) to visualize whether or not LLCs were engrafted *in vivo* by retroviral transduction (Figure 5A). LLCs transduced with Venus-Akaluc exhibited distinctive green fluorescence (Figure 5B). Akaluc-transfected LLCs showed a bioluminescent signal, AkaBLI, only when an AkaLumine-HCl substrate was added to the culture medium (Figure 5C).

Because the LLCs were cultured in an adherent manner, to create a similar environment *in vivo*, we attempted to seed LLCs onto a polyethylene terephthalate (PET) membrane and transplanted them together (Figure 5D). Mice in group 1 were transplanted with PET membranes carrying LLCs transfected with Venus-Akaluc, whereas mice in group 2 were transplanted with PET membranes carrying LLCs that lacked Venus-Akaluc. Mice in group 3 received only PET membranes, lacking any cells, and mice in group 4 received neither cells nor PET membranes (Figure 5D). The LLCs consistently exhibited green fluorescence for at least 2 days, even after being attached to the PET membrane (Figure S6E), and were then transplanted subcutaneously into mice. Three days after transplantation, the AkaLumine-HCl substrate was intraperitoneally injected into the mice, resulting in the observation of a bioluminescent signal coincident with the transplantation site (Figure 5E). In the culture supernatant of group 1 LLCs, testosterone levels were significantly lower than those in group 2 LLCs (data not shown). It is hypothesized that this reduction may be due to cell damage incurred during retroviral introduction of Venus-Akaluc into LLCs. Consequently, the focus was shifted to group 2 grafts, which are intended to increase serum testosterone levels after transplantation into mice. Transplantation of group 2 LLCs into female mice tended to increase the levels of serum testosterone and its metabolite, dihydrotestosterone (DHT), whereas a significant increase was observed in estradiol, another metabolite of testosterone (Table S5; Figures 5F–5H). Upon excision of the skin and subsequent observation, subcutaneous angiogenic vessels were observed at the engraftment site, suggesting that an environment had been established in which testosterone secreted by LLCs could easily circulate into the mouse bloodstream (Figure S6F). Angiogenesis was observed at the site of implantation, even when the artificial membrane was implanted alone (group 3, data not shown), suggesting that angiogenesis was not induced by the cells. Furthermore, immunostaining of PET membranes removed from mice revealed the presence of cells positive for LHCGR, a marker of Leydig cells (Figure S6G). In summary, LLCs adhering to PET membranes were viable *in vivo*, with blood levels of testosterone not showing significant elevation, whereas levels of metabolites were significantly elevated.

**Figure 6:**
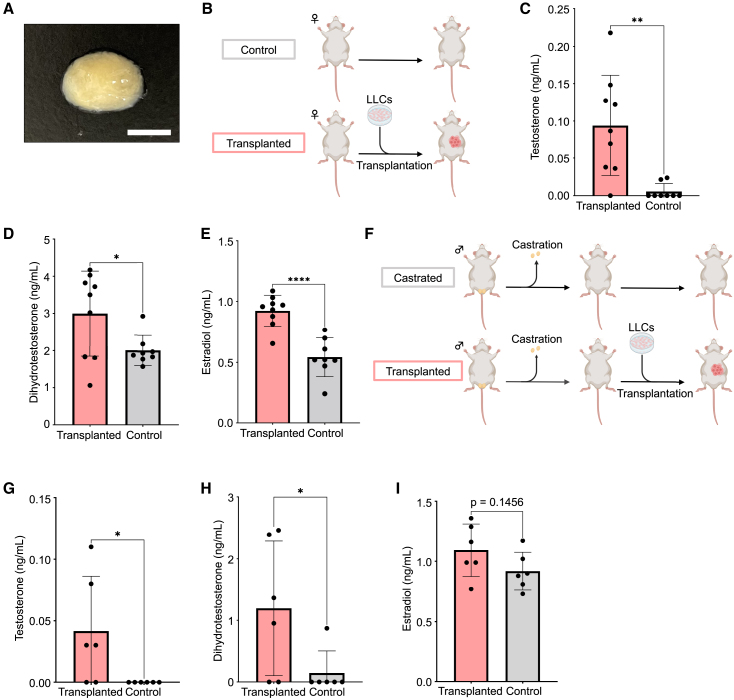
Transplantation of LLCs as cell clusters into mice (A) LLC clusters were formed by removing the cells from the culture dish using a cell scraper. Scale bar: 5 mm. (B) LLCs were transplanted subcutaneously into immunocompromised female mice as clusters. No cell transplantation was performed in the control mice. (C–E) Serum testosterone, dihydrotestosterone, and estradiol levels were measured 3 days after cell transplantation. Statistical significance was denoted by ^∗∗∗∗^ for *p* < 0.0001, ^∗∗^ for *p* < 0.01, and ^∗^ for *p* < 0.05. Data are presented as the mean ± SD from independent experiments, with sample sizes of *n* = 9 for the transplantation group and *n* = 8 for the control group. (F) LLC clusters were transplanted subcutaneously into immunocompromised castrated male mice. No cell transplantation was performed in control mice. (G–I) Four days after transplantation, the serum testosterone, dihydrotestosterone, and estradiol levels were measured. Statistical significance was denoted as ^∗^ for *p* < 0.05. Data are presented as the mean ± SD from independent experiments, with sample sizes of *n* = 6 for each group.

Next, we attempted to peel off the LLCs with a cell scraper (Figure 6A) and subcutaneously transplanted them into immunocompromised female mice as cell clusters without using a PET membrane (Figure 6B). Three days later, a significant increase in the serum testosterone level was observed (Figure 6C). Blood levels of its metabolites, DHT and estradiol, were also significantly increased in the transplantation group (Table S5; Figures 6D and 6E, respectively). Furthermore, we transplanted LLCs into immunocompromised castrated male mice using the same method (Figure 6F). Relative to the castrated group, which served as the negative control, the LLC transplantation group showed a significant increase in serum testosterone and DHT levels, with a tendency toward increased estradiol levels (Table S5; Figures 6G–6I). The blood testosterone levels were 0 ± 0 ng/mL in the castrated group, 0.04 ± 0.04 ng/mL in the LLC-transplanted group, and 0.83 ± 0.55 ng/mL in the non-castrated group. These findings indicated that LLCs, when subcutaneously engrafted into immunocompromised mice, are viable *in vivo* and secrete testosterone.

## Discussion

In this study, we successfully generated LLCs that produced testosterone at concentrations approximately 22 times higher than those achieved with our previous method (Ishida et al., 2021). The testosterone secretion was approximately 245 ng per million cells over 24 h, exceeding the highest reported value of approximately 70 ng for human LLCs derived from SLCs (Feng et al., 2021). These findings imply that LLCs generated with our current protocol exhibit exceptional testosterone secretion ability. Furthermore, our LLCs were able to be maintained *in vitro* for more than 16–21 weeks while continuously secreting testosterone, whereas those in a previous report could only be maintained for a maximum of 7 weeks (Ishida et al., 2021; Li et al., 2022b). With the aim of achieving LLC transplantation therapy, LLCs capable of sustained secretion of large amounts of testosterone would be exceedingly useful, as they would not only reduce the number of cells needed but also extend the interval between transplants. Although significant improvements were made in comparison to conventional methods for generating LLCs, there is still room for further optimization regarding EB size and cytokine concentrations.

At present, the mechanism underlying this prolonged viability remains unclear, although the existence of a subset within our LLCs exhibiting molecular signatures akin to earlier-stage Leydig cells, such as FLCs and SLCs, as revealed by an scRNA-seq analysis, might hint at one of the mechanisms underlying their sustained long-term viability. A further investigation is warranted to determine whether or not LLCs can be maintained over the long term *in vivo*.

Although the differentiation efficiency of stem cells to LLCs was reported to peak at 50% (Chen et al., 2019), our improved method achieved a differentiation efficiency of 99% without the need for any purification process, which simplifies the manufacturing process for clinical application. Furthermore, the expression of the undifferentiated gene marker *LIN28* in the resultant cell population was below a certain threshold. While a negative *LIN28A*/ddPCR test result does not solely indicate safety, the absence of a certain threshold of undifferentiated cells is among the fundamental quality attributes crucial for ensuring safety, thus indicating a reduced risk of teratoma formation after transplantation (Knoepfler, 2009; Koyanagi-Aoi et al., 2013). In addition, our LLCs can be cryopreserved and retain their testosterone-secreting capacity after thawing, which may be advantageous for clinical application. Although the issue of potential contamination of undifferentiated cells and cryo-preservability have been significant concerns in the clinical applications of LLCs, prior reports on Leydig-like cells derived from pluripotent stem cells (Chen et al., 2019; Ishida et al., 2021; Li et al., 2019) have not addressed these issues. Consequently, whether or not our methodology surpasses the previously reported methods in this regard remains uncertain, but the current method still addresses these concerns.

In previous reports, the standard method for engrafting generated LLCs involved transplantation into the abdominal cavity or testicular interstitial tissue of mice or rats (Chen et al., 2019, Feng et al., 2021; Jiang et al., 2014). However, in terms of clinical application, these cell transplantation methods have issues related to invasiveness, procedural complexity, and challenges in cell removal when required. To overcome these issues, the subcutaneous transplantation of LLCs is ideal. SLCs were reportedly successfully extracted from 6-week-old mice and ectopically transplanted into the subcutaneous tissue, resulting in an increase in serum testosterone levels (Arora et al., 2019). However, the main issues with that approach were that the experiment was performed using mouse cells, not human cells, and that the SLCs were extracted from young animals and then transplanted back into the same animals. Furthermore, Sertoli and myoid cells were required for cellular engraftment, as Leydig cells alone were insufficient, and these cells had to be engrafted simultaneously. In contrast, in our study, subcutaneous transplantation of the cells was performed using human cells, successfully engrafting LLC cells alone without the support of Sertoli or myoid cells, which also resulted in increased serum testosterone levels. We believe that these results are significant in that they represent a successful transplantation method with consideration for the clinical application of LLC in patients with LOH syndrome.

Several limitations associated with the present study warrant mention. First, our improved method forcibly expressed *NR5A1* to generate LLCs, but stopping the forced expression of *NR5A1* rendered the LLCs unsustainable and incapable of secreting testosterone (Figure 2A). Although *NR5A1* has traditionally been considered essential for Leydig cell differentiation (Luo et al., 1994; Yang et al., 2015), it may also be a critical gene for maintaining the cells after differentiation completion. The Tet-Off system employed in this study can eliminate the need for the addition of doxycycline, but the expression of endogenous *NR5A1* would help promote enduring homeostasis in Leydig-like cells through mechanisms identical to those observed in authentic Leydig cells.

Second, the concentration of testosterone secreted by LLCs showed variability among the experimental replicates (Figures 1D and 2F). Although the cause of this variability is unclear, it is generally accepted that during the induction of differentiation using pluripotent stem cells, numerous cells undergo dynamic changes, leading to differences in the timing of differentiation among individual cells. Additionally, factors such as cell density, distance between cells, nutrient depletion, and waste accumulation vary among experiments and cannot be precisely controlled. Therefore, controlling this variability among experiments and producing a large quantity of stable quality LLCs remain a challenge to be addressed in future clinical applications.

Third, although we added gonadotropic hormones, such as LH and hCG, to the culture medium, the LLCs did not respond (Figures S6A and S6B) and continued to secrete testosterone autonomously, regardless of the effects of such gonadotropic hormones. Therefore, it is desirable to develop LLCs that can physiologically control testosterone secretion and reproduce diurnal variations in the response to gonadotropic hormones. In this study, the expression of LHCGR was confirmed in more than 99% of LLCs by immunostaining and flow cytometry (Figures 4A–4D and S2C–S2F); however, without membrane permeabilization, the positive rate was only approximately 30% (Figures S6C and S6D). If LHCGR is stably expressed on the cell membrane surface, the responsiveness to gonadotropic hormones can be overcome. As LLCs did not respond to the addition of LH or hCG *in vitro*, we did not conduct experiments involving the addition of gonadotropic hormones after transplantation into mice. This issue remains a topic for future research.

Fourth, we have not yet determined whether our LLCs express HSD17B3 protein at levels comparable to those in authentic Leydig cells, as we were unable to isolate or culture primary testicular Leydig cells to be used as positive controls for immunostaining or flow cytometry. The mRNA expression level of *HSD17B3* was lower in LLCs than in testicular Leydig cells; however, it is known that in some cases, a protein’s expression level can be high even when its mRNA expression is low (Kosti et al., 2016). Therefore, it remains unclear whether the HSD17B3 protein expression in LLCs is actually low. To determine whether LLCs have functions comparable to those of Leydig cells, it is essential to clarify whether the expression levels of *HSD17B3*, a marker for Leydig cells and an important enzyme in the final stage of testosterone biosynthesis, are sufficient.

Fifth, maintenance of LLCs requires the addition of forskolin. Because forskolin is a plant-derived substance, it is necessary to generate LLCs that do not require this substance.

Sixth, because the mice used for transplantation in this study were immunodeficient, it will be necessary to encapsulate LLCs in an immunoisolation device and engraft them into immunocompetent animals in future studies. Regarding the transplantation of endocrine cells, significant device development has occurred in the field of pancreatic beta cells (Shahjalal et al., 2018). The primary method for controlling rejection without immunosuppressive drugs involves encapsulating islets within a device made of biocompatible materials, including alginate, and transplanting them subcutaneously or intraperitoneally (An et al., 2018; Orive et al., 2015; Vegas et al., 2016). However, none of these approaches have yet been put to practical use. In our study, transplantation of LLCs as clusters significantly increased blood testosterone levels in mice. Embedding cell clusters in alginate may thus be a promising approach. Furthermore, to our knowledge, the method used in this study, whereby endocrine cells are attached to an artificial PET membrane and the resulting cell sheets are grown subcutaneously, is novel, and this approach is promising for clinical applications. By combining the findings from our cell transplantation experiments with the techniques and knowledge accumulated in relation to β-cell transplantation, it may be possible to determine the optimal method for LLC engraftment.

If these challenges can be overcome, regenerative medicine for LOH syndrome will become a reality.

## Experimental procedures

### Improved method for induction of differentiation into LLCs

Anti-adherence rinsing solution (STEMCELL Technologies, Cambridge, MA, USA) was added to AggreWell400 (6-well plates; STEMCELL Technologies) at a volume of 2 mL/well. The plates were then centrifuged at 1300×*g* for 5 min in a plate centrifuge, after which the rinsing solution was removed through aspiration. Confluent 3AB4-*NR5A1*-hiPSCs were detached from culture dishes using 0.5X TrypLE Select. A total of 1.4 × 10^6^ viable cells were suspended in 5 mL of Step 1 medium (DMEM high glucose with 15% KSR, CHIR99021 [4 μM; Tocris Bioscience], BMP4 [80 ng/mL; R&D Systems, Minneapolis, MN, USA], VEGF [80 ng/mL; R&D Systems], CultureSure Y-27632 [10 μM], penicillin [50 units/mL], streptomycin [50 μg/mL], and doxycycline hyclate [1.5 μM]) and placed in a single well of rinsing solution-treated AggreWell400 plate. After centrifugation at 100×*g* for 3 min in a plate centrifuge, the cells were incubated at 37°C, 5% CO_2_ for 6 days to form micro-EBs. The medium was not altered during this 6-day period. Micro-EBs were then collected in tubes and centrifuged at 100×*g* for 3 min. The supernatant was aspirated, and the micro-EBs were suspended in 4 mL of Step 2 medium (DMEM high glucose with 10% FBS, 8Br-cAMP [1 mM], forskolin [100 μM], penicillin [50 units/mL], streptomycin [50 μg/mL], and doxycycline hyclate [1.5 μM]) and distributed into 2 wells of a Nunc cell culture-treated multidish (6-well plate; Life Technologies). The cells were maintained at 37°C, 5% CO_2_, and the medium was changed to Step 2 medium every 2–3 days. From day 17 of differentiation induction, the cells were sustained in Step 2 medium without 8Br-cAMP.

## Resource availability

### Lead contact

Further information and requests for resources and reagents should be directed to and will be fulfilled by the lead contact, Takashi Aoi (takaaoi@med.kobe-u.ac.jp).

### Materials availability

This study did not generate new unique reagents.

### Data and code availability

The datasets generated during the current study are available in the Gene Expression Omnibus database.

(1) NGS Data: GSE244796 Go to https://www.ncbi.nlm.nih.gov/geo/query/acc.cgi?acc=GSE244796.
(2) scRNA-seq Data: GSE245553 Go to https://www.ncbi.nlm.nih.gov/geo/query/acc.cgi?acc=GSE245553.

The source code for the NGS and scRNA-seq analyses presented in this study is available at https://github.com/KatsuyaSato1212/Sato-et-al.--RStudio_code.git.

## Acknowledgments

We thank all members of the laboratory for their scientific discussions, Yoko Matsuoka for administrative support, and Miwako Watanabe for technical support. We also thank Ryoko Hirohata and Michiko Nakamura for the plasmid construction. Naoya Hosono provided support for the scRNA-seq analysis. This work was supported by a grant from the 10.13039/100018627Research Center Network for Realization of Regenerative Medicine (JP18bm0704005h0003 and JP21bm0404051h0003) (to T.A. and M.K.-A.) from the 10.13039/100009619Japan Agency for Medical Research and Development (AMED), Akira Sakagami Fund for Research and Education, Kobe University Graduate School of Medicine (to T.A. and M.K.-A.), and 10.13039/501100001691JSPS KAKENHI (JP20K09578 [to M.F. and T.A.], JP23H03037 [to M.F., T.A., and K.S.], JP24K12483 [to K.S., M.F., and T.A.], and JP17K07256 [to K.W.]).

## Author contributions

K.S. designed and performed the experiments, analyzed the data, interpreted the results, and wrote the manuscript. M.K.-A. contributed to experimental design and manuscript writing. K.U. analyzed the scRNA-seq data. Y.Y. conducted the PCR experiments. M.S. quantified various hormone levels. S.L., A.R., and K.W. created the PB-TA-MCS (KW107) and PB-CAG-tTA-IREShyg (KW1526). K.C., H.M., and M.F. contributed to experimental design. T.A. oversaw the experiment design, result interpretation, and manuscript writing.

## Declaration of interests

We have patents related to this work.

Title: Method for preparing human pluripotent stem cell-derived Leydig-like cell, and human pluripotent stem cell-derived Leydig-like cell, patent number: 6979702(JP).

## Supplemental information

Document S1. Figures S1–S6

Table S1. Testosterone concentrations

Table S2. Genes Upregulated in LLCs

Table S3. Top 50 genes in Cluster 0, 4 and 9

Table S4. Subset1, Subset2 and LC

Table S5. Various hormone concentrations

Document S2. Article plus supplemental information
