# Supplementary material for: Efficient differentiation of human iPSCs into Leydig-like cells capable of long-term stable secretion of testosterone
Source: Stem Cell Reports. 2025 Jan 16;20(2):102392. doi: 10.1016/j.stemcr.2024.102392 (PMC11864132; doi:10.1016/j.stemcr.2024.102392)
Supplement: Document S1. Figures S1–S6 [file mmc1.pdf]

Stem Cell Reports, Volume *20*

Supplemental Information

Efficient differentiation of human iPSCs into Leydig-like cells capable of long-term stable secretion of testosterone

Katsuya Sato, Michiyo Koyanagi-Aoi, Keiichiro Uehara, Yosuke Yamashita, Masakazu Shinohara, Suji Lee, Anika Reinhardt, Knut Woltjen, Koji Chiba, Hideaki Miyake, Masato Fujisawa, and Takashi Aoi

Figure S1

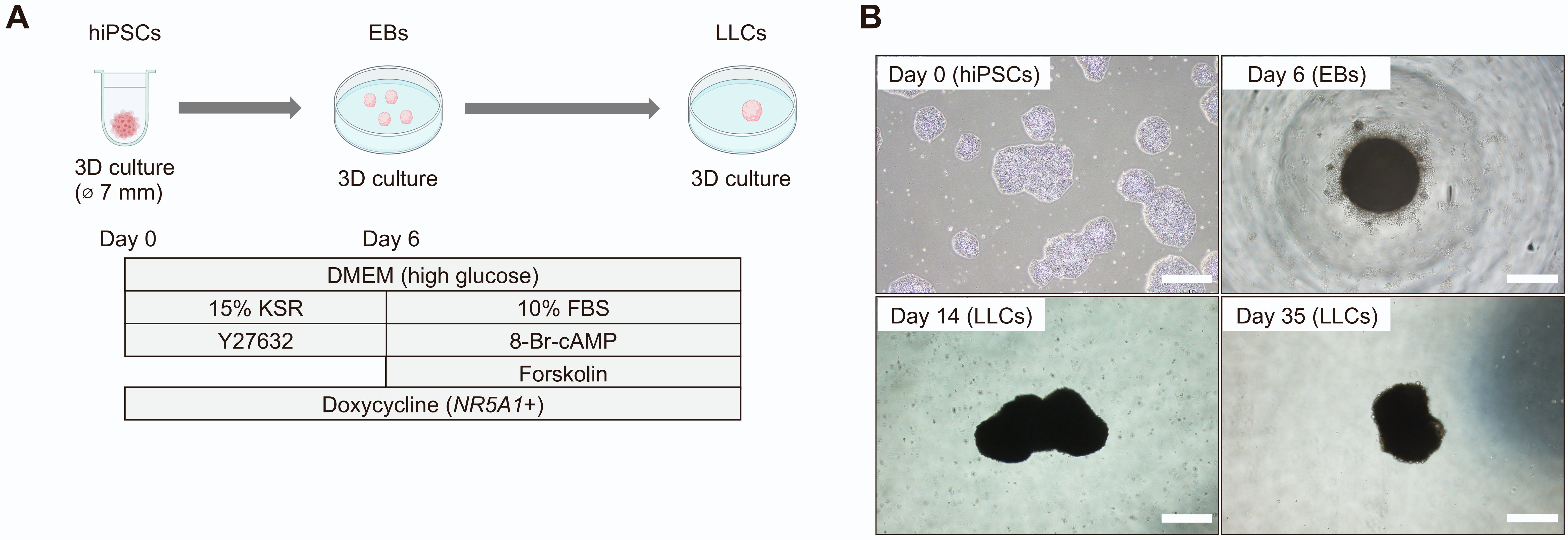
A
hiPSCs
EBs
LLCs
3D culture
(⌀ 7 mm)
3D culture
3D culture
Day 0
Day 6
DMEM (high glucose)
15% KSR
10% FBS
Y27632
8-Br-cAMP
Forskolin
Doxycycline (*NR5A1*+)
B
Day 0 (hiPSCs)
Day 6 (EBs)
Day 14 (LLCs)
Day 35 (LLCs)

Figure S2

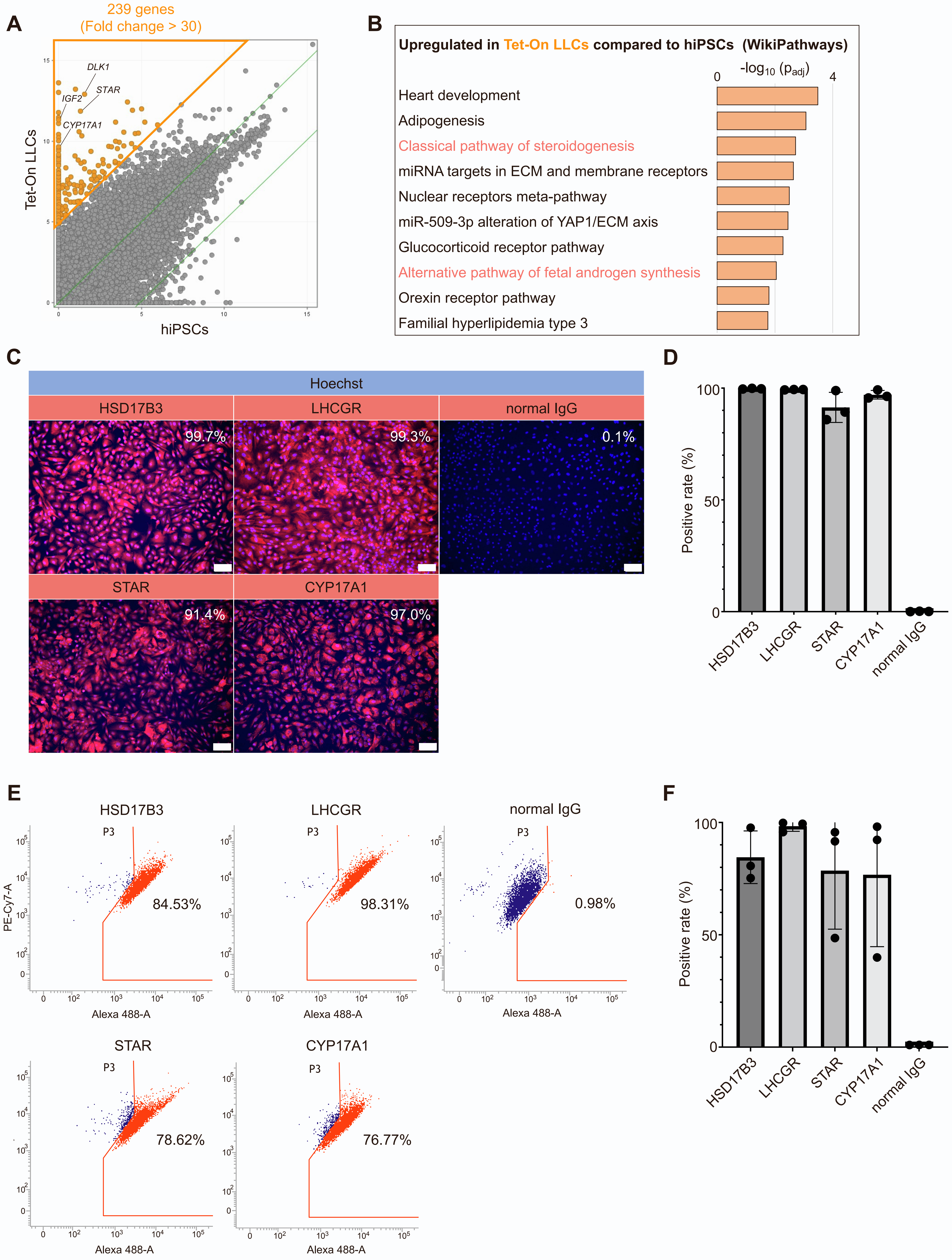
A
239 genes
(Fold change > 30)
DLK1
STAR
IGF2
CYP17A1
Tet-On LLCs
hiPSCs
B
Upregulated in Tet-On LLCs compared to hiPSCs (WikiPathways)
-log10 (padj)
Heart development
Adipogenesis
Classical pathway of steroidogenesis
miRNA targets in ECM and membrane receptors
Nuclear receptors meta-pathway
miR-509-3p alteration of YAP1/ECM axis
Glucocorticoid receptor pathway
Alternative pathway of fetal androgen synthesis
Orexin receptor pathway
Familial hyperlipidemia type 3
C
Hoechst
HSD17B3
LHCGR
normal IgG
99.7%
99.3%
0.1%
STAR
CYP17A1
91.4%
97.0%
D
Positive rate (%)
HSD17B3
LHCGR
STAR
CYP17A1
normal IgG
E
HSD17B3
LHCGR
normal IgG
P3
84.53%
98.31%
0.98%
PE-Cy7-A
Alexa 488-A
STAR
CYP17A1
78.62%
76.77%
F
Positive rate (%)

Figure S3

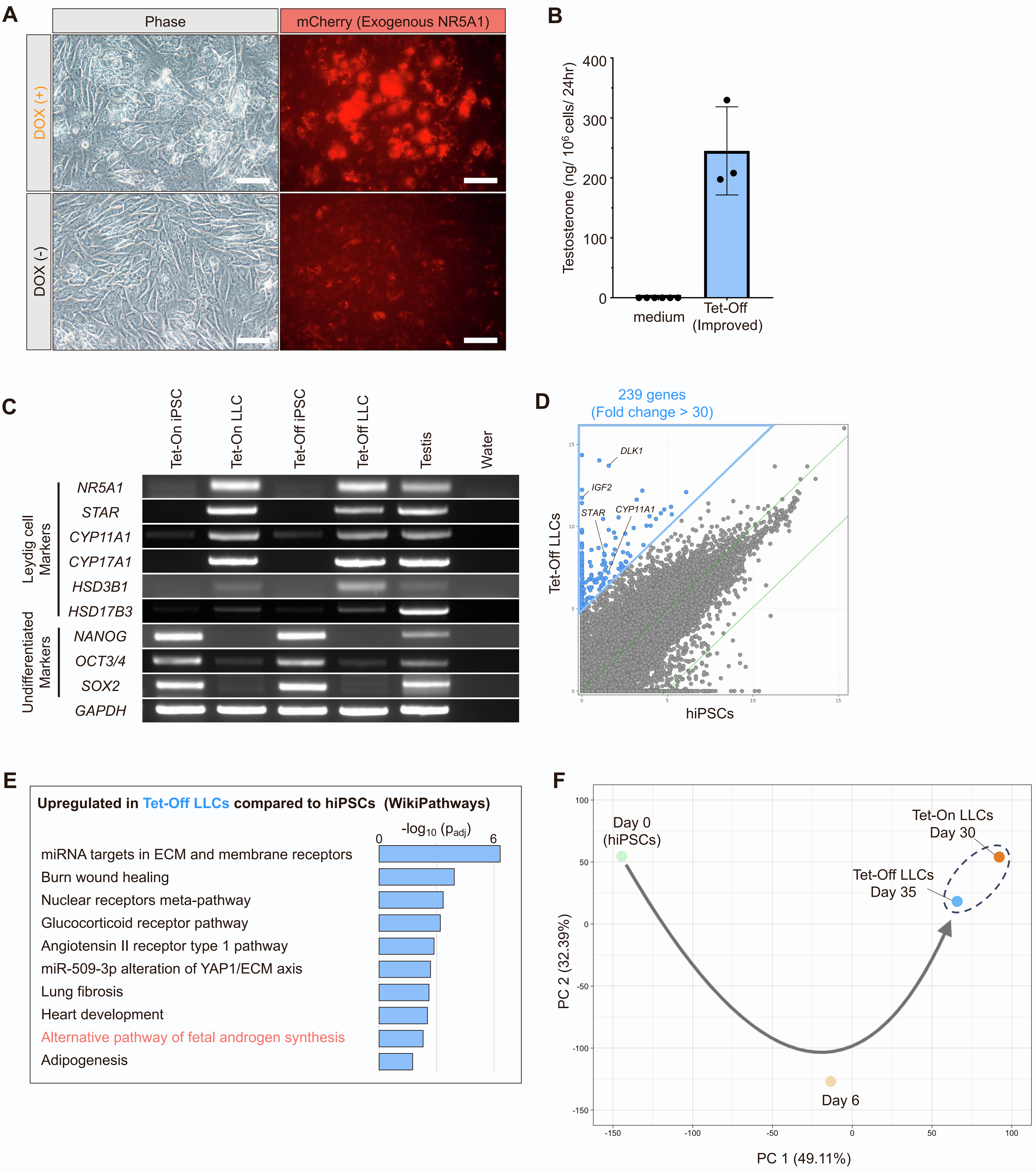

A
Phase
mCherry (Exogenous NR5A1)
DOX (+)
DOX (-)
B
Testosterone (ng/ 10^6 cells/ 24hr)
400
300
200
100
0
medium
Tet-Off (Improved)
C
Tet-On iPSC
Tet-On LLC
Tet-Off iPSC
Tet-Off LLC
Testis
Water
Leydig cell Markers
Undifferentiated Markers
NR5A1
STAR
CYP11A1
CYP17A1
HSD3B1
HSD17B3
NANOG
OCT3/4
SOX2
GAPDH
D
239 genes
(Fold change > 30)
DLK1
IGF2
STAR
CYP11A1
Tet-Off LLCs
hiPSCs
E
Upregulated in Tet-Off LLCs compared to hiPSCs (WikiPathways)
-log10 (padj)
0
6
miRNA targets in ECM and membrane receptors
Burn wound healing
Nuclear receptors meta-pathway
Glucocorticoid receptor pathway
Angiotensin II receptor type 1 pathway
miR-509-3p alteration of YAP1/ECM axis
Lung fibrosis
Heart development
Alternative pathway of fetal androgen synthesis
Adipogenesis
F
Day 0
(hiPSCs)
Tet-On LLCs
Day 30
Tet-Off LLCs
Day 35
Day 6
PC 2 (32.39%)
PC 1 (49.11%)
100
50
0
-50
-100
-150
-150
-100
-50
0
50
100

Figure S4

A

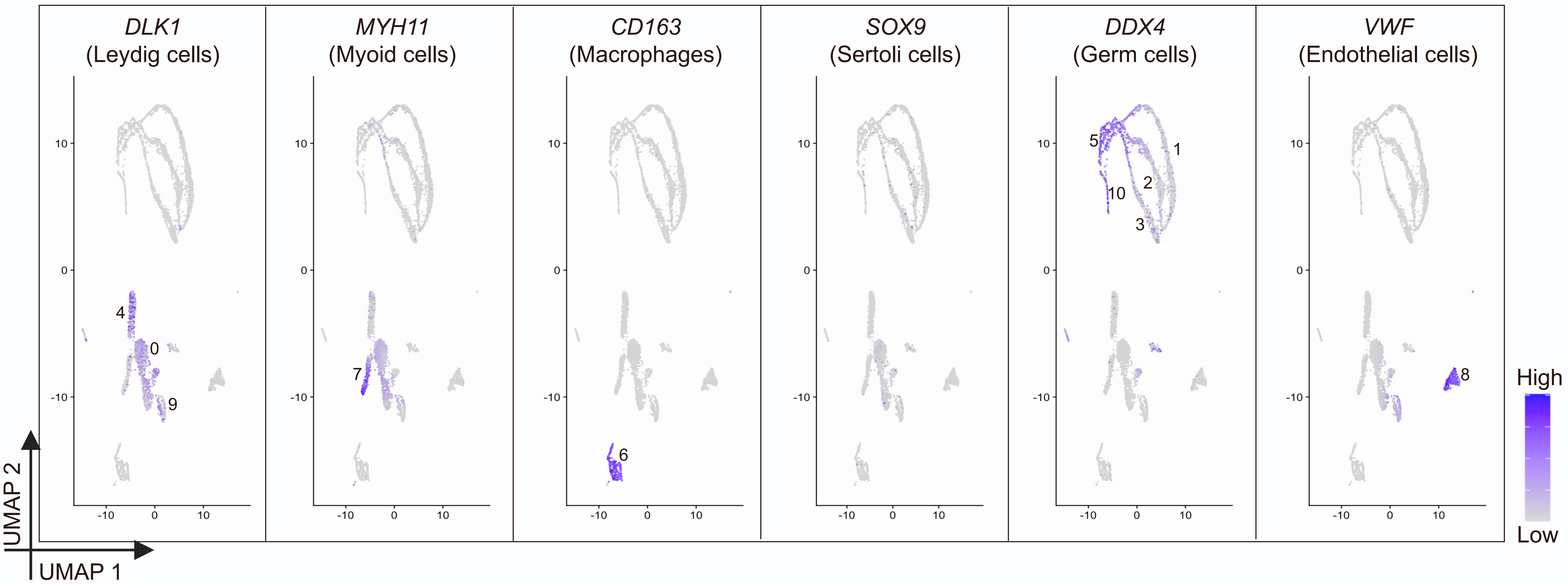

B

Subset 1 > Subset 2	Subset 2 > Subset 1
TEX30	*MMRN1*
PTRH1	*CXCL3*
NRK	*CCL21*
HSPA12A	*CXCL2*
PCDH18	*FLI1*
KANK2	*NES*
MMP23B	*PDLIM1*
MIR202HG	*WFDC2*
HSPA5	*LAPTM5*
CYP11A1	*SORBS2*

C

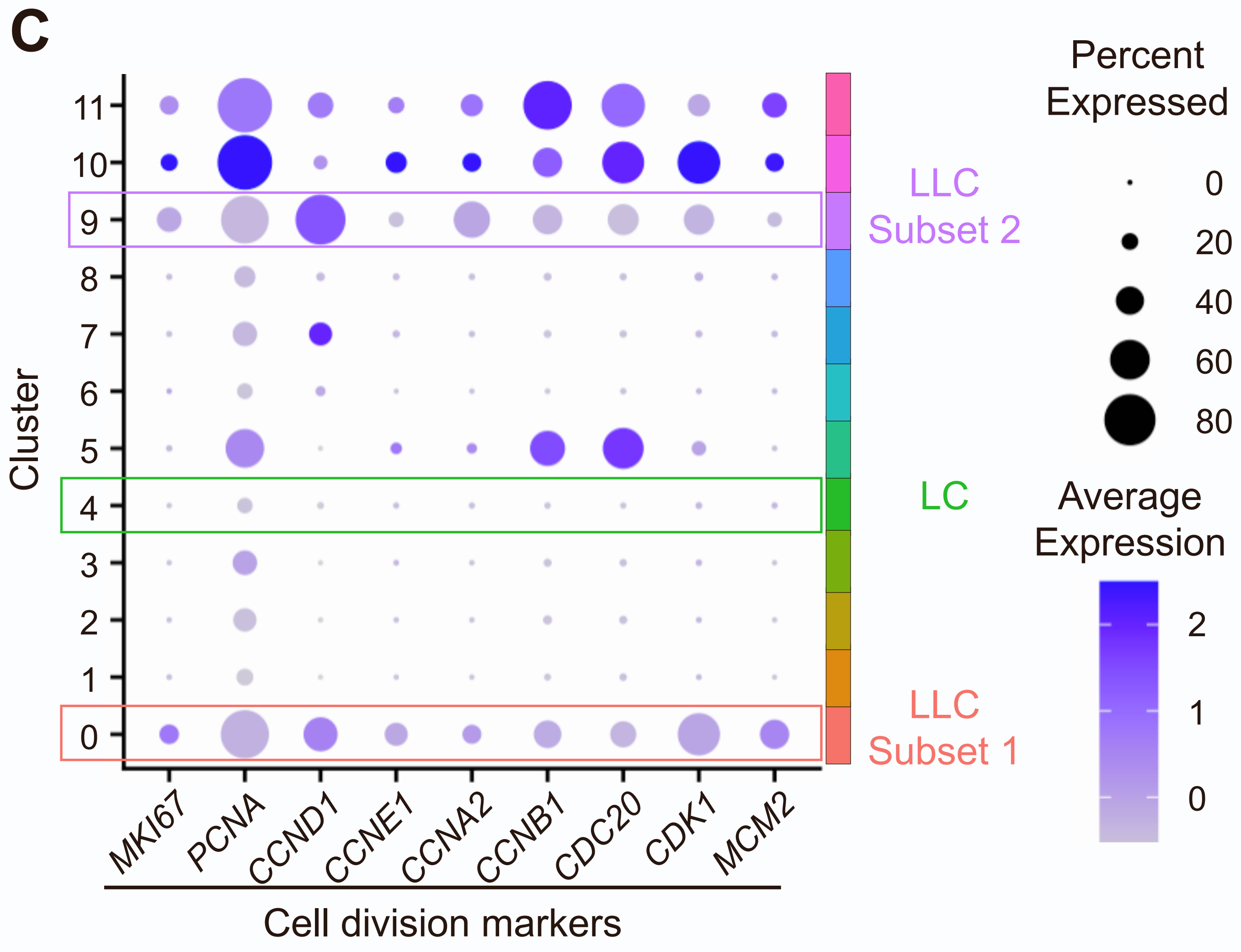

D

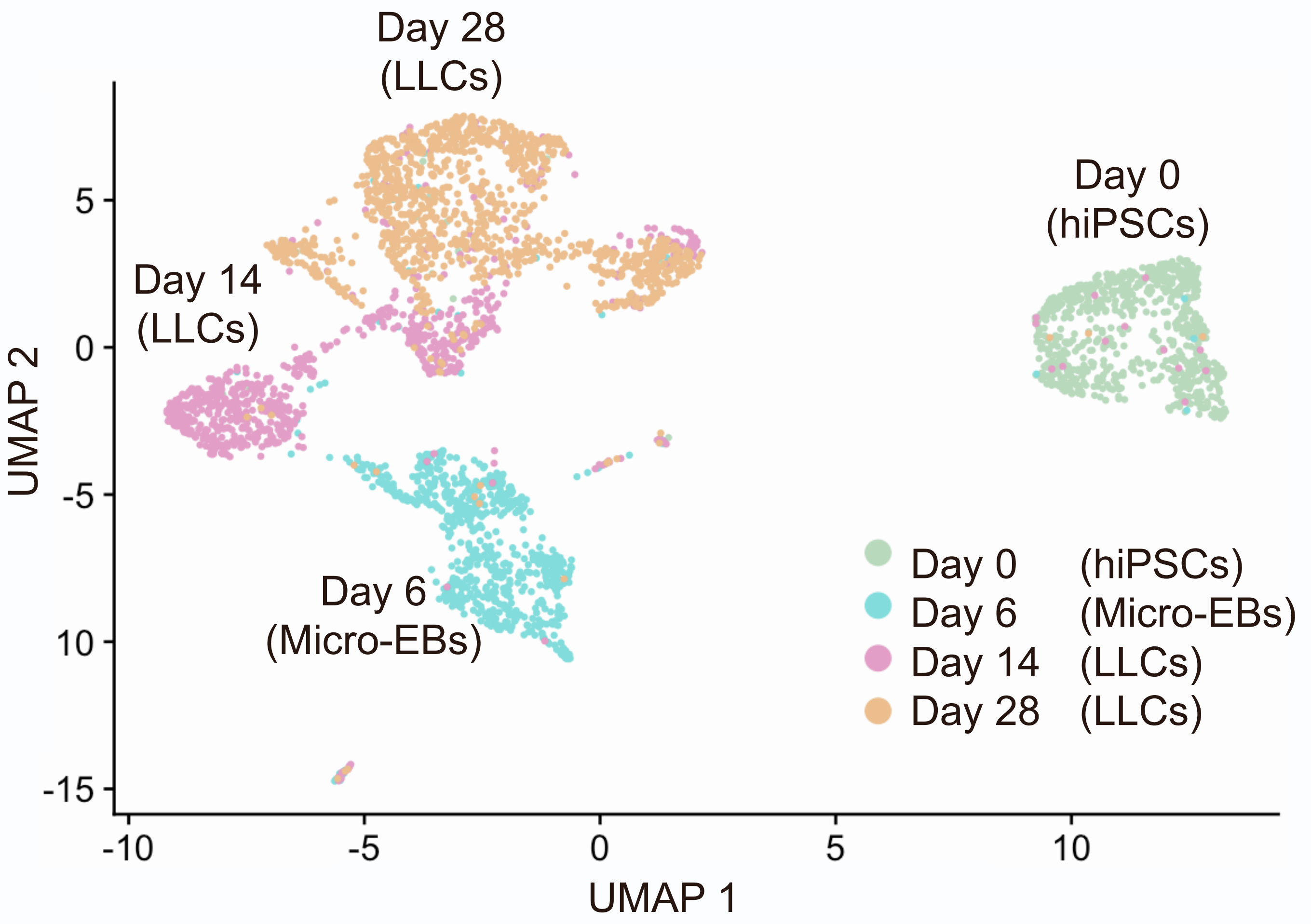

E

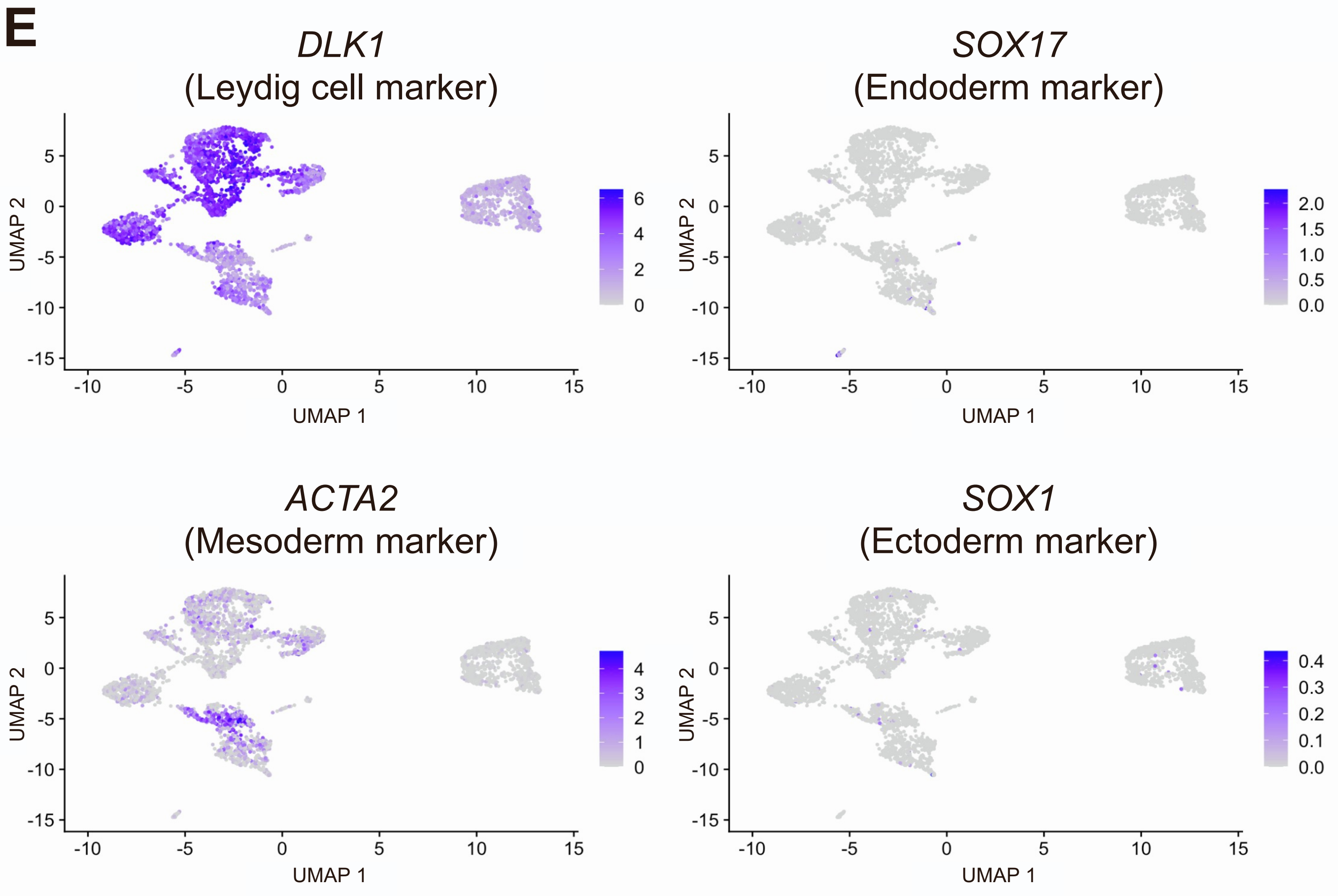

Figure S5

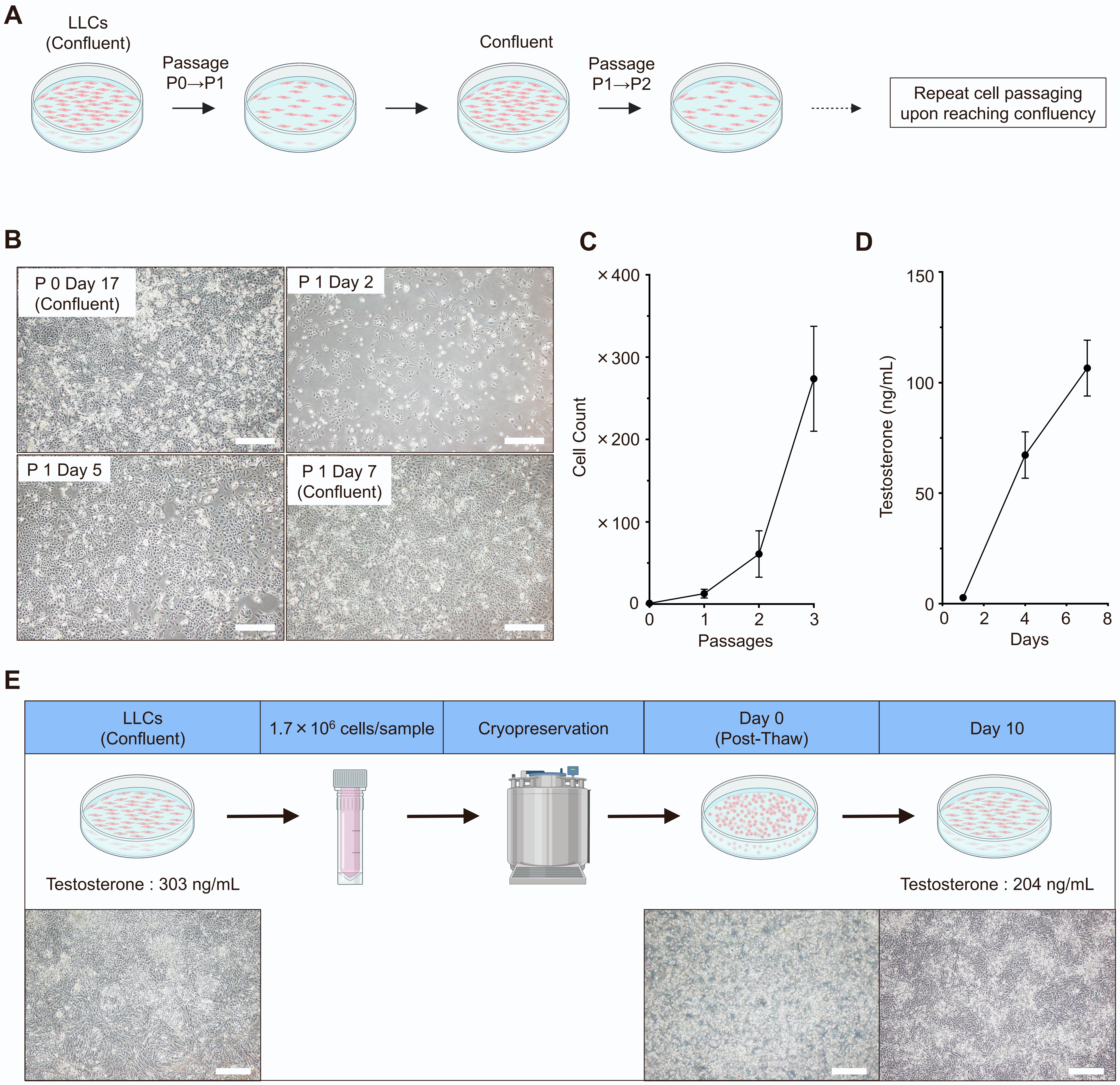

A
LLCs
(Confluent)
Passage
P0→P1
Confluent
Passage
P1→P2
Repeat cell passaging
upon reaching confluency
B
P 0 Day 17
(Confluent)
P 1 Day 2
P 1 Day 5
P 1 Day 7
(Confluent)
C
Cell Count
× 400
× 300
× 200
× 100
0
0
1
2
3
Passages
D
Testosterone (ng/mL)
150
100
50
0
0
2
4
6
8
Days
E
LLCs
(Confluent)
1.7 × 10^6 cells/sample
Cryopreservation
Day 0
(Post-Thaw)
Day 10
Testosterone : 303 ng/mL
Testosterone : 204 ng/mL

Figure S6

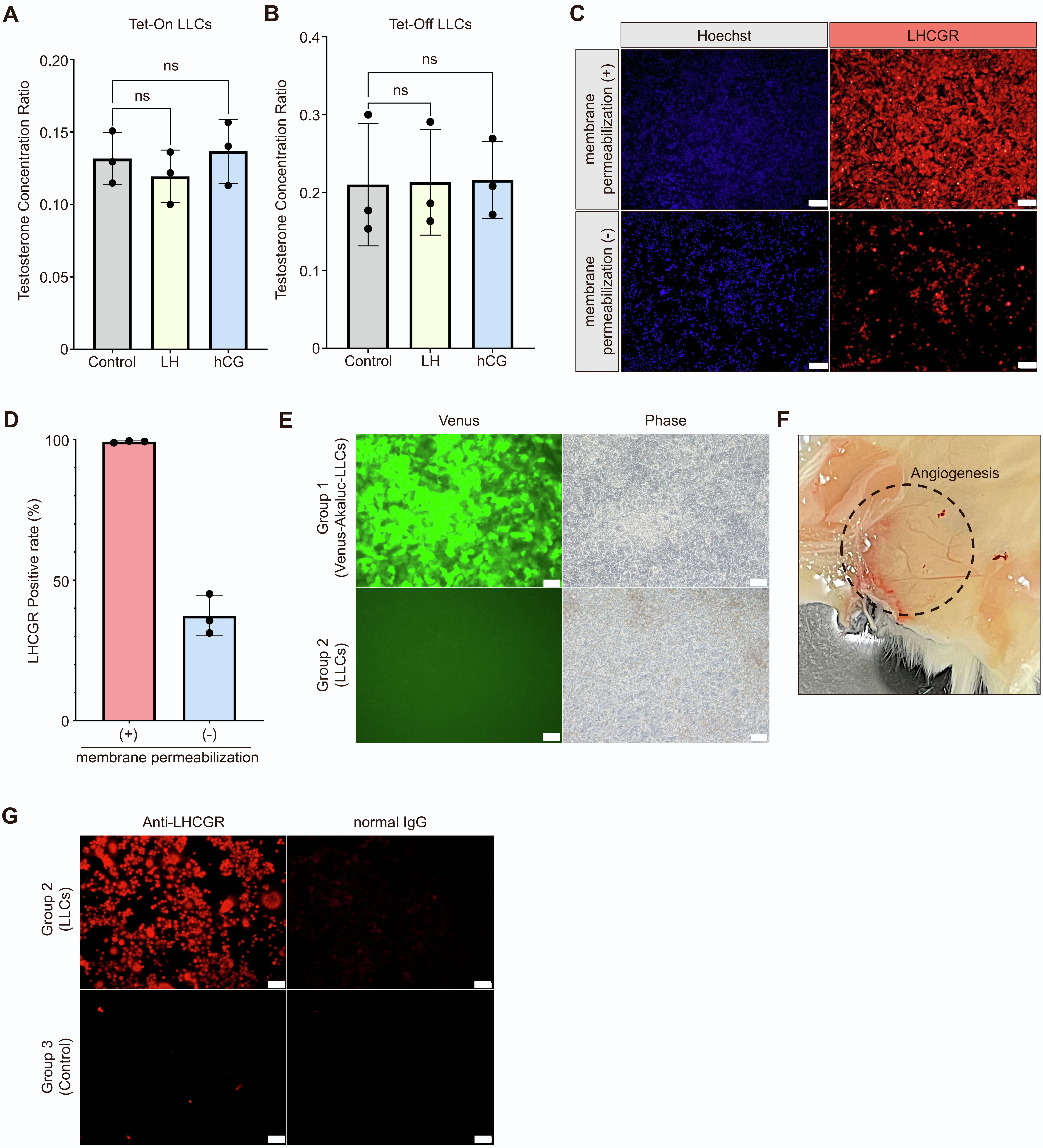
A
Tet-On LLCs
Testosterone Concentration Ratio
0.20
0.15
0.10
0.05
0
ns
ns
Control
LH
hCG
B
Tet-Off LLCs
Testosterone Concentration Ratio
0.4
0.3
0.2
0.1
0
ns
ns
Control
LH
hCG
C
Hoechst
LHCGR
membrane permeabilization (+)
membrane permeabilization (-)
D
LHCGR Positive rate (%)
100
50
0
(+)
(-)
membrane permeabilization
E
Venus
Phase
Group 1 (Venus-Akaluc-LLCs)
Group 2 (LLCs)
F
Angiogenesis
G
Anti-LHCGR
normal IgG
Group 2 (LLCs)
Group 3 (Control)

SUPPLEMENTARY FIGURE LEGENDS

Figure S1. Conventional method of generating hiPSC-derived LLCs

A) Schematic representation of the conventional differentiation protocol for generating LLCs from hiPSCs. This method involves the forced expression of *NR5A1* in hiPSCs, which are then maintained in 3D culture.

B) Morphological changes in cells generated by the conventional method, as observed by phase contrast microscopy on days 0 (hiPSCs), 6 (embryoid bodies), 14, and 35 (LLCs). Scale bars: 500 μm.

Figure S2. The evaluation of Tet-On LLCs using NGS, immunostaining, and flow cytometry

A) The gene expression of a comprehensive set of 53,827 genes was examined using next-generation sequencing (NGS). Among the examined genes, 239 showed a substantial 30-fold increase in expression on day 14 (LLCs) compared with day 0 (hiPSCs).

B) A pathway analysis was conducted on the 239 genes extracted using the methods described above, leveraging the WikiPathways database, and the top 10 pathways were subsequently identified.

C) Leydig cell marker levels were assessed using an immunofluorescence assay. The percentages presented in the figure are the average positivity rates from three independent experiments, which assessed LLCs at days 28-35 of culture. Scale bars: 100 μm.

D) Statistical analyses of the immunofluorescence assay. Positivity was quantitatively evaluated using the ImageJ software program. Mean value ± SD (n = 3 independent experiments).

E) Quantification of the Leydig cell markers was performed using flow cytometry. The LLCs were analyzed on days 27-48 of cell culture. A two-dimensional analysis was conducted to ascertain the positivity rate.

F) Statistical analyses of flow cytometry. Mean value ± SD (n = 3 independent experiments).

Figure S3. The evaluation of Tet-Off LLCs

A) The removal of doxycycline from Tet-On LLCs medium resulted in a decrease in the red fluorescent protein expression of mCherry. This suggested that the expression of exogenous *NR5A1* ceased. Scale bars: 50 μm.

B) Testosterone concentration in the culture supernatant of Tet-Off LLCs was measured. Cells were detached from the culture dish using 0.5X TrypLE Select, and the cell count was measured to calculate the amount of testosterone secreted per million cells in 24 h. The findings are expressed as the mean ± SD from independent experiments, with sample sizes of n = 6 for the control (medium) group, n = 3 for the Tet-Off LLCs group.

C) Semi-quantitative RT-PCR showed that Tet-On LLC and Tet-Off LLC cells express steroid hormone metabolism genes (*STAR*, *CYP11A1*, *CYP17A1*, *HSD3B1*, and *HSD17B3*). The testis was used as a positive control for the marker genes.

D) A comprehensive set of 53,827 genes was analyzed for gene expression using NGS. Among the examined genes, 239 showed a 30-fold increase in expression in Tet-Off LLCs on day 35 compared to hiPSCs.

E) A WikiPathway analysis was conducted on the 239 identified genes using the methods described above. The top 10 pathways were identified.

F) A principal component analysis of NGS data from day 0 (hiPSCs), Tet-On day 6 (micro-EBs), Tet-On LLCs (day 30), and Tet-Off LLCs (day 35). The horizontal axis represents the score of principal component 1, and the vertical axis represents the score of principal component 2.

Figure S4. Characterization of gene expression changes during the induction of LLC differentiation using scRNA-seq

A) Cluster identification was performed using markers specific to each cell type.

B) The top 10 genes upregulated in each subset are listed when comparing LLC subsets 1 and 2 (See also Table S5).

C) The expression of the genes associated with cell division was compared among the identified clusters and is represented using dotplots.

D) UMAP plots were generated using cell sample data collected at various time points during the Tet-On LLC differentiation induction process (days 0, 6, 14, and 28).

E) UMAP plots indicate changes in the expression of DLK1 (a Leydig cell marker), SOX17 (an endoderm marker), ACTA2 (a mesoderm marker), and SOX1 (an ectoderm marker) on days 0, 6, 14, and 28 after differentiation induction.

Figure S5. LLCs can be proliferated by passaging and can also be cryopreserved and thawed

A) Once the LLCs reached confluence, they were passaged at 1/6 cell density in new wells. This was repeated each time the cells reached confluence.

B) On day 17 of differentiation induction, LLCs that had reached confluence were passaged into another well, and images were captured using phase-contrast microscopy on days 2, 5, and 7 post-passaging. Scale bars: 500 µm.

C) Proliferation curves for repeated passages of LLCs. The proliferation rate was evaluated in triplicate, and a curve was plotted. The vertical axis represents the cell count, with the cell count on P0 day 17 set at 1. Mean ± SD, n = 3 independent experiments.

D) Changes in testosterone concentration in culture supernatant after the first passaging. The data represent the mean ± SD of three independent experiments, each performed in triplicate.

E) LLCs that reached confluence were pipetted into tubes at a density of 1.7×10^6 cells/vial, frozen in liquid nitrogen, thawed, and subsequently cultured again. After thawing, LLCs secreted large amounts of testosterone into the culture supernatant. LLCs were imaged using phase-contrast microscopy pre-cryopreservation, immediately post-thaw (day 0), and on day 10 post-thaw. Scale bars: 500 µm.

Figure S6. Response of LLCs to gonadotropic hormones and LLCs used for transplantation

A-B) The vertical axis represents the ratio of the testosterone concentration in the culture supernatant after 3 h of incubation with LH (5 ng/mL) or hCG (1 U/mL)

relative to the baseline testosterone concentration measured during the preceding 24 h (from -24 to 0 h) in Tet-On LLCs (A) and Tet-Off LLCs (B). The values represent the mean ± SD (n = 3 independent experiments).

C) To investigate the localization of LHCGR on the cell surface, we performed immunostaining for LHCGR in Tet-Off LLCs with and without membrane permeabilization (upper and lower panels, respectively). Scale bars: 100 µm.

D) The percentage of LHCGR-positive cells was quantitatively evaluated using ImageJ . The values represent the mean ± SD (n = 3 independent experiments).

E) LLCs transfected with Venus-Akaluc (Group 1) displayed green Venus fluorescence, even after being cultured on PET membranes. Scale bars: 50 µm.

F) The subcutaneous tissue from the mice showed the development of angiogenic vessels in the area, consistent with the implantation of PET membranes.

G) Immunostaining of PET membranes harvested from mice indicated the presence of cells expressing the Leydig cell-specific marker LHCGR on the membranes. Scale bars: 50 µm.

Video S1. Differentiation of mesodermal embryoid bodies into LLCs. Micro-EBs transitioned from a 3D culture to an adherent culture to induce differentiation into LLCs on day 6. Images were captured every 5 min using a time-lapse phase-contrast microscope. Scale bar: 200 µm.

KEY RESOURCES TABLE

Reagent or Resource	SOURCE	IDENTIFIER
**Antibodies**		
Rabbit anti-HSD17B3	Genetex	Cat# GTX114480
Rabbit anti-LHCGR	Genetex	Cat# GTX100008
Rabbit anti-StAR	Genetex	Cat# GTX105716
Rabbit anti-CYP17A1	Genetex	Cat# GTX56294
Normal Rabbit IgG	FUJIFILM Wako Pure Chemical Corporation	Cat# 148-09551
donkey anti-rabbit IgG, Alexa Fluor 488	Thermo Fisher Scientific	Cat# A-21206
donkey anti-rabbit IgG, Alexa Fluor 594	Thermo Fisher Scientific	Cat# A-21207
Hoechst 33342, trihydrochloride	Thermo Fisher Scientific	Cat# H3570
**Chemicals, peptides, and recombinant proteins**		
StemFit AK02N	Ajinomoto	Cat# AJ100
Penicillin-Streptomycin	Life Technologies	Cat# 15140-122
1X TrypLE Select	Thermo Fisher Scientific	Cat# A12859-01
0.5 mol/l-EDTA Solution	Nacalai Tesque	Cat# 06894-14
iMatrix-511 silk	Nippi	Cat# 892021
CultureSure Y-27632	FUJIFILM Wako Pure Chemical Corporation	Cat# 034-24023
DMEM High Glucose	Nacalai Tesque	Cat# 08459-64
KnockOut SR	Life Technologies	Cat# 10828-010
Doxycycline hyclate	Tocris Bioscience	Cat# 4090
Fetal Bovine Serum (FBS)	Biowest	Cat# S1650
8-Bromoadenosine-3',5'-cyclic Monophosphate Sodium Salt Hydrate	Nacalai Tesque	Cat# 05450-86
Forskolin	Abcam	Cat# ab120058
CHIR99021	TOCRIS	Cat# 252917-06-9
BMP4	R&D Systems	Cat# 314-BP-050
VEGF	R&D Systems	Cat# 293-VE-050
TRIzol	Life Technologies	Cat# 15596018
4% paraformaldehyde phosphate buffer solution	FUJIFILM Wako Pure Chemical Corporation	Cat# 163-20145

Blocking One	Nacalai Tesque	Cat# 03953-95
Tween 20	Nacalai Tesque	Cat# 28353-85
Triton X-100	Nacalai Tesque	Cat# 35501-15
Luteinizing Hormone from human pituitary	Sigma-Aldrich	Cat# L6420-10UG
Gonadotropin for Injection, 5000 Units	ASKA Pharmaceutical Co., Ltd.	Cat# 123-00080-2
LR Clonase™ II Plus	ThermoFisher Scientific	Cat# 12538200
OPTI-MEM (1X)	Thermo Fisher Scientific	Cat# 31985-062
FuGENE HD	Roche	Cat# E2311
Hygromycin B	FUJIFILM Wako Pure Chemical Corporation	Cat# 084-07681
Trypan Blue Stain (0.4%)	Life Technologies	Cat# 15250-061
STEM-CELLBANKER	ZENOAQ	Cat# CB047
AkaLumine-HCl	FUJIFILM Wako Pure Chemical Corporation	Cat# 018-26703
**Critical commercial assays**		
TURBO DNA-free kit	Life Technologies	Cat# AM1907
SMARTer stranded total RNA-seq kit	Takara Bio	Cat# 634836
One-Step RT-ddPCR Advanced Kit for Probes	Bio-Rad	Cat# 1864021
PrimeScript II 1st Strand Synthesis Kit	Takara	Cat# 6210A
**Experimental models: Organisms/strains**		
PB-TAC-ERN-NR5A1	Ishida et al., 2021[16]	N/A
PB-TA-MCS	Knut Woltjen	N/A
PB-CAG-tTA-IRESHyg	Knut Woltjen	N/A
pcDNA3 Venus-Akaluc	RIKEN	Cat# RDB15781
pMXs retrovirus vector (pMXs-hOCT3/4)	addgene	Cat #17217
NSG mice (NOD.Cg-Prkdcscid Il2rgtm1Wjl/SzJ)	The Jackson Laboratory Japan	N/A
Total RNA - Human Adult Normal Tissue: Testis	BioChain	Cat# R1234260-50
**Deposited data**		
NGS data	This paper	GEO: GSE244796

Single cell RNA-seq for adult human testes	Guo et al., 2018	GEO: GSE112013
Single cell RNA-seq for Leydig-like cells	This paper	GEO: GSE245553
**Software and algorithms**		
Strand NGS software	Strand Life Sciences	N/A
R	N/A	v4.1.2
g:Profiler	Uku Raudvere et al., 2019[50]	https://biit.cs.ut.ee/gprofiler/gost
Seurat	Butler et al., 2019[51]	N/A
ImageJ Fiji software	National Institutes of Health	https://imagej.net/Fiji
QuantaSoft software	Bio-Rad	N/A
Living Image software	PerkinElmer	v4.3
The Human Protein Atlas website	N/A	https://www.proteinatlas.org/
**Culture Dish**		
PrimeSurface96M	Sumitomo Bakelite	Cat# MS-9096M
PrimeSurface24F	Sumitomo Bakelite	Cat# MS-90240
AggreWell400 (6-well plate)	STEMCELL Technologies	Cat# 34421
Anti-Adherence Rinsing Solution	STEMCELL Technologies	Cat# 07010
Nunc Cell-Culture Treated Multidish (6-well plate)	Life Technologies	Cat# 140675
Nunc Cell-Culture Treated Multidish (24-well plate)	Life Technologies	Cat# 142475
0.4-μm pore size PET-Track-attached-membrane-12-well format	Corning	Cat# 353180
12-well TC-treated Polystyrene Permeable Support Companion Plate	Corning	Cat# 353503
**Other**		
BioStudio	Nikon	N/A
inverted fluorescence microscope	Olympus Corporation	N/A
BD Rhapsody Express Single-Cell Analysis System	BD Biosciences	N/A
FACSVerse instrument	BD Biosciences	N/A
QX200 Droplet Generator	Bio-Rad	N/A
C1000 Touch Thermal Cycler	Bio-Rad	N/A

QX200 Droplet Reader	Bio-Rad	N/A
IVIS Lumina LT imaging system	PerkinElmer	N/A
Countess3 Automated Cell Counter	Invitrogen	Cat# AMQAX2000

SUPPLEMENTAL EXPERIMENTAL PROCEDURE

hiPSC culture

In this study, two types of hiPSCs (3AB4_NR5A1_hiPSC (Ishida et al., 2021) and 121-3 (Murai et al., 2023)) were used. This study was approved by the Ethics Committee of Kobe University Graduate School of Medicine (No. 1722), and informed consent was obtained from all donors. 3AB4 iPSC line and LLCs generated from this line were used in the experiments in Figures 1B-G, 2A, 2E-H, 3B-F, 4G-H, S1B, S2A-F, S3A, S3C, S4A-E and Video S1. On the other hand, 121-3 iPSC line and LLCs generated from this line were used in Figures 2D-H, 3B, 4A-E, 4G-H, 5B-H, 6A-I, S3B-F, S5B-E and S6A-G.

The hiPSCs were cultured using a previously described method (Nakagawa et al., 2014) with some modifications. In brief, hiPSCs were cultured in StemFit AK02N (Ajinomoto, Tokyo, Japan) supplemented with penicillin (50 units/mL) and streptomycin (50 µg/mL) (Life Technologies, Carlsbad, CA, USA) and maintained at 37°C, 5% CO_2. The culture medium was changed every other day and passaged with 0.5X TrypLE Select (1X TrypLE Select [Thermo Fisher Scientific, Waltham, MA, USA] diluted 1:1 with 0.5 mM EDTA Solution [Nacalai Tesque, Kyoto, Japan]/phosphate-buffered saline [PBS]) every 7 days. For cell attachment, culture dishes were precoated with iMatrix-511 silk (Nippi, Tokyo, Japan) at 0.5 µg/cm^2, and CultureSure Y-27632 (10 µM; FUJIFILM Wako Pure Chemical Corporation, Osaka, Japan) was added to the medium for 1 day to promote the cell survival and attachment.

Conventional method for induction of differentiation into LLCs

The conventional method for inducing differentiation of hiPSCs into LLCs was

performed with some modifications to the previous literature (Ishida *et al.*, 2021). In brief, for EB formation, 3×10^4 3AB4-NR5A1-hiPSCs were allowed to aggregate in 100 µL of maintenance medium (DMEM High Glucose [Nacalai Tesque] with 15% KnockOut SR [Life Technologies], CultureSure Y-27632 [10 µM], penicillin [50 units/mL], streptomycin [50 µg/mL], and doxycycline hyclate [1.5 µM; Tocris Bioscience, Bristol, UK]) and seeded into each well of non-adherent 96-well culture dishes (**PrimeSurface96M**; Sumitomo Bakelite, Tokyo, Japan) for incubation in a 5% CO_2 environment at 37°C for 6 days without replenishing the medium. Subsequently, four EBs were transferred to each well of 24-well culture dishes (**PrimeSurface24F**; Sumitomo Bakelite) containing 500 µL/well differentiation medium (DMEM High Glucose with 10% Fetal Bovine Serum [FBS] [Biowest, Nuaille, France], 8-Bromoadenosine-3',5'-cyclic Monophosphate Sodium Salt Hydrate [8Br-cAMP] [1 mM, Nacalai Tesque], forskolin [100 µM, Abcam, Cambridge, UK], penicillin [50 units/mL], streptomycin [50 µg/mL], and doxycycline hyclate [1.5 µM]). All media were refreshed every two to three days starting on day 6.

Sequential imaging of cellular morphological changes

On day 6, micro-EBs were transferred to adherent culture dishes. A phase-contrast microscope (BioStudio; Nikon, Tokyo, Japan) was used to determine the time-lapse function. High-resolution images were acquired every 5 min for 48 h.

Measurement of testosterone concentrations in culture supernatants

Culture supernatants were collected every three days, and testosterone concentrations were quantitatively measured using an electrochemiluminescence immunoassay

(ECLIA) performed by SRL (Tokyo, Japan).

RT-PCR analyses

Total RNA was extracted using TRIzol reagent (Life Technologies), and genomic DNA contamination was eliminated using a TURBO DNA-free kit (Life Technologies). A total of 650 ng of total RNA was reverse-transcribed into cDNA using the PrimeScript II 1st Strand Synthesis Kit (Takara, Shiga, Japan) in accordance with the manufacturer's instructions. Water served as the negative control, whereas total testicular RNA (BioChain, Newark, CA, USA) served as the positive control. The primer sequences used for RT-PCR are outlined below.

**Gene name**		**primer (5'-3' orientation)**
GAPDH	forward	accacagtccatgccatcac
	reverse	tccaccaccctgttgctgta
OCT3/4	forward	gacaggggggagggggaggagctagg
	reverse	cttccctccaaccagttgccccaaac
NANOG	forward	tgaacctcagctacaaacag
	reverse	tggtggtaggaagagtaaag
SOX2	forward	gggaaatgggaggggtgcaaaagagg
	reverse	ttgcgtgagtgtggatgggattggtg
NR5A1	forward	aggagtacctgtaccacaagc
	reverse	tgcagcatttcgatgagcag
STAR	forward	ggttctcggctggaagagac
	reverse	gggacaggacctggttgatg
CYP11A1	forward	tgggtcgcctatcaccagta
	reverse	gttggccttgatgtcctcga
CYP17A1	forward	tggctctcttgctgcttacc
	reverse	gccacgaagacaggaaagga
HSD3B1	forward	cacatggcccgctccatac
	reverse	gtgccgccgtttttcagattc
HSD17B3	forward	gtcaacaatgtcggaatgcttc
	reverse	tgatgttacaatggatgaggctc

RNA sequencing

At various stages of differentiation induction, total RNA was extracted from the cells using TRIzol (Life Technologies) and subsequently treated with the TURBO DNA-free kit (Life Technologies). The treated RNA was then sent to Macrogen (Seoul, South Korea; https://www.macrogen.com) for library preparation using the SMARTer stranded total RNA-seq kit (Takara Bio) according to the manufacturer's instructions. Paired-end RNA sequencing datasets were generated using an Illumina NovaSeq6000 (Illumina, San Diego, CA, USA). Using the Strand NGS software program (Strand Life Sciences, Bangalore, India), the reads were aligned to the human transcriptome reference sequence (hg38). Heat maps were created by converting transcripts per million (TPM) values to z-scores, and a PCA was performed on all differentiated cells using the R software program (Version 4.1.2 (2021-11-01), R Foundation for Statistical Computing, Vienna, Austria). Using the g:Profiler software program, we conducted pathway analyses.

Immunofluorescence assays

Differentiated LLCs cultured in Nunc Cell-Culture-Treated Multidish (24-well plate; Life Technologies) were first fixed using 4% paraformaldehyde Phosphate Buffer Solution (FUJIFILM Wako Pure Chemical Corporation) for 15 min, followed by washing the cells with PBS three times. The cells were then treated with 0.5% Tween 20 (Nacalai Tesque) in 5% Blocking One (Nacalai Tesque) in PBS for 10 min at room temperature. Afterwards, the cells were incubated with primary antibodies overnight at 4°C. The primary antibodies used were anti-HSD17B3 (rabbit polyclonal antibody, dilution 1:100; Genetex, Irvine, CA, USA; #GTX114480), anti-LHCGR (rabbit polyclonal antibody, dilution 1:100; Genetex, #GTX100008), anti-StAR (rabbit polyclonal antibody, dilution 1:100; Genetex, #GTX105716), and anti-CYP17A1 (rabbit polyclonal antibody, dilution 1:50; Genetex,

#GTX56294). Normal rabbit IgG (4 μg/mL; FUJIFILM Wako Pure Chemical Corporation; #148-09551) was used as the isotype control. The following day, the cells were washed thrice with PBS. They were then incubated with secondary antibody (donkey anti-rabbit IgG, Alexa Fluor 594, dilution 1:500; Thermo Fisher Scientific) for 60 min at room temperature. Nuclear staining was performed using Hoechst 33342 (dilution, 1:10,000; Thermo Fisher Scientific). After the final PBS wash, cells were observed using an Olympus IX71 inverted microscope with an Olympus DP73 camera (Olympus Corporation, Tokyo, Japan). Quantitative measurements were performed using the ImageJ Fiji software program (National Institutes of Health, Bethesda, MD, USA) (Schindelin et al., 2012). In immunostaining of LHCGR (Figure S6C), cells without membrane permeabilization were not treated with 0.5% Tween 20.

Flow cytometry

Flow cytometry was also used to evaluate the differentiation efficiency. Specifically, the cell samples were fixed with 4% paraformaldehyde phosphate buffer solution for 15 min at room temperature and subsequently permeabilized with 0.3% Triton X-100 (Nacalai Tesque)/PBS for 30 min at room temperature. The cells were then incubated with primary antibodies or isotype control for 30 min at 4°C, followed by labeling with secondary antibodies for an additional 30 min at 4°C. The primary antibodies and isotype control were the same as those used in the immunofluorescence assay described above, with only the following dilutions adjusted: anti-HSD17B3 (dilution 1:20), anti-LHCGR (dilution 1:20), anti-StAR (dilution 1:20), and anti-CYP17A1 (dilution 1:10). Normal rabbit IgG (20 μg/mL) was used as the isotype control. The secondary antibody used was donkey anti-rabbit IgG Alexa Fluor 488 (dilution 1:25; Thermo Fisher Scientific). The labeled

samples were analyzed using a FACSVerse instrument (BD Biosciences, San Jose, CA, USA).

One-step digital droplet PCR

As previously reported (Kuroda et al., 2015), a one-step digital droplet PCR assay was performed to analyze the expression of *LIN28*, an undifferentiated marker. Total RNA was prepared as previously described. PCR mixtures (20 μL) were prepared using the One-Step RT-ddPCR Advanced Kit for Probes (Bio-Rad, Hercules, CA, USA). The mixture consisted of 5 μL of Supermix, 2 μL of Reverse Transcriptase, 1 μL of 300 mM DTT, 900 nM forward and reverse primers, 250 nM probe, and total RNA sample (100 ng). The primers and probe used for the analysis of the LIN28 gene in this study were designed based on the work of Kuroda et al. Specifically, the forward primer sequence for LIN28 is "CACGGTGCGGGCATCTG", and the reverse primer sequence is "CCTTCCATGTGCAGCTTACTC". Additionally, the probe sequence for LIN28 is "CGCATGGGGTTCGGCTTCCTGTCC". Droplets were generated using a QX200 Droplet Generator (Bio-Rad). RT-PCR was performed on a C1000 Touch Thermal Cycler (Bio-Rad). The thermal cycling conditions were as follows: reverse transcription at 50°C for 60 min, enzyme activation at 95°C for 10 min, and 40 cycles of a thermal profile comprising denaturation at 95°C for 30 s, and annealing/extension at 60°C for 1 min. After PCR amplification, products were denatured at 98°C for 10 min and cooled to 4°C. The fluorescence intensity of each droplet from the samples was measured using a QX200 Droplet Reader (Bio-Rad). Positive droplets containing amplification products were distinguished from negative droplets and counted by applying a fluorescence amplitude threshold in the QuantaSoft software program (Bio-Rad). The threshold was manually

determined at the highest point in the fibroblast sample droplet group. To calculate the positivity rate, the number of positive droplets was divided by the total number of droplets.

Generation of hiPSC lines with the forced expression of NR5A1 using the Tet-Off system

NR5A1 cDNA was introduced into the PB-TA-MCS (KW107) transposon vector using Gateway Cloning (Thermo Fisher Scientific) to create PB-TA-NR5A1. PB-CAG-tTA-IREShyg (KW1526) expresses Tet transactivator (tTA) and a hygromycin resistance gene and was constructed using the MultiSite Gateway Cloning technique with LR Clonase^{TM} II Plus (Thermo Fisher Scientific). These two transposon vectors were then co-delivered into hiPSCs (121-3) along with pCAG-PBase (KW158), which expresses the piggyBac transposase, using FuGENE HD (Roche, Basel, Switzerland), following the manufacturer's instructions. hiPSCs bearing transposons were enriched after 50 µg/ml of Hygromycin B (FUJIFILM Wako Pure Chemical Corporation) was added to the medium for 24 h. hiPSCs with the forced expression of *NR5A1* using the Tet-Off system were generated as described and maintained under the same culture conditions as the original hiPSCs, with the addition of 1 µM doxycycline to the maintenance medium.

Transmission Electron Microscope (TEM)

The cell samples for TEM were fixed in phosphate-buffered 2% glutaraldehyde (Electron Microscopy Science) and subsequently post-fixed in 2% osmium tetroxide (Heraeus Chemicals, South Africa) for 2 h in an ice bath. The specimens were then dehydrated in a graded ethanol series and embedded in an epoxy resin (TAAB Laboratories). Ultrathin sections were obtained using an ultramicrotome. Ultrathin sections stained with uranyl

acetate for 15 min and lead staining solution for 2 min were subjected to TEM observation at 100 kV (JEM-1400Flash, JEOL).

scRNA-seq analyses

We performed scRNA-seq using the BD Rhapsody Express Single-Cell Analysis System (BD Biosciences) and analyzed the data using the Seurat software program (http://satijalab.org/seurat/, R package version 4.1.2).

First, the LLCs were dissociated by adding 0.5X TrypLE Select to the culture dishes and incubating at 37°C for 2 min. Following the workflow manual, the cells were suspended in 1X PBS and loaded into cartridges along with the beads. Reverse transcription and PCR were performed. cDNA amplification, sequencing library preparation and a sequencing analysis using the TAS-Seq approach was performed by ImmunoGeneTeqs, Inc (Chiba, Japan). Next, we loaded scRNA-seq data from the publicly available dataset of testes from 3 adult males (17, 24, and 25 years old; GSE112013) (Guo et al., 2018) and our generated LLCs dataset. The cells were filtered to retain only those with high-quality expression profiles, setting min_cells = 3 and min_features = 500. For the adult testis dataset, we retained cells with 500 < nFeature_RNA < 5000 and percentage _mt <10. For the LLC dataset, we retained cells with 5000 < nFeature_RNA < 10000 and percent_mt <20. Next, we normalized the data using the LogNormalize method and identified the top 2,000 variable features for each dataset using the variance-stabilizing transformation (VST) method. We then performed unsupervised clustering using the top 10 principal components (PCs) and a resolution of 0.15 for both datasets. For data integration, we merged the two datasets into one Seurat object and prepared it for further analyses. The resulting cell clusters were visualized

using UMAP. In the UMAP plot, we identified distinct cell populations using specific marker genes: DLK1 for Leydig cells, MYH11 for myoid cells, CD163 for macrophages, SOX9 for Sertoli cells, DDX4 for germ cells, and VWF for vascular endothelial cells. This allowed us to compare the generated LLCs with normal testis cells and to understand their transcriptional landscape. Their expression patterns were visualized using feature plots, violin plots, dot plots, and heat maps. The top 50 genes characteristic of LC, LLC subset 1, and LLC subset 2 are also listed. From each set, five genes that were relevant to Leydig cells were identified through a PubMed search, now listed on the right side of the heatmap (Figure 3E). Cells at days 0, 6, and 28 of differentiation induction were also subjected to an scRNA-seq analysis in the same manner, and alterations in gene expression with the progression of differentiation induction days were represented in UMAP plots.

Passaging, cryopreservation, and thawing of LLCs

When LLCs reached confluence in 6-well plates, 0.5X TrypLE Select was added, and cells were dissociated by incubation at 37°C for 2 min. The cells were harvested and resuspended in Step 2 medium without cAMP. One well of cells was distributed into six wells for passaging, and once confluence was reached, passaging was repeated in the same manner. Cells were stained with Trypan Blue Stain (Life Technologies) and counted using a Countess3 Automated Cell Counter (Invitrogen). To determine whether or not LLCs could be cryopreserved and thawed, LLCs on day 17 of differentiation induction were dissociated with 0.5X TrypLE Select as described above and suspended in STEM-CELLBANKER (ZENOAQ, Fukushima, Japan) at a concentration of 1.6×10^{6} cells/500 μL. Cell suspensions were dispensed in 500 μL aliquots into cryopreservation vials. After

freezing the vials at -80°C for 24 h, the frozen vials were transferred to a liquid nitrogen storage tank. When thawing cells, vials were warmed in a 37°C water bath, and LLCs were resuspended in cAMP-free Step 2 medium and cultured in 1 well of a 6-well plate, with the medium changed every 2 days.

Response of LLCs to gonadotropic hormones

We investigated whether LLCs were capable of secreting testosterone in response to gonadotropic hormonal stimuli. To achieve this, we first measured the baseline testosterone concentration secreted by LLCs over a 24-hour period (-24 to 0 h). At 0 h, 5 ng/mL LH (Sigma Aldrich, St. Louis, MO, USA) or 1 U/mL hCG (gonadotropin; ASKA Pharmaceutical Co., Ltd., Tokyo, Japan) was added to the culture medium. The testosterone concentration in the culture supernatant was measured 3 h after addition.

Transfection of Venus-Akaluc

The target gene from pcDNA3 Venus-Akaluc (RIKEN, Japan) was integrated into the pMXs retrovirus vector using an infusion reaction, resulting in the generation of pMXs-Venus-Akaluc. The pcDNA3 Venus-Akaluc was provided by the RIKEN BRC through the National BioResource Project of the MEXT, Japan (cat. RDB15781). The pMXs-Venus-Akaluc plasmid vector was transfected into packaging cells (PLAT-A) using FuGENE HD (Roche) according to the manufacturer's instructions. The culture medium was changed on the day following transfection, and on the second day, the culture supernatant was collected and passed through a 0.45-μm filter. The filtered supernatant was supplemented with 8 μg/mL polybrene. LLCs used for infection were those on the second day (P1Day2) of culture following a single passaging event. Following

transfection, Venus-Akaluc-LLCs were cultured in the Step 2 medium without 8Br-cAMP. On day 21 post-retroviral infection, the expression of green Venus fluorescent proteins was visualized using a fluorescence microscope. The substrate AkaLumine-HCl (FUJIFILM Wako Pure Chemical Corporation) was added to 24-well plates at a concentration of 125 μg/well, and the luminescence of AkaBLI was observed using an IVIS Lumina LT imaging system (PerkinElmer, Waltham, MA, USA). The following parameters were used to capture images: exposure time of 60 s, medium binning of 8, XFOV field of view measuring 24 × 24 cm, and f/stop value of 1. The bioluminescence images produced by the IVIS system were analyzed using the specialized Living Image software program (PerkinElmer).

Adherent culture of LLCs on PET membranes

On day 21 of differentiation induction, LLCs cultured in two wells of the six-well plates were incubated with 0.5X TrypLE Select for 2 min to dissociate from the culture dish. The LLCs were resuspended in 2 mL of Step 2 medium and loaded into one well of a 0.4-μm pore size PET-Track-attached-membrane-12-well format (Corning, Corning, NY, USA; catalog #353180). Subsequently, this membrane was placed in 1 well of a 12-well TC-treated Polystyrene Permeable support anion plate (Corning; catalog #353503) containing 2 mL of Step 2 medium per well and incubated at 37°C, 5% CO_2.

Procurement and management of immunocompromised mice

All experimental protocols involving animals were approved by the Animal Experimentation Committee of Kobe University. The approval number for this study is P230206.

Four-week-old female NSG mice (NOD.Cg-Prkdcscid Il2rgtm1Wjl/SzJ; Jackson Laboratory, Kanagawa, Japan) were obtained and maintained in a sterile environment with free access to food and water. A minimum of five days was allowed for acclimation. Mice were divided into four groups: Group 1 received PET membranes with Venus-Akaluc-transfected LLCs, Group 2 received PET membranes with LLCs without Venus-Akaluc, Group 3 received only PET membranes without cells, and Group 4 received no cells or membranes (Figure 5D). On the day of transplantation, the mice were anesthetized with isoflurane, and a small incision was made in the back for cell transplantation. PET membranes were transplanted subcutaneously with the cell-attached side facing the body surface, and the skin was sutured. After the surgery, the mice were monitored for signs of discomfort or distress.

Measurement of AkaBLI luminescence and serum testosterone levels in mice

Three days after transplantation, we anesthetized the mice again with isoflurane and injected AkaLumine-HCl (750 µg per body) dissolved in 300 µl of saline into the abdominal cavity. After 10 min in the abdominal cavity, we measured the AkaBLI luminescence levels using an IVIS Lumina LT imaging system. The settings were an exposure time of 60 s, medium binning of 8, XFOV field of view measuring 12.9×12.9 cm, and an f/stop value of 1. Mice were euthanized by cervical dislocation, and blood was immediately drawn from their hearts. Subsequently, serum hormone levels were measured at the Integrated Center for Mass Spectrometry at Kobe University (https://www.med.kobe-u.ac.jp/icms/icms/index.html). The skin of the mice was cut open and observed at the engraftment site. We removed the PET membranes and

immunostained them using an anti-LHCGR antibody as described in the immunofluorescence assay section above.

Transplantation of LLC clusters into the mice

Tet-Off LLCs on days 43-67 of differentiation induction, cultured in 6-well plates, were treated with 400 µL/well of 0.5X TrypLE Select and incubated for 2 minutes at 37°C, 5% CO_2 incubator. The cells were then rinsed with 1X PBS and detached using a cell scraper (Sumitomo Bakelite) in a scrubbing manner to form cell clusters. These clusters were then subcutaneously transplanted into anesthetized 8- to 12-week-old immunocompromised female mice, as described above. Three days post-implantation, the mice were euthanized by cervical dislocation, and heart blood samples were collected for the measurement of various hormone levels, as previously described. In experiments involving male mice, five-week-old immunocompromised mice were anesthetized using isoflurane, and the bilateral testes were surgically removed. Tet-Off LLCs were subsequently transplanted subcutaneously in the form of cell clusters as described previously. Four days later, blood samples were collected from the heart, and serum concentrations of various hormones were quantified.

Figures

Several figures were created using the BioRender software program (BioRender.com). These illustrations were used to depict specific experimental processes and results, adhering to the platform's guidelines for academic and scientific use.

Declaration of generative AI and AI-assisted technologies in the writing process

During the preparation of this work the authors used DeepL (https://www.deepl.com/translator) and ChatGPT-4 (https://chat.openai.com/) in order to translate specific sentences from Japanese to English. After using these tools, the authors reviewed and edited the content as needed and take full responsibility for the content of the publication.

SUPPLEMENTAL REFERENCES

Guo, J., Grow, E.J., Mlcochova, H., Maher, G.J., Lindskog, C., Nie, X., Guo, Y., Takei, Y., Yun, J., Cai, L., et al. (2018). The adult human testis transcriptional cell atlas. Cell Res *28*, 1141-1157. 10.1038/s41422-018-0099-2.

Ishida, T., Koyanagi-Aoi, M., Yamamiya, D., Onishi, A., Sato, K., Uehara, K., Fujisawa, M., and Aoi, T. (2021). Differentiation of Human Induced Pluripotent Stem Cells Into Testosterone-Producing Leydig-like Cells. Endocrinology *162*. 10.1210/endocr/bqab202.

Kuroda, T., Yasuda, S., Matsuyama, S., Tano, K., Kusakawa, S., Sawa, Y., Kawamata, S., and Sato, Y. (2015). Highly sensitive droplet digital PCR method for detection of residual undifferentiated cells in cardiomyocytes derived from human pluripotent stem cells. Regen Ther *2*, 17-23. 10.1016/j.reth.2015.08.001.

Murai, N., Koyanagi-Aoi, M., Terashi, H., and Aoi, T. (2023). Re-generation of cytotoxic γδT cells with distinctive signatures from human γδT-derived iPSCs. Stem Cell Reports *18*, 853-868. 10.1016/j.stemcr.2023.02.010.

Nakagawa, M., Taniguchi, Y., Senda, S., Takizawa, N., Ichisaka, T., Asano, K., Morizane, A., Doi, D., Takahashi, J., Nishizawa, M., et al. (2014). A novel efficient feeder-free culture system for the derivation of human induced pluripotent stem cells. Sci Rep *4*, 3594. 10.1038/srep03594.

Schindelin, J., Arganda-Carreras, I., Frise, E., Kaynig, V., Longair, M., Pietzsch, T., Preibisch, S., Rueden, C., Saalfeld, S., Schmid, B., et al. (2012). Fiji: an open-source platform for biological-image analysis. Nat Methods *9*, 676-682. 10.1038/nmeth.2019.
