# Supplementary material for: Efficient differentiation of human iPSCs into Leydig-like cells capable of long-term stable secretion of testosterone
Source: Stem Cell Reports. 2025 Jan 16;20(2):102392. doi: 10.1016/j.stemcr.2024.102392 (PMC11864132; doi:10.1016/j.stemcr.2024.102392)
Supplement: Document S2. Article plus supplemental information [file mmc8.pdf]

Stem Cell Reports
Article
OPEN ACCESS

# Efficient differentiation of human iPSCs into Leydig-like cells capable of long-term stable secretion of testosterone

Katsuya Sato,[1,2,3] Michiyo Koyanagi-Aoi,[1,2,4] Keiichiro Uehara,[1,2,5] Yosuke Yamashita,[1,2,3] Masakazu Shinohara,[6,7] Suji Lee,[8] Anika Reinhardt,[8] Knut Woltjen,[8] Koji Chiba,[3] Hideaki Miyake,[3] Masato Fujisawa,[3] and Takashi Aoi[1,2,4,9,10,*]

[1]Division of Stem Cell Medicine, Graduate School of Medicine, Kobe University, Kobe, Japan
[2]Division of Advanced Medical Science, Graduate School of Science, Technology and Innovation, Kobe University, Kobe, Japan
[3]Division of Urology, Graduate School of Medicine, Kobe University, Kobe, Japan
[4]Center for Human Resource Development for Regenerative Medicine, Kobe University Hospital, Kobe, Japan
[5]Department of Diagnostic Pathology, Graduate School of Medicine, Kobe University, Kobe, Japan
[6]The Integrated Center for Mass Spectrometry, Graduate School of Medicine, Kobe University, Kobe, Japan
[7]Division of Molecular Epidemiology, Graduate School of Medicine, Kobe University, Kobe, Japan
[8]Department of Life Science Frontiers, Center for iPS Cell Research and Application (CiRA), Kyoto University, Kyoto, Japan
[9]Division of Signal Pathways, Biosignal Research Center, Kobe University, Kobe, Japan
[10]Lead contact
*Correspondence: takaaoi@med.kobe-u.ac.jp
https://doi.org/10.1016/j.stemcr.2024.102392

## SUMMARY

Late-onset hypogonadism (LOH) syndrome is characterized by age-related testosterone deficiency and negatively affects the quality of life of older men. A promising therapeutic approach for LOH syndrome is transplantation of testosterone-producing Leydig-like cells (LLCs) derived from human induced pluripotent stem cells (hiPSCs). However, previous studies have encountered obstacles, such as limited cell longevity, insufficient testosterone production, and inefficiency of differentiation. To address these issues, we developed a novel protocol that includes forced *NR5A1* expression, a cytokine cocktail promoting mesoderm differentiation, and a transitional shift from 3D to 2D cultures. The resultant cells survived on culture dishes for over 16 weeks, produced 22-fold more testosterone than the conventional method, and constituted a homogeneous population of LLCs with a differentiation efficiency exceeding 99% without purification. Furthermore, these LLCs were successfully engrafted subcutaneously into mice, resulting in increased serum testosterone levels. Our study will facilitate innovative therapeutic strategies for LOH syndrome.

## INTRODUCTION

The age-related decline in serum levels of the male hormone testosterone is a significant cause of late-onset hypogonadism (LOH) syndrome (Handelsman et al., 2015; Huhtaniemi, 2014; Wu et al., 2008). Clinical manifestations of LOH syndrome include muscle loss, a decreased bone mineral density, increased fat mass, sexual dysfunction, mood disorders, and fatigue, all of which lead to a diminished quality of life in older men (Basaria, 2014). LOH syndrome has also been associated with metabolic syndrome and diabetes (Stellato et al., 2000; Tsujimura et al., 2013) and is emerging as a significant public health concern. Testosterone is mainly secreted by Leydig cells located in the interstitium of the testes (Chamindrani Mendis-Handagama and Siril Ariyaratne, 2001). A decline in the number of Leydig cells with age leads to reduced blood testosterone levels, which in turn causes LOH syndrome (Mularoni et al., 2020).

Currently, testosterone replacement therapy (TRT) is the predominant treatment for LOH syndrome globally (Khera et al., 2016; Shin and Park, 2019). Although TRT effectively improves symptoms, it places a high compliance burden on patients, as its effects are not sustained, so regular treatment must be continued. For example, injectable therapy requires injections every 2–4 weeks (Bhasin et al., 2010). Skin patches cause skin rashes in many patients (Bhasin et al., 2010), and ointments may cause skin rashes and secondary exposure to others (Bhasin et al., 2010). Oral medications require high doses of testosterone due to metabolic effects, and there are concerns about the burden on the liver (Bhasin et al., 2010). For these reasons, alternative treatments to TRT are desirable.

The transplantation of Leydig cells is viewed as a promising novel therapeutic alternative to TRT. If the transplanted Leydig cells remain viable within the body, their efficacy can last for the lifetime of the recipient, or at least significantly longer than the transient efficacy of a single testosterone injection. However, Leydig cells, which are terminally differentiated and do not proliferate *in vitro* (Chen et al., 2009; Haider, 2004), face supply scarcity, hampering their potential use in cell transplantation procedures.

A previous study reported that targeted activation of *NR5A1, GATA4*, and *DMRT1* could convert human foreskin fibroblasts into functional Leydig-like cells (LLCs); however, its reprogramming efficiency was approximately 7% (Huang et al., 2019). In addition, a method for isolating


Published by Elsevier Inc. on behalf of International Society for Stem Cell Research.
This is an open access article under the CC BY license (http://creativecommons.org/licenses/by/4.0/).

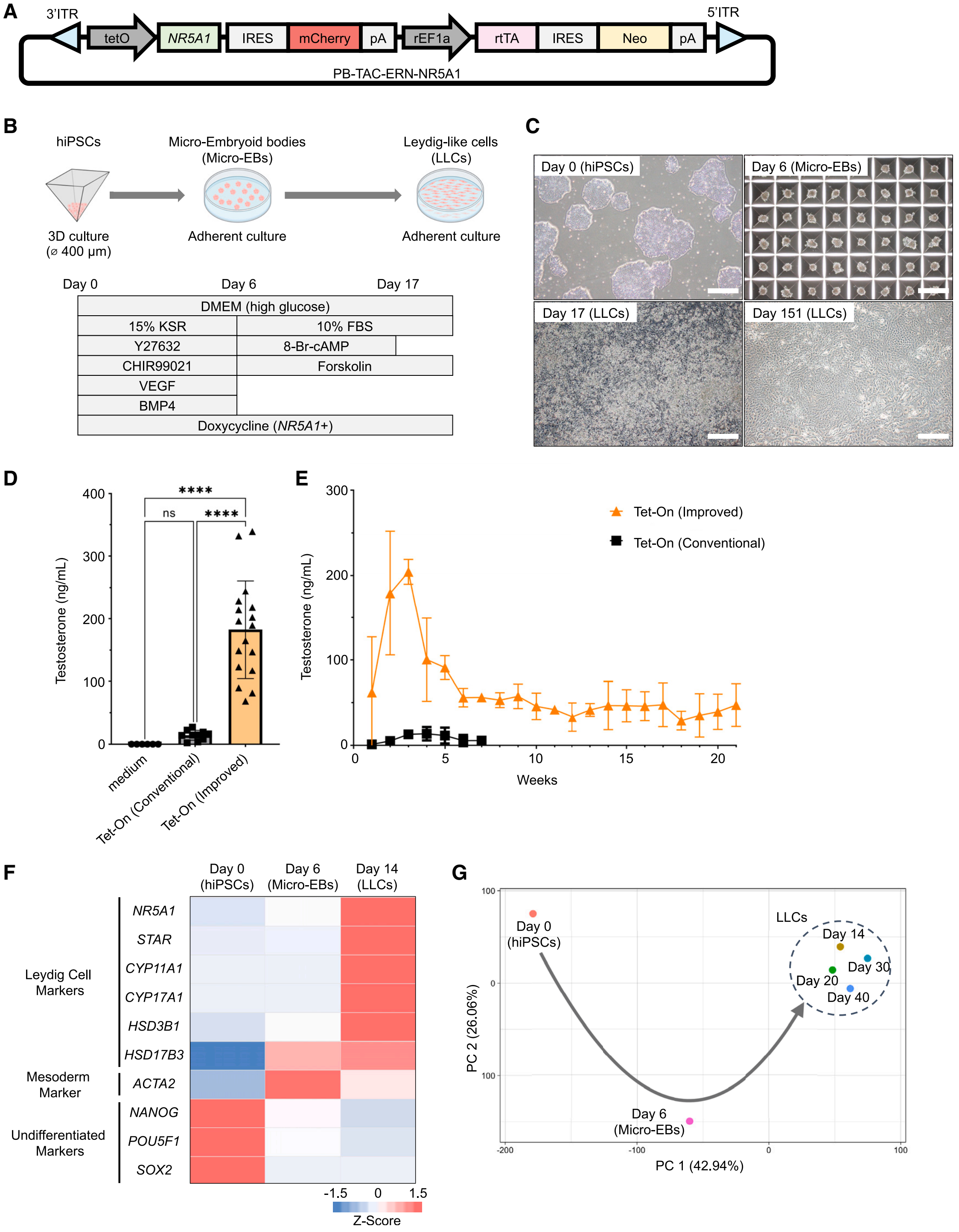


**Figure 1. Improved method of generating hiPSC-derived LLCs**

(A) Doxycycline-inducible *NR5A1* piggyBac vector. *NR5A1* and mCherry were combined with an IRES such that when exogenous *NR5A1* was expressed, the fluorescent protein mCherry was co-expressed.

(B) Schematic representation of the improved differentiation protocol for generating LLCs from hiPSCs. This protocol is characterized by the constitutive forced expression of *NR5A1*, switching from 3D to adherent culture, and addition of compounds to promote mesoderm differentiation.

*(legend continued on next page)*

human stem Leydig cells (hSLCs) from human testes and generating proliferating Leydig cells from these hSLCs has also been reported, but ethical issues surrounding the procurement of hSLCs and the clinical application of this method pose significant challenges (Feng et al., 2021).

Several groups, including ours, have investigated methods for generating testosterone-secreting LLCs from stem cells (Chen et al., 2019, Ishida et al., 2021; Ji et al., 2020; Li et al., 2019; Shin et al., 2021, Yang et al., 2015). Based on reports that *NR5A1* is a master regulator required for Leydig cell differentiation (Luo et al., 1994; Yang et al., 2015), we successfully generated functional testosterone-secreting LLCs by forced expression of *NR5A1* in human induced pluripotent stem cells (hiPSCs) and employed three-dimensional (3D) culture to form embryoid bodies (EBs) (Ishida et al., 2021). However, the generated cells could be maintained for approximately 7 weeks only, and given that the Tet-On system was utilized to maintain the expression of *NR5A1*, it was necessary to consistently administer doxycycline to the cells. Another group reported a differentiation induction method to induce differentiation of LLCs from hiPSCs without gene overexpression, using only the addition of compounds (Chen et al., 2019). However, the protocol consisted of six rather complex steps, and the generated cells produced a smaller amount of testosterone than our protocol, with the differentiation induction efficiency being low (about 50%). In addition, there are issues regarding the selection of cell transplantation sites. For example, if LLCs can be engrafted subcutaneously, the wound associated with transplantation would be small and simple to induce, and if the cells become cancerous, they could be easily removed. However, current methods of engraftment of cells into the interstitium of the testis (Chen et al., 2019; Curley et al., 2019; Yang et al., 2015, 2020) or into the peritoneal cavity (Feng et al., 2021) do not provide this advantage and thus present a significant challenge.

In this study, we established a novel and improved protocol that involves the use of a Tet-Off system (no need for doxycycline to maintain forced expression of exogenous genes) for the overexpression of *NR5A1* in hiPSCs, the addition of cytokine cocktails to enhance mesoderm differentiation, and the transitional shift from 3D to two-dimensional (2D) culture system. This approach has enabled us to generate LLCs that secrete testosterone at concentrations approximately 22 times higher than those achieved with the conventional method (Ishida et al., 2021), with these LLCs having a lifespan exceeding 16 weeks in culture dishes. We also successfully achieved an LLC differentiation efficiency of nearly 100% without the need for purification. Contrary to the previous approach of injecting LLCs into the testis, with a clinical perspective in mind, we also successfully elevated serum testosterone levels by engrafting clusters of LLCs into the subcutaneous tissues of castrated mice, an approach that is more readily applicable in clinical practice.

## RESULTS

### Establishment of a novel method of LLC differentiation with high testosterone secretion and long-term viability

Based on a previously reported protocol in which *NR5A1* was overexpressed using the Tet-On system (Figure 1A) (Ishida et al., 2021), we optimized the method using testosterone concentrations in the culture supernatant as an indicator by improving four points (1)–(4). The protocol of the improved method and the cell morphological changes during differentiation are shown in Figures 1B and 1C, respectively.

(1) Although we used a 96-well plate with a diameter of 7 mm per well from days 0 to 6 to form EBs in the conventional method (Figures S1A and S1B), we employed a plate with numerous open microwells 400 μm diameter to increase the number of micro-EBs generated in the improved method (Figures 1B and 1C).

(C) Morphological changes in cells generated using the improved method of differentiation induction. Cells at day 0 (hiPSCs), day 6 (micro-EBs), day 17, and day 151 (LLCs) were observed using phase-contrast microscopy. Scale bars: 500 μm.
(D) Testosterone concentrations in the culture supernatant were measured using the electrochemiluminescence immunoassay (ECLIA) method. Testosterone concentrations represent the peak levels observed during the measurement period. The testosterone concentration peaked value on days 21–37 (median: day 28) for the conventional method and on days 15–26 (median: day 18) for the improved method. The findings are expressed as the mean ± standard deviation (SD) from independent experiments, with sample sizes of $n = 6$ for the control (medium) group, $n = 10$ for the conventional method group, and $n = 17$ for the improved method group. Statistical significance is denoted by **** for $p < 0.0001$.
(E) The comparison of changes in testosterone concentrations in culture supernatants during differentiation induction between the improved and the conventional methods. Mean value ± SD ($n = 3$ independent experiments).
(F) A heatmap illustrating changes in the expression of Leydig cell markers, a mesoderm marker, and undifferentiated markers using NGS. The gene expression was depicted with color-coded representations derived from $Z$ scores calculated from transcript per million (TPM) values.
(G) A principal component analysis of NGS data from day 0 (hiPSCs), day 6 (micro-EBs), and LLCs (days 14, 20, 30, and 40) is presented. The horizontal axis represents the score of principal component 1, whereas the vertical axis represents the score of principal component 2.

(2) Because Leydig cells are mesodermal cells (Merchant-Larios and Moreno-Mendoza, 1998), we added CHIR99021, VEGF, and BMP4, which are known to enhance mesoderm differentiation (Ohta et al., 2016), from days 0 to 6.
(3) Contrary to the conventional method, which continued to culture EBs in 3D culture (Ishida et al., 2021), we intentionally transitioned the mesodermal lineage micro-EBs from 3D culture to adherent culture on day 6 (Figures 1B and 1C). The EBs then adhered to the bottom of the culture dish and lost their spherical shape, and each individual cell migrated and divided into the surrounding spaces (Video S1).
(4) From day 6 onward, we supplemented the medium with 8-bromoadenosine-3′,5′-cyclic monophosphate (8-Br-cAMP) and forskolin, as in the conventional method (Figures 1B and S1A) (Ishida et al., 2021; Yang et al., 2015).

The LLCs generated by the improved method had an average peak testosterone concentration in the culture supernatant that was approximately 12.8 times higher than that of the LLCs generated by the conventional method (Figure 1D; Table S1). Unlike the LLCs derived from the conventional method, which died after approximately 7 weeks due to loss of their embryoid shape, those derived via the improved method survived on culture dishes for at least 21 weeks while secreting testosterone stably (Figure 1E).

### Comprehensive gene expression changes during the process of differentiation into LLCs and differentiation efficiency

Next, we analyzed the changes in comprehensive gene expression during the differentiation process from hiPSCs to LLCs in the current method using RNA sequencing. At the onset of differentiation induction (day 0), we observed the expression of undifferentiated marker genes, such as *POU5F1*, *SOX2*, and *NANOG* (Figure 1F). As differentiation proceeded, we detected a significant increase in the expression of Leydig cell markers, such as *STAR*, *CYP11A1*, *CYP17A1*, *HSD3B1*, and *HSD17B3*, by day 14. This result was consistent with the increased testosterone secretion in LLCs after day 14 of differentiation induction (Figure 1E). A principal component analysis (PCA) revealed that the LLCs generated on days 14, 20, 30, and 40 detected similar global gene expression patterns, suggesting that Leydig cell differentiation was likely accomplished by day 14 (Figure 1G).

To examine the characteristics of the genes that showed an upregulated expression in LLCs on day 14 compared to induced pluripotent stem cells (iPSCs), we selected 239 genes (higher expression in LLCs with a fold change >30) and performed a pathway analysis (Figures S2A and S2B; Table S2). This analysis for these genes suggested that, in addition to Leydig cell markers, the expression of genes related to steroidogenesis and androgen synthesis, which are characteristic functions of Leydig cells, increased.

Because of their pluripotency, iPSCs can differentiate into various cell types, including unintended cell types (Takahashi and Yamanaka, 2006). Therefore, it is essential to achieve high differentiation efficiency when generating specific cell types from iPSCs for clinical applications. Previous reports have shown that <50% of LLCs from human iPSCs express Leydig cell markers (Chen et al., 2019). Furthermore, even with our conventional differentiation induction protocol, the generated cell population produced not only testosterone but also aldosterone and cortisol, suggesting the presence of non-target cells, such as adrenal cortex cells (Ishida et al., 2021).

We performed immunofluorescence to determine the differentiation efficiency of LLCs in the cell population generated using the current method (Figures S2C and S2D). All of the examined biomarkers for Leydig cells (HSD17B3, LHCGR, STAR, and CYP17A1) showed high positivity rates, particularly HSD17B3 and LHCGR, with a positivity rate of over 99%. A flow cytometry analysis also revealed high positivity rates for biomarkers of Leydig cells, with LHCGR exhibiting the highest rate at 98.31% (Figures S2E and S2F). These results demonstrated that our current protocol can effectively differentiate hiPSCs into LLCs, resulting in a homogeneous cell population without the need for purification.

### Generation of LLCs using the forced expression of *NR5A1* via the Tet-Off system

We previously generated LLCs by continuously expressing *NR5A1*, a master regulator for differentiation into Leydig cells (Luo et al., 1994; Yang et al., 2015), using the Tet-On system (Ishida et al., 2021). To investigate whether or not the continuous expression of *NR5A1* is necessary for LLCs to continue to secrete testosterone, we removed doxycycline from the culture medium after differentiation and examined the amount of testosterone produced. We monitored the expression of exogenous *NR5A1* by fluorescence observation of mCherry (Figure 1A) and confirmed a decrease in its expression 11 days after doxycycline removal (Figure S3A). In addition, the amount of testosterone also decreased and reached almost zero after 1 week (Figure 2A). These results suggest that *NR5A1* is essential not only for differentiation into Leydig cells but also for maintaining the Leydig cell function.

Considering the prospective application of LLCs in future transplantation therapies, it is undesirable to have to continue adding doxycycline in order to sustain the expression of *NR5A1*. Therefore, we opted to utilize

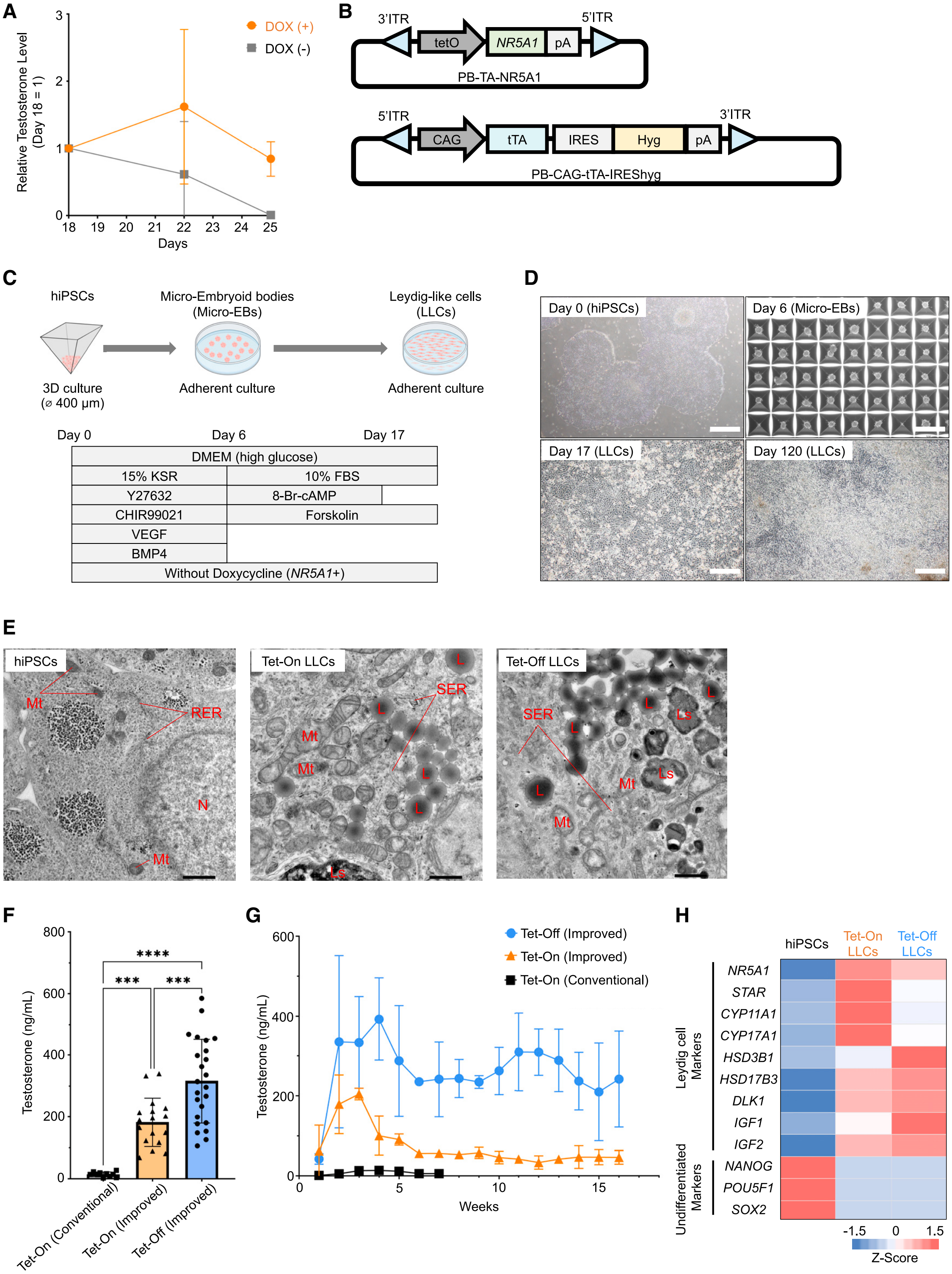


**Figure 2. Generation of hiPSC-derived LLCs using the Tet-Off System**

(A) LLCs generated by the forced expression of *NR5A1* using the Tet-On system exhibited a decrease in testosterone production in the absence of doxycycline. In the DOX (+) group, 1.5 μM doxycycline was continuously added to the medium, whereas in the DOX (−) group, doxycycline was removed after day 18. The concentration of testosterone in the culture supernatant on day 18 is represented as a ratio of 1. Mean value ± SD ($n = 3$ independent experiments).

*(legend continued on next page)*

the Tet-Off system to induce the expression of *NR5A1* without the addition of doxycycline (Figure 2B) (Gossen and Bujard, 1992). We attempted to induce differentiation into LLCs using the same protocol as the Tet-On system, except that doxycycline was added until the start of differentiation induction and then removed (Figures 1B and 2C). In differentiation using the Tet-Off system, micro-EBs were formed by day 6, and the cell morphologies of differentiated cells after switching to adherent culture were similar to those generated using the Tet-On system (Figures 1C and 2D). Previous reports have indicated that rat Leydig cells are characterized by well-developed organelles such as lipid droplets and smooth endoplasmic reticulum, reflecting their function in synthesizing testosterone from cholesterol (Chen et al., 2009). We performed electron microscopy to analyze three cell types: hiPSCs, LLCs generated by the Tet-On system (Tet-On LLCs), and LLCs generated by the Tet-Off system (Tet-Off LLCs). Electron microscopy showed that organelles such as lipid droplets (L) and smooth endoplasmic reticulum were abundantly present in the cytoplasm of both Tet-On LLCs (Figure 2E middle panel) and Tet-Off LLCs (Figure 2E right panel), but not in the hiPSC stage (Figure 2E left panel). These data suggest that LLCs are similar to Leydig cells in terms of their internal cellular structure.

Unexpectedly, the concentration of testosterone in the culture supernatant was significantly higher in Tet-Off LLCs than Tet-On LLCs, and the concentration of testosterone in the culture supernatant of Tet-Off LLCs was approximately 22 times higher than that of LLCs generated by our conventional method (Table S1; Figure 2F). Testosterone secretion by 1 million Tet-Off LLCs surpassed 200 ng over 24 h (Figure S3B). In addition, Tet-Off LLCs demonstrated the ability to survive for more than 16 weeks while secreting large amounts of testosterone on culture dishes (Figure 2G).

We also performed semiquantitative reverse-transcription polymerase chain reaction to examine the expression of Leydig cell marker genes in Tet-On and Tet-Off LLCs (Figure S3C). The results showed that both LLCs expressed steroidogenic enzyme genes (*STAR*, *CYP11A1*, *CYP17A1*, *HSD3B1*, and *HSD17B3*), all of which are required for the synthesis of steroid hormones from cholesterol. A comprehensive gene expression analysis using next-generation sequencing (NGS) detected the expression of Leydig cell markers in LLCs generated by both the Tet-On and Tet-Off systems (Figure 2H). A WikiPathways analysis was employed to examine the 239 genes that exhibited an increased expression in Tet-Off LLCs compared to that in hiPSCs (Figures S3D and S3E; Table S2). The results revealed that the pathway related to "androgen synthesis" was the top pathway, which was consistent with the analysis of Tet-On LLCs (Figure S2B). A PCA showed that Tet-On and Tet-Off LLCs were located close to each other, indicating that their comprehensive gene expression patterns were similar (Figure S3F). There were subtle differences between Tet-On and Tet-Off LLCs in each experiment (Figures 2F–2H), which may be due to variations in *NR5A1* gene transfer efficiency and promoter activity between the experimental systems. These variations likely resulted in the differences in the expression levels of various Leydig cell markers and testosterone concentrations.

## Single-cell RNA sequencing revealed similarities between the generated LLCs and authentic Leydig cells

To assess the molecular similarity of our generated Tet-On and Tet-Off LLCs to authentic Leydig cells, we performed

(B) Construction of a piggyBac vector for the *NR5A1* expression in the absence of doxycycline (using the Tet-Off system).
(C) A schematic representation of the protocol for inducing the differentiation of hiPSCs to LLCs is shown. To induce *NR5A1* expression using the Tet-Off system, doxycycline was removed during the differentiation-induction process.
(D) Morphological changes in the cells were captured by phase-contrast microscopy on day 0 (hiPSCs), day 6 (micro-EBs), day 17, and day 120 (LLCs). Scale bars: 500 μm.
(E) Electron micrographs of hiPSCs (left), Tet-On LLCs (middle), and Tet-Off LLCs (right) are shown. The abbreviations for the organelles are as follows: N, nucleus; Mt, mitochondria; L, lipid droplets; LS, lysosomes; SER, smooth endoplasmic reticulum; RER, rough endoplasmic reticulum. Scale bars: 1,000 nm.
(F) The testosterone concentration in the culture supernatant was measured using the ECLIA method. Testosterone concentrations represent the peak levels observed during the induction period. Peak testosterone concentrations were recorded on days 21–37 (median: day 28) for the Tet-On conventional group, on days 15–26 (median: day 18) for the Tet-On improved group, and on days 18–90 (median: day 32) for the Tet-Off improved group. Results are represented as the mean ± SD from independent experiments, with sample sizes of $n = 10$ for the Tet-On conventional group, $n = 17$ for the Tet-On improved group, and $n = 24$ for the Tet-Off improved group. Statistical significance was denoted by **** for $p < 0.0001$ and *** for $p < 0.001$.
(G) Testosterone concentrations in the culture supernatants during differentiation induction were compared between the groups. Mean value ± SD ($n = 3$ independent experiments).
(H) A heatmap displays variations in expression of Leydig cell markers and undifferentiated markers using NGS, represented by color-coded scales derived from *Z* scores calculated from transcripts per million (TPM) values. The analysis included hiPSCs on day 0, Tet-On LLCs on day 30, and Tet-Off LLCs on day 35.

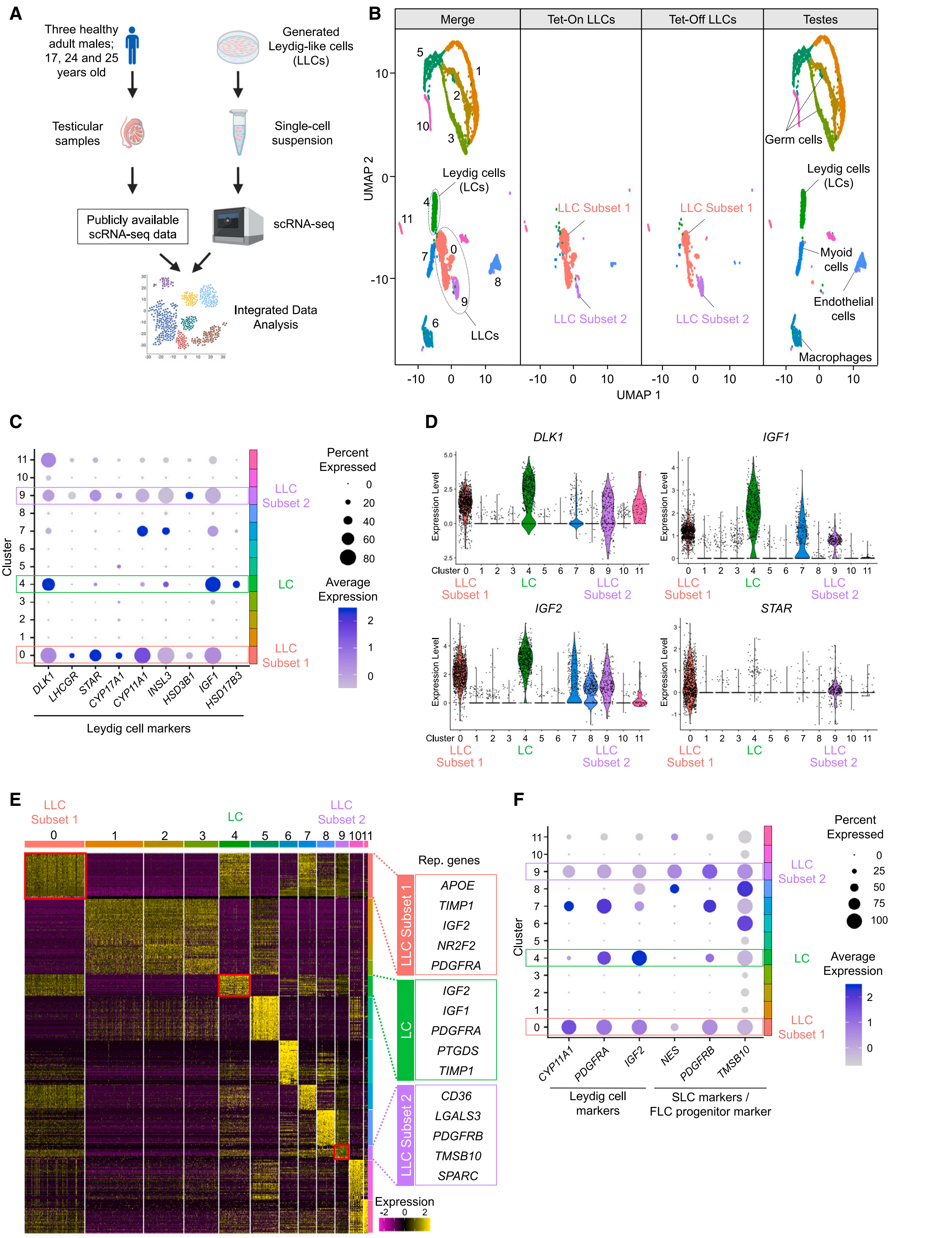


*(legend on next page)*

a single-cell RNA sequencing (scRNA-seq) analysis. We integrated publicly accessible scRNA-seq data from testicular samples obtained from three healthy male subjects using scRNA-seq data from our generated cells (Figure 3A). Using the integrated dataset, we generated uniform manifold approximation and projection (UMAP) plots and then identified cell clusters based on marker genes distinctive of Leydig cells (cluster 4), myoid cells (cluster 7), macrophages (cluster 6), germ cells (clusters 1, 2, 3, 5, and 10), and vascular endothelial cells (cluster 8) (Figures 3B and S4A). Our generated Tet-On and Tet-Off LLCs were divided into LLC subset 1 (cluster 0) and LLC subset 2 (cluster 9), which were close to authentic Leydig cells on UMAP, indicating their similar gene expression patterns. We selected genes characteristic of Leydig cells and illustrated the expression status of these genes in each cluster using a dotplot (Figure 3C) and violin plots (Figure 3D). The expression patterns of Leydig cell marker genes in LLC subsets 1 and 2 were similar to those of authentic Leydig cells. The heatmap further demonstrated concordance in the gene expression status between LLCs and authentic Leydig cells (Figure 3E).

We also extracted the top 50 most distinctive genes for each cluster (Table S3), and the representative genes are shown in Figure 3E right panels. In LLC subset 1, genes such as *TIMP1*, *IGF2*, and *PDGFRA* as well as authentic Leydig cells exhibited a high expression, whereas markers such as *PDGFRB* (Li et al., 2022a; Odeh et al., 2014), a marker for stem Leydig cells (SLCs), and *TMSB10* (Inoue et al., 2022), a marker for fetal Leydig cell (FLC) progenitors, were elevated in LLC subset 2. In addition, when we examined the genes that showed a differential expression between LLC subsets 1 and 2, we found that the expression of *NES*, a characteristic marker of SLCs (Davidoff et al., 2004; Jiang et al., 2014; Yao et al., 2022), was higher in subset 2 than in subset 1 (Table S4; Figure S4B). A dotplot analysis showed that the expression of *PDGFRB*, *NES*, and *TMSB10* was more prominent in LLC subset 2 than in subset 1 (Figure 3F). We compared the publicly available single-cell analysis data of human fetal testicular cells (Chitiashvili et al., 2020) with the data from LLCs, and UMAP showed that these cell groups were positioned apart (data not shown). Although LLCs expressed SLC markers and FLC progenitor markers more than Leydig cells (Figure 3F), we found that the gene expression profiles of fetal testicular cells and LLCs were not identical. We performed a dotplot analysis to further investigate the markers related to cell division. It revealed that proliferation markers, such as *MKI67* and *PCNA*, were expressed at higher levels in LLCs than in authentic Leydig cells (Figure S4C). The top 100 genes that were differentially expressed between LLCs and Leydig cells were identified (Table S4). A pathway analysis based on this gene list revealed that when compared to Leydig cell, pathways related to steroidogenic enzymes were significantly upregulated in LLC subset 1, and pathways related to cell division were significantly upregulated in LLC subset 2.

In addition, we analyzed cell samples at each time point of differentiation induction (on days 0, 6, 14, and 28) and created a UMAP (Figure S4D). As differentiation induction progressed, *DLK1*, a Leydig cell marker gene, was upregulated (Figure S4E). We also assessed the expression of *SOX17* (an endodermal marker), *ACTA2* (a mesodermal marker), and *SOX1* (an ectodermal marker). We observed that *SOX17* and *SOX1* were not expressed during differentiation induction, whereas *ACTA2* expression was increased on day 6, which was consistent with the results of the NGS analysis (Figure 1F). Given that Leydig cells are derived from the mesoderm, upregulation of *ACTA2* expression during differentiation induction is biologically plausible.

## Measurement of the differentiation efficiency and undifferentiated marker genes in Tet-Off LLCs and the analysis of passaging and freeze-thaw cycles

As in the Tet-On system, we investigated whether or not the induction efficiency was also high when the Tet-Off system

**Figure 3. Genetic analyses of LLCs using scRNA-seq**

(A) Steps for single-cell analyses: the data acquired from the generated LLCs were merged with previously published testicular data from healthy males, and the integrated data were then analyzed.

(B) UMAP plots were created from the integrated data to visualize the distribution of the gene expression profiles. Presented from left to right are the UMAP plots of the integrated data, Tet-On LLCs on P0 day 14, Tet-Off LLCs on P2 day 18, and testes.

(C) Gene expression characteristics of Leydig cells were compared among the identified clusters and represented using dotplots.

(D) Gene expression characteristics of Leydig cells were compared among the identified clusters and represented using violin plots.

(E) A heatmap was used to visualize the gene characteristics of each cluster, with the vertical axis representing genes and the horizontal axis corresponding to the clusters. Genes with an elevated expression appear yellow, whereas those with a decreased expression appear purple. Representative genes that are characteristically expressed in the LC, LLC subset 1, and LLC subset 2 clusters are listed on the right side (see also Table S3).

(F) Genes differentially expressed between LLC subsets 1 and 2 were identified and visualized using dotplots. The Leydig cell markers *CYP11A1*, *PDGFRA*, and *IGF2* were highly expressed in subset 1. In contrast, the SLC markers *NES* and *PDGFRB*, as well as *TMSB10*, a gene involved in fetal Leydig cell (FLC) differentiation, were upregulated in subset 2.

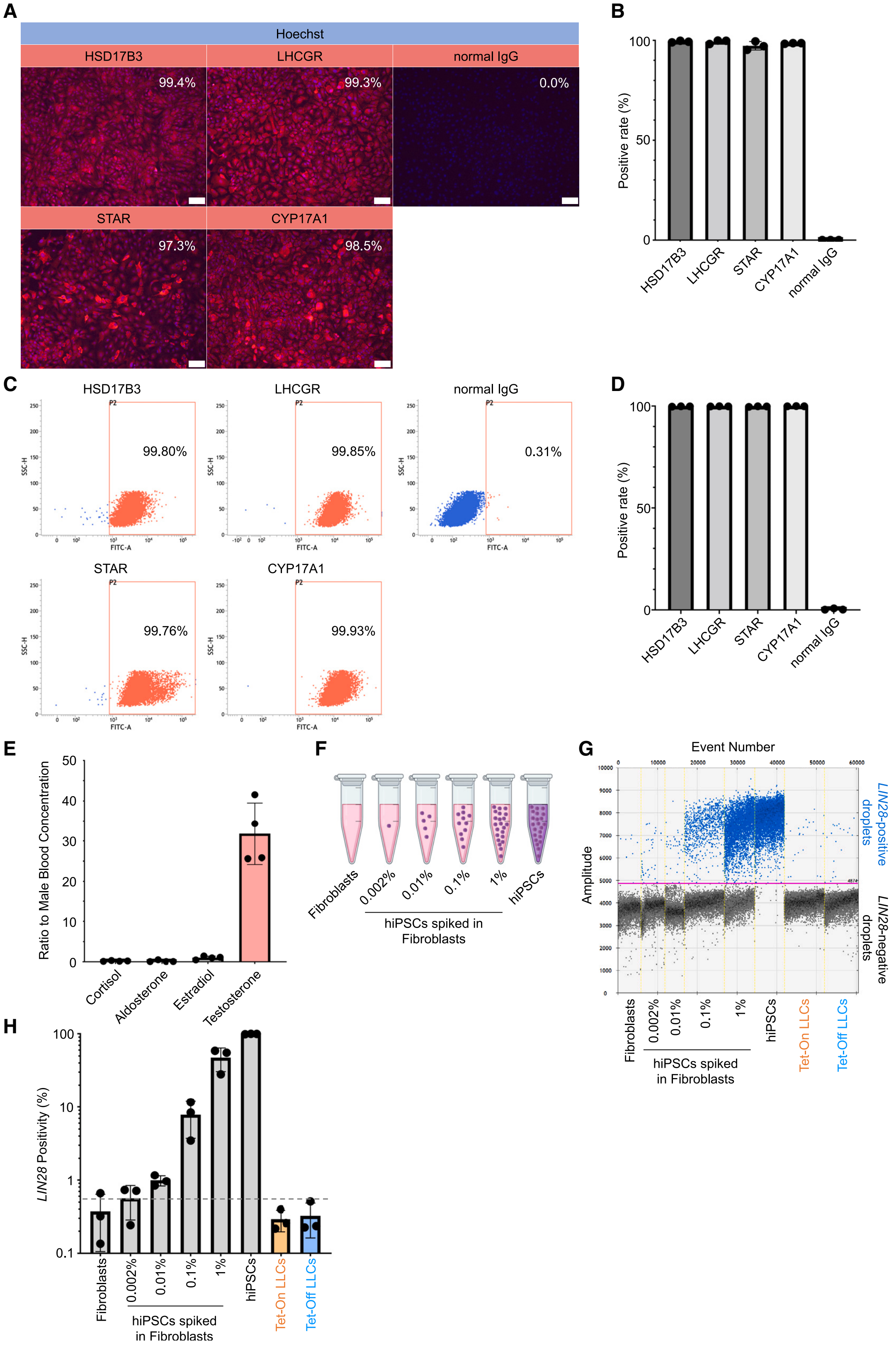


*(legend on next page)*

was used. Both immunofluorescence (Figures 4A and 4B) and flow cytometry analyses (Figures 4C and 4D) showed that the percentage of cells expressing the Leydig cell markers exceeded 97%, indicating that even with the forced expression of *NR5A1* by the Tet-Off system, hiPSCs can be effectively differentiated into homogeneous LLC populations without further purification. Furthermore, the cells generated using the conventional method contained aldosterone and cortisol in their culture supernatant (Ishida et al., 2021). In contrast, the LLCs generated by the current method secreted little aldosterone or cortisol in their culture supernatants. The testosterone concentration in the supernatant was approximately 30-fold higher than the normal level in healthy male blood, whereas the concentrations of cortisol, aldosterone, and estradiol were 0.19, 0.17, and 1.01 times higher than the normal levels in healthy male blood, respectively (Table S5; Figure 4E). These results indicate that our current method induces the expression of *NR5A1* in hiPSCs while promoting intensive differentiation into Leydig cells without concomitant differentiation into adrenocortical cells or other gonadal cells.

The presence of residual undifferentiated cells within the final cellular product derived from hiPSCs can lead to uncontrolled proliferation and formation of teratomas (Knoepfler, 2009; Koyanagi-Aoi et al., 2013). To quantify the presence of undifferentiated cells within the generated cell population, we employed the *LIN28A*/digital droplet PCR (ddPCR) method (Kuroda et al., 2015) using *LIN28A* as a marker for undifferentiated cells (Yu et al., 2007). As in previous reports, fibroblasts were used as negative controls for *LIN28A*, and hiPSCs were used as positive controls (Kuroda et al., 2015). Fibroblast samples as negative controls were mixed with increasing concentrations of hiPSCs (0.002%, 0.01%, 0.1%, and 1%, Figure 4F). The results demonstrated that the *LIN28A*-positive droplet rates of both the generated Tet-On LLCs and Tet-Off LLCs were below the 0.002% threshold for the hiPSC contamination rate, indicating a low risk of teratoma formation after transplantation (Figures 4G and 4H).

In our current method, EBs derived from hiPSCs lose their shape after transition from a 3D culture to an adherent culture, and LLCs reach confluence at least 17 days after the start of induction. To examine whether or not the generated LLCs could be passaged and expanded while maintaining their function, we performed passaging after the cells reached confluence (Figure S5A). LLCs were able to proliferate while maintaining their cell morphology and testosterone secretion function for at least three passages (Figures S5B–S5D). Furthermore, we confirmed that LLCs could also be cryopreserved, thawed, and cultured again while maintaining their testosterone secretion (Figure S5E). When considering clinical applications, these features may facilitate the storage of cells intended for transplantation, making it possible to treat these cells as off-the-shelf products.

## Response of LLCs to gonadotropic hormones

We assessed whether the addition of gonadotropic hormones, such as luteinizing hormone (LH) and human chorionic gonadotropin (hCG), to the culture supernatant would increase testosterone secretion by LLCs. The testosterone concentration secreted within 3 h after the addition of LH or hCG showed no significant difference from the control in both Tet-On LLCs (Figure S6A) and Tet-Off LLCs (Figure S6B). Thus, we conclude that our induced LLCs secrete testosterone autonomously, without regulation by gonadotropic hormones. To investigate why LHCGR does not respond to the gonadotropic hormone LH, even though the expression is detected, we considered the possibility that LHCGR, the receptor for LH and hCG, is not localized on the cell surface. We performed immunostaining for LHCGR, with or without

**Figure 4. Evaluation of Tet-Off LLCs by immunostaining, flow cytometry, and ddPCR**

(A) Representative images for the expression of Leydig cell markers in differentiated LLCs on day 7 after two passages using immunofluorescence assays. Scale bars: 100 μm.

(B) Statistical analyses of the immunofluorescence assay. Positivity was quantitatively evaluated using the ImageJ software program. Mean value ± SD ($n$ = 3 independent experiments).

(C) Representative flow cytometry histograms for Leydig cell markers in differentiated LLCs on day 4 after one passage.

(D) Statistical analyses of flow cytometry. Mean value ± SD ($n$ = 3 independent experiments).

(E) Cortisol, aldosterone, estradiol, and testosterone concentrations were measured in the culture supernatant on days 38–42 of cell culture. These concentrations were divided according to the corresponding upper reference limits of male blood concentrations. Mean value ± SD ($n$ = 4 independent experiments).

(F) Detection of *LIN28A* using ddPCR. hiPSCs were used as positive controls, while fibroblasts were used as negative controls. Fibroblast samples were mixed with increasing concentrations of hiPSCs (0.002%, 0.01%, 0.1%, and 1%).

(G) Quantification of positive droplets using ddPCR. The magenta line indicates the fluorescence positivity boundary of the droplet, classifying the droplet as *LIN28A* positive or *LIN28A* negative. The blue dots represent droplets containing at least one copy of *LIN28A* being evaluated.

(H) Statistical analyses of the ddPCR assay. The vertical axis represents the percentage of *LIN28A*-positive droplets. The dotted line indicates the rate of *LIN28A* positivity in the samples containing 0.002% hiPSCs. Mean ± SD, $n$ = 3 independent experiments.

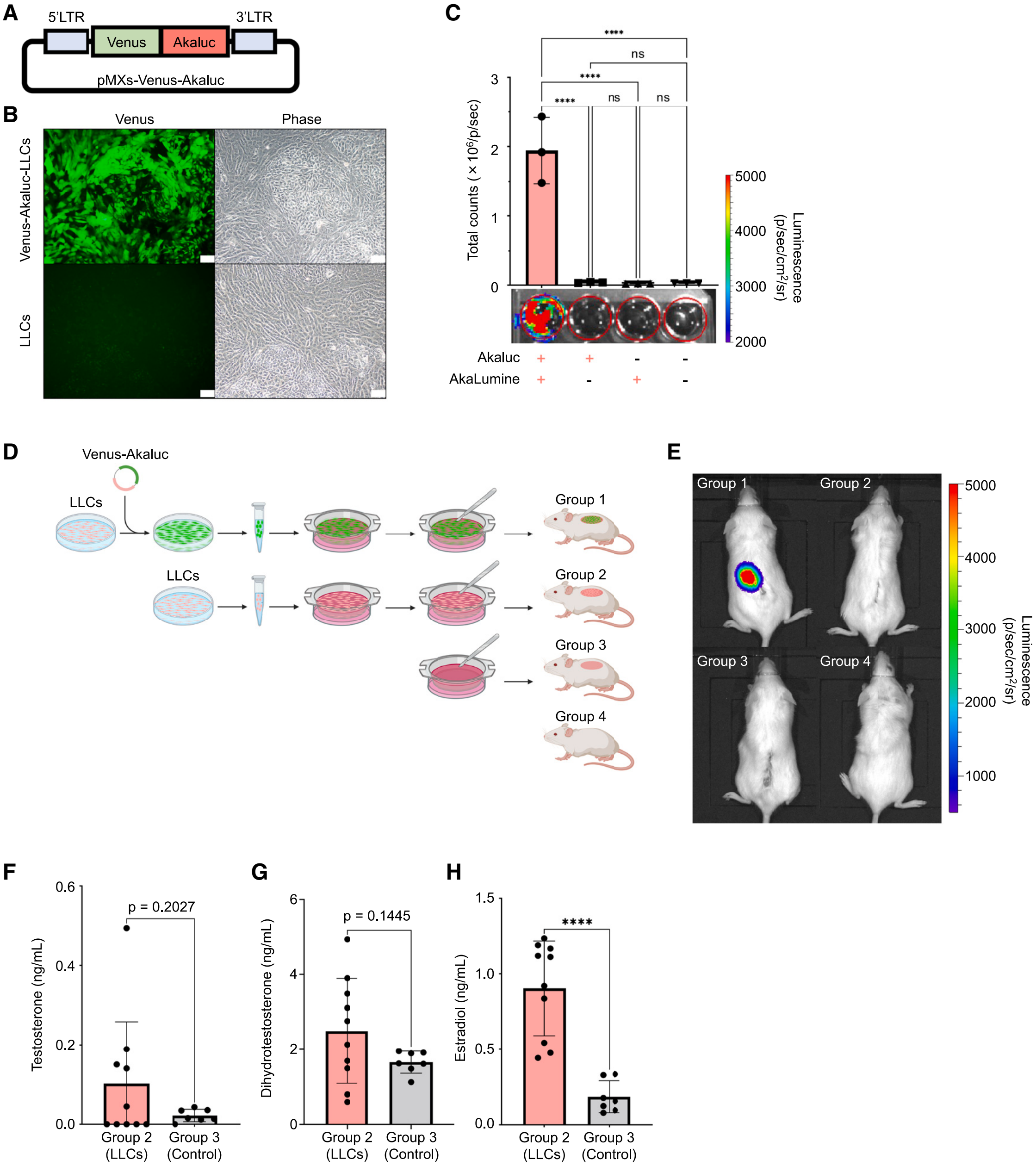


**Figure 5. Transplantation of LLCs into immunodeficient mice**

(A) Diagram of a retroviral vector to introduce Venus-Akaluc expression into Tet-Off LLCs.

(B) Fluorescence (left) and phase-contrast (right) images of LLCs after retroviral transduction with Venus-Akaluc (upper) and control LLCs (lower). Scale bars; 100 μm.

(C) When the substrate AkaLumine was added to the culture wells of LLCs transfected with Venus-Akaluc, luminescence of AkaBLI was detected after 3 min by the substrate enzyme reaction. Images of each group are shown. Quantification of the AkaBLI luminescence was performed in each well. Total counts are the sum of all counts for all pixels inside the region of interest (ROI), presented as the mean ± SD, $n$ = 3 independent experiments. Statistical significance is denoted by **** for $p < 0.0001$.

(D) A schematic diagram of the cell transplantation process in mice is shown. Group 1 mice were transplanted subcutaneously using PET membranes to which Venus-Akaluc-transfected LLCs were attached; group 2 mice were transplanted subcutaneously using PET membranes to which Venus-Akaluc-untransfected LLCs were attached; group 3 mice were implanted subcutaneously with PET membranes alone, with no transplanted cells; and group 4 mice did not have any implants.

*(legend continued on next page)*

membrane permeabilization. The results indicated that nearly 100% of the cells were positive for LHCGR when permeabilized, whereas the percentage of LHCGR-positive cells decreased to approximately 30% without permeabilization (Figures S6C and S6D). These findings suggest that the limited expression of LHCGR on the cell surface leads to the inability of LLCs to respond to gonadotropic hormones.

### Engraftment of LLCs into immunocompromised mice

To investigate whether or not the induced LLCs function as testosterone-secreting cells *in vivo*, we transplanted them into immunodeficient mice. First, we suspended the cells in medium, injected them subcutaneously or intraperitoneally, and measured the blood testosterone concentration. However, no significant increases were observed. Next, we generated LLCs that expressed the fluorescent protein Venus (Nagai et al., 2002) and the bioluminescent enzyme Akaluc (Iwano et al., 2018) to visualize whether or not LLCs were engrafted *in vivo* by retroviral transduction (Figure 5A). LLCs transduced with Venus-Akaluc exhibited distinctive green fluorescence (Figure 5B). Akaluc-transfected LLCs showed a bioluminescent signal, AkaBLI, only when an AkaLumine-HCl substrate was added to the culture medium (Figure 5C).

Because the LLCs were cultured in an adherent manner, to create a similar environment *in vivo*, we attempted to seed LLCs onto a polyethylene terephthalate (PET) membrane and transplanted them together (Figure 5D). Mice in group 1 were transplanted with PET membranes carrying LLCs transfected with Venus-Akaluc, whereas mice in group 2 were transplanted with PET membranes carrying LLCs that lacked Venus-Akaluc. Mice in group 3 received only PET membranes, lacking any cells, and mice in group 4 received neither cells nor PET membranes (Figure 5D). The LLCs consistently exhibited green fluorescence for at least 2 days, even after being attached to the PET membrane (Figure S6E), and were then transplanted subcutaneously into mice. Three days after transplantation, the AkaLumine-HCl substrate was intraperitoneally injected into the mice, resulting in the observation of a bioluminescent signal coincident with the transplantation site (Figure 5E). In the culture supernatant of group 1 LLCs, testosterone levels were significantly lower than those in group 2 LLCs (data not shown). It is hypothesized that this reduction may be due to cell damage incurred during retroviral introduction of Venus-Akaluc into LLCs. Consequently, the focus was shifted to group 2 grafts, which are intended to increase serum testosterone levels after transplantation into mice. Transplantation of group 2 LLCs into female mice tended to increase the levels of serum testosterone and its metabolite, dihydrotestosterone (DHT), whereas a significant increase was observed in estradiol, another metabolite of testosterone (Table S5; Figures 5F–5H). Upon excision of the skin and subsequent observation, subcutaneous angiogenic vessels were observed at the engraftment site, suggesting that an environment had been established in which testosterone secreted by LLCs could easily circulate into the mouse bloodstream (Figure S6F). Angiogenesis was observed at the site of implantation, even when the artificial membrane was implanted alone (group 3, data not shown), suggesting that angiogenesis was not induced by the cells. Furthermore, immunostaining of PET membranes removed from mice revealed the presence of cells positive for LHCGR, a marker of Leydig cells (Figure S6G). In summary, LLCs adhering to PET membranes were viable *in vivo*, with blood levels of testosterone not showing significant elevation, whereas levels of metabolites were significantly elevated.

Next, we attempted to peel off the LLCs with a cell scraper (Figure 6A) and subcutaneously transplanted them into immunocompromised female mice as cell clusters without using a PET membrane (Figure 6B). Three days later, a significant increase in the serum testosterone level was observed (Figure 6C). Blood levels of its metabolites, DHT and estradiol, were also significantly increased in the transplantation group (Table S5; Figures 6D and 6E, respectively). Furthermore, we transplanted LLCs into immunocompromised castrated male mice using the same method (Figure 6F). Relative to the castrated group, which served as the negative control, the LLC transplantation group showed a significant increase in serum testosterone and DHT levels, with a tendency toward increased estradiol levels (Table S5; Figures 6G–6I). The blood testosterone levels were 0 ± 0 ng/mL in the castrated group, 0.04 ± 0.04 ng/mL in the LLC-transplanted group, and 0.83 ± 0.55 ng/mL in the non-castrated group. These findings indicated that LLCs, when subcutaneously engrafted into immunocompromised mice, are viable *in vivo* and secrete testosterone.

(E) Three days after transplantation, the substrate AkaLumine was injected intraperitoneally in mice; 10 min later, AkaBLI luminescence was detected subcutaneously in mice in the Venus-Akaluc-LLC live-transplantation group (group 1). Images of each group are shown. $n = 3$ independent experiments.
(F–H) The results of the blood hormone levels taken 3 days after transplantation are shown for both the group of mice in which LLCs were adhered to the PET membrane and then transplanted (group 2 in Figure 5D) and the control group that received only the membrane transplant (group 3). Statistical significance was denoted by **** for $p < 0.0001$. Data are presented as mean ± SD from independent experiments, with a sample size of $n = 10$ for group 2 and $n = 7$ for group 3.

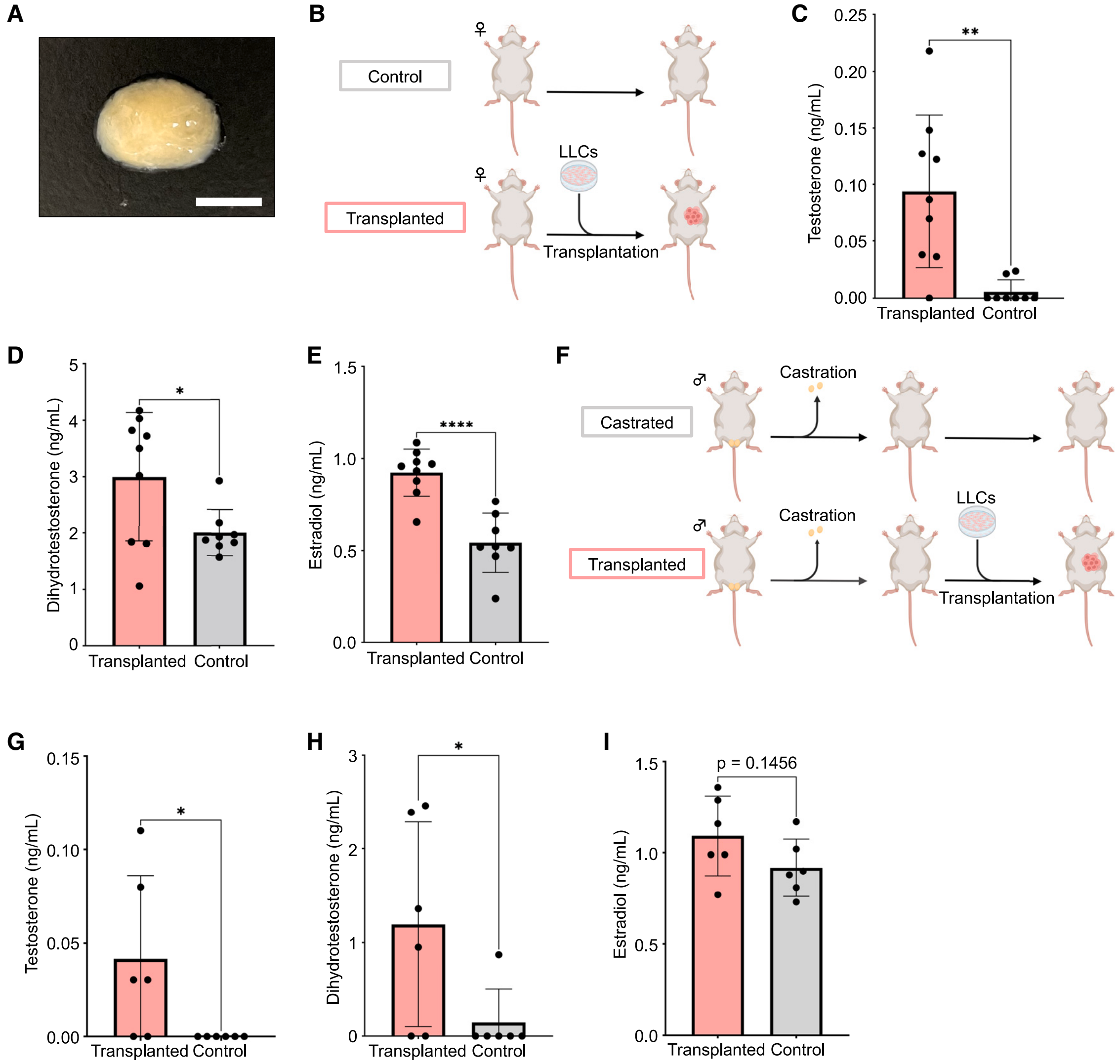


**Figure 6. Transplantation of LLCs as cell clusters into mice**

(A) LLC clusters were formed by removing the cells from the culture dish using a cell scraper. Scale bar: 5 mm.

(B) LLCs were transplanted subcutaneously into immunocompromised female mice as clusters. No cell transplantation was performed in the control mice.

(C–E) Serum testosterone, dihydrotestosterone, and estradiol levels were measured 3 days after cell transplantation. Statistical significance was denoted by **** for $p < 0.0001$, ** for $p < 0.01$, and * for $p < 0.05$. Data are presented as the mean ± SD from independent experiments, with sample sizes of $n = 9$ for the transplantation group and $n = 8$ for the control group.

(F) LLC clusters were transplanted subcutaneously into immunocompromised castrated male mice. No cell transplantation was performed in control mice.

(G–I) Four days after transplantation, the serum testosterone, dihydrotestosterone, and estradiol levels were measured. Statistical significance was denoted as * for $p < 0.05$. Data are presented as the mean ± SD from independent experiments, with sample sizes of $n = 6$ for each group.

## DISCUSSION

In this study, we successfully generated LLCs that produced testosterone at concentrations approximately 22 times higher than those achieved with our previous method (Ishida et al., 2021). The testosterone secretion was approximately 245 ng per million cells over 24 h, exceeding the highest reported value of approximately 70 ng for human LLCs derived from SLCs (Feng et al., 2021). These findings imply that LLCs generated with our current protocol exhibit exceptional testosterone secretion ability. Furthermore, our LLCs were able to be maintained *in vitro* for

more than 16–21 weeks while continuously secreting testosterone, whereas those in a previous report could only be maintained for a maximum of 7 weeks (Ishida et al., 2021; Li et al., 2022b). With the aim of achieving LLC transplantation therapy, LLCs capable of sustained secretion of large amounts of testosterone would be exceedingly useful, as they would not only reduce the number of cells needed but also extend the interval between transplants. Although significant improvements were made in comparison to conventional methods for generating LLCs, there is still room for further optimization regarding EB size and cytokine concentrations.

At present, the mechanism underlying this prolonged viability remains unclear, although the existence of a subset within our LLCs exhibiting molecular signatures akin to earlier-stage Leydig cells, such as FLCs and SLCs, as revealed by an scRNA-seq analysis, might hint at one of the mechanisms underlying their sustained long-term viability. A further investigation is warranted to determine whether or not LLCs can be maintained over the long term *in vivo*.

Although the differentiation efficiency of stem cells to LLCs was reported to peak at 50% (Chen et al., 2019), our improved method achieved a differentiation efficiency of 99% without the need for any purification process, which simplifies the manufacturing process for clinical application. Furthermore, the expression of the undifferentiated gene marker *LIN28* in the resultant cell population was below a certain threshold. While a negative *LIN28A*/ddPCR test result does not solely indicate safety, the absence of a certain threshold of undifferentiated cells is among the fundamental quality attributes crucial for ensuring safety, thus indicating a reduced risk of teratoma formation after transplantation (Knoepfler, 2009; Koyanagi-Aoi et al., 2013). In addition, our LLCs can be cryopreserved and retain their testosterone-secreting capacity after thawing, which may be advantageous for clinical application. Although the issue of potential contamination of undifferentiated cells and cryo-preservability have been significant concerns in the clinical applications of LLCs, prior reports on Leydig-like cells derived from pluripotent stem cells (Chen et al., 2019; Ishida et al., 2021; Li et al., 2019) have not addressed these issues. Consequently, whether or not our methodology surpasses the previously reported methods in this regard remains uncertain, but the current method still addresses these concerns.

In previous reports, the standard method for engrafting generated LLCs involved transplantation into the abdominal cavity or testicular interstitial tissue of mice or rats (Chen et al., 2019, Feng et al., 2021; Jiang et al., 2014). However, in terms of clinical application, these cell transplantation methods have issues related to invasiveness, procedural complexity, and challenges in cell removal when required. To overcome these issues, the subcutaneous transplantation of LLCs is ideal. SLCs were reportedly successfully extracted from 6-week-old mice and ectopically transplanted into the subcutaneous tissue, resulting in an increase in serum testosterone levels (Arora et al., 2019). However, the main issues with that approach were that the experiment was performed using mouse cells, not human cells, and that the SLCs were extracted from young animals and then transplanted back into the same animals. Furthermore, Sertoli and myoid cells were required for cellular engraftment, as Leydig cells alone were insufficient, and these cells had to be engrafted simultaneously. In contrast, in our study, subcutaneous transplantation of the cells was performed using human cells, successfully engrafting LLC cells alone without the support of Sertoli or myoid cells, which also resulted in increased serum testosterone levels. We believe that these results are significant in that they represent a successful transplantation method with consideration for the clinical application of LLC in patients with LOH syndrome.

Several limitations associated with the present study warrant mention. First, our improved method forcibly expressed *NR5A1* to generate LLCs, but stopping the forced expression of *NR5A1* rendered the LLCs unsustainable and incapable of secreting testosterone (Figure 2A). Although *NR5A1* has traditionally been considered essential for Leydig cell differentiation (Luo et al., 1994; Yang et al., 2015), it may also be a critical gene for maintaining the cells after differentiation completion. The Tet-Off system employed in this study can eliminate the need for the addition of doxycycline, but the expression of endogenous *NR5A1* would help promote enduring homeostasis in Leydig-like cells through mechanisms identical to those observed in authentic Leydig cells.

Second, the concentration of testosterone secreted by LLCs showed variability among the experimental replicates (Figures 1D and 2F). Although the cause of this variability is unclear, it is generally accepted that during the induction of differentiation using pluripotent stem cells, numerous cells undergo dynamic changes, leading to differences in the timing of differentiation among individual cells. Additionally, factors such as cell density, distance between cells, nutrient depletion, and waste accumulation vary among experiments and cannot be precisely controlled. Therefore, controlling this variability among experiments and producing a large quantity of stable quality LLCs remain a challenge to be addressed in future clinical applications.

Third, although we added gonadotropic hormones, such as LH and hCG, to the culture medium, the LLCs did not respond (Figures S6A and S6B) and continued to secrete

testosterone autonomously, regardless of the effects of such gonadotropic hormones. Therefore, it is desirable to develop LLCs that can physiologically control testosterone secretion and reproduce diurnal variations in the response to gonadotropic hormones. In this study, the expression of LHCGR was confirmed in more than 99% of LLCs by immunostaining and flow cytometry (Figures 4A–4D and S2C–S2F); however, without membrane permeabilization, the positive rate was only approximately 30% (Figures S6C and S6D). If LHCGR is stably expressed on the cell membrane surface, the responsiveness to gonadotropic hormones can be overcome. As LLCs did not respond to the addition of LH or hCG *in vitro*, we did not conduct experiments involving the addition of gonadotropic hormones after transplantation into mice. This issue remains a topic for future research.

Fourth, we have not yet determined whether our LLCs express HSD17B3 protein at levels comparable to those in authentic Leydig cells, as we were unable to isolate or culture primary testicular Leydig cells to be used as positive controls for immunostaining or flow cytometry. The mRNA expression level of *HSD17B3* was lower in LLCs than in testicular Leydig cells; however, it is known that in some cases, a protein's expression level can be high even when its mRNA expression is low (Kosti et al., 2016). Therefore, it remains unclear whether the HSD17B3 protein expression in LLCs is actually low. To determine whether LLCs have functions comparable to those of Leydig cells, it is essential to clarify whether the expression levels of *HSD17B3*, a marker for Leydig cells and an important enzyme in the final stage of testosterone biosynthesis, are sufficient.

Fifth, maintenance of LLCs requires the addition of forskolin. Because forskolin is a plant-derived substance, it is necessary to generate LLCs that do not require this substance.

Sixth, because the mice used for transplantation in this study were immunodeficient, it will be necessary to encapsulate LLCs in an immunoisolation device and engraft them into immunocompetent animals in future studies. Regarding the transplantation of endocrine cells, significant device development has occurred in the field of pancreatic beta cells (Shahjalal et al., 2018). The primary method for controlling rejection without immunosuppressive drugs involves encapsulating islets within a device made of biocompatible materials, including alginate, and transplanting them subcutaneously or intraperitoneally (An et al., 2018; Orive et al., 2015; Vegas et al., 2016). However, none of these approaches have yet been put to practical use. In our study, transplantation of LLCs as clusters significantly increased blood testosterone levels in mice. Embedding cell clusters in alginate may thus be a promising approach. Furthermore, to our knowledge, the method used in this study, whereby endocrine cells are attached to an artificial PET membrane and the resulting cell sheets are grown subcutaneously, is novel, and this approach is promising for clinical applications. By combining the findings from our cell transplantation experiments with the techniques and knowledge accumulated in relation to β-cell transplantation, it may be possible to determine the optimal method for LLC engraftment.

If these challenges can be overcome, regenerative medicine for LOH syndrome will become a reality.

## EXPERIMENTAL PROCEDURES

### Improved method for induction of differentiation into LLCs

Anti-adherence rinsing solution (STEMCELL Technologies, Cambridge, MA, USA) was added to AggreWell400 (6-well plates; STEMCELL Technologies) at a volume of 2 mL/well. The plates were then centrifuged at 1300×*g* for 5 min in a plate centrifuge, after which the rinsing solution was removed through aspiration. Confluent 3AB4-*NR5A1*-hiPSCs were detached from culture dishes using 0.5X TrypLE Select. A total of $1.4 \times 10^6$ viable cells were suspended in 5 mL of Step 1 medium (DMEM high glucose with 15% KSR, CHIR99021 [4 μM; Tocris Bioscience], BMP4 [80 ng/mL; R&D Systems, Minneapolis, MN, USA], VEGF [80 ng/mL; R&D Systems], CultureSure Y-27632 [10 μM], penicillin [50 units/mL], streptomycin [50 μg/mL], and doxycycline hyclate [1.5 μM]) and placed in a single well of rinsing solution-treated AggreWell400 plate. After centrifugation at 100×*g* for 3 min in a plate centrifuge, the cells were incubated at 37°C, 5% $CO_2$ for 6 days to form micro-EBs. The medium was not altered during this 6-day period. Micro-EBs were then collected in tubes and centrifuged at 100×*g* for 3 min. The supernatant was aspirated, and the micro-EBs were suspended in 4 mL of Step 2 medium (DMEM high glucose with 10% FBS, 8Br-cAMP [1 mM], forskolin [100 μM], penicillin [50 units/mL], streptomycin [50 μg/mL], and doxycycline hyclate [1.5 μM]) and distributed into 2 wells of a Nunc cell culture-treated multidish (6-well plate; Life Technologies). The cells were maintained at 37°C, 5% $CO_2$, and the medium was changed to Step 2 medium every 2–3 days. From day 17 of differentiation induction, the cells were sustained in Step 2 medium without 8Br-cAMP.

## RESOURCE AVAILABILITY

### Lead contact

Further information and requests for resources and reagents should be directed to and will be fulfilled by the lead contact, Takashi Aoi (takaaoi@med.kobe-u.ac.jp).

### Materials availability

This study did not generate new unique reagents.

### Data and code availability

The datasets generated during the current study are available in the Gene Expression Omnibus database.

(1) NGS Data: GSE244796 Go to https://www.ncbi.nlm.nih.gov/geo/query/acc.cgi?acc=GSE244796.

(2) scRNA-seq Data: GSE245553 Go to https://www.ncbi.nlm.nih.gov/geo/query/acc.cgi?acc=GSE245553.

The source code for the NGS and scRNA-seq analyses presented in this study is available at https://github.com/KatsuyaSato1212/Sato-et-al.–RStudio_code.git.

## ACKNOWLEDGMENTS

We thank all members of the laboratory for their scientific discussions, Yoko Matsuoka for administrative support, and Miwako Watanabe for technical support. We also thank Ryoko Hirohata and Michiko Nakamura for the plasmid construction. Naoya Hosono provided support for the scRNA-seq analysis. This work was supported by a grant from the Research Center Network for Realization of Regenerative Medicine (JP18bm0704005h0003 and JP21bm0404051h0003) (to T.A. and M.K.-A.) from the Japan Agency for Medical Research and Development (AMED), Akira Sakagami Fund for Research and Education, Kobe University Graduate School of Medicine (to T.A. and M.K.-A.), and JSPS KAKENHI (JP20K09578 [to M.F. and T.A.], JP23H03037 [to M.F., T.A., and K.S.], JP24K12483 [to K.S., M.F., and T.A.], and JP17K07256 [to K.W.]).

## AUTHOR CONTRIBUTIONS

K.S. designed and performed the experiments, analyzed the data, interpreted the results, and wrote the manuscript. M.K.-A. contributed to experimental design and manuscript writing. K.U. analyzed the scRNA-seq data. Y.Y. conducted the PCR experiments. M.S. quantified various hormone levels. S.L., A.R., and K.W. created the PB-TA-MCS (KW107) and PB-CAG-tTA-IRESyhg (KW1526). K.C., H.M., and M.F. contributed to experimental design. T.A. oversaw the experiment design, result interpretation, and manuscript writing.

## DECLARATION OF INTERESTS

We have patents related to this work.

Title: Method for preparing human pluripotent stem cell-derived Leydig-like cell, and human pluripotent stem cell-derived Leydig-like cell, patent number: 6979702(JP).

## SUPPLEMENTAL INFORMATION

Supplemental information can be found online at https://doi.org/10.1016/j.stemcr.2024.102392.

Received: April 5, 2024
Revised: December 12, 2024
Accepted: December 13, 2024
Published: January 16, 2025

## REFERENCES

An, D., Chiu, A., Flanders, J.A., Song, W., Shou, D., Lu, Y.-C., Grunnet, L.G., Winkel, L., Ingvorsen, C., Christophersen, N.S., et al. (2018). Designing a retrievable and scalable cell encapsulation device for potential treatment of type 1 diabetes. Proc. Natl. Acad. Sci. USA *115*, E263–E272. https://doi.org/10.1073/pnas.1708806115.

Arora, H., Zuttion, M.S.S.R., Nahar, B., Lamb, D., Hare, J.M., and Ramasamy, R. (2019). Subcutaneous Leydig Stem Cell Autograft: A Promising Strategy to Increase Serum Testosterone. Stem Cells Transl. Med. *8*, 58–65. https://doi.org/10.1002/sctm.18-0069.

Basaria, S. (2014). Male hypogonadism. Lancet *383*, 1250–1263. https://doi.org/10.1016/S0140-6736(13)61126-5.

Bhasin, S., Cunningham, G.R., Hayes, F.J., Matsumoto, A.M., Snyder, P.J., Swerdloff, R.S., and Montori, V.M.; Task Force, Endocrine Society (2010). Testosterone Therapy in Men with Androgen Deficiency Syndromes: An Endocrine Society Clinical Practice Guideline. J. Clin. Endocrinol. Metab. *95*, 2536–2559. https://doi.org/10.1210/jc.2009-2354.

Mendis-Handagama, S.M., and Ariyaratne, H.B. (2001). Differentiation of the Adult Leydig Cell Population in the Postnatal Testis1. Biol. Reprod. *65*, 660–671. https://doi.org/10.1095/biolreprod65.3.660.

Chen, H., Ge, R.S., and Zirkin, B.R. (2009). Leydig cells: From stem cells to aging. Mol. Cell. Endocrinol. *306*, 9–16. https://doi.org/10.1016/j.mce.2009.01.023.

Chen, X., Li, C., Chen, Y., Xi, H., Zhao, S., Ma, L., Xu, Z., Han, Z., Zhao, J., Ge, R., and Guo, X. (2019). Differentiation of human induced pluripotent stem cells into Leydig-like cells with molecular compounds. Cell Death Dis. *10*, 220. https://doi.org/10.1038/s41419-019-1461-0.

Chitiashvili, T., Dror, I., Kim, R., Hsu, F.M., Chaudhari, R., Pandolfi, E., Chen, D., Liebscher, S., Schenke-Layland, K., Plath, K., and Clark, A. (2020). Female human primordial germ cells display X-chromosome dosage compensation despite the absence of X-inactivation. Nat. Cell Biol. *22*, 1436–1446. https://doi.org/10.1038/s41556-020-00607-4.

Curley, M., Gonzalez, Z.N., Milne, L., Hadoke, P., Handel, I., Péault, B., and Smith, L.B. (2019). Human Adipose-derived Pericytes Display Steroidogenic Lineage Potential in Vitro and Influence Leydig Cell Regeneration in Vivo in Rats. Sci. Rep. *9*, 15037. https://doi.org/10.1038/s41598-019-50855-0.

Davidoff, M.S., Middendorff, R., Enikolopov, G., Riethmacher, D., Holstein, A.F., and Müller, D. (2004). Progenitor cells of the testosterone-producing Leydig cells revealed. J. Cell Biol. *167*, 935–944. https://doi.org/10.1083/jcb.200409107.

Feng, X., Xia, K., Ke, Q., Deng, R., Zhuang, J., Wan, Z., Luo, P., Wang, F., Zang, Z., Sun, X., et al. (2021). Transplantation of encapsulated human Leydig-like cells: A novel option for the treatment of testosterone deficiency. Mol. Cell. Endocrinol. *519*, 111039. https://doi.org/10.1016/j.mce.2020.111039.

Gossen, M., and Bujard, H. (1992). Tight control of gene expression in mammalian cells by tetracycline-responsive promoters. Proc. Natl. Acad. Sci. USA *89*, 5547–5551. https://doi.org/10.1073/pnas.89.12.5547.

Haider, S.G. (2004). Cell biology of Leydig cells in the testis. Int. Rev. Cytol. *233*, 181–241. https://doi.org/10.1016/s0074-7696(04)33005-6.

Handelsman, D.J., Yeap, B., Flicker, L., Martin, S., Wittert, G.A., and Ly, L.P. (2015). Age-specific population centiles for androgen status in men. Eur. J. Endocrinol. *173*, 809–817. https://doi.org/10.1530/EJE-15-0380.

Huang, H., Zou, X., Zhong, L., Hou, Y., Zhou, J., Zhang, Z., Xing, X., and Sun, J. (2019). CRISPR/dCas9-mediated activation of multiple endogenous target genes directly converts human foreskin fibroblasts into Leydig-like cells. J. Cell Mol. Med. *23*, 6072–6084. https://doi.org/10.1111/jcmm.14470.

Huhtaniemi, I. (2014). Late-onset hypogonadism: current concepts and controversies of pathogenesis, diagnosis and treatment. Asian J. Androl. *16*, 192–202. https://doi.org/10.4103/1008-682x.122336.

Inoue, M., Baba, T., Takahashi, F., Terao, M., Yanai, S., Shima, Y., Saito, D., Sugihara, K., Miura, T., Takada, S., et al. (2022). Tmsb10 triggers fetal Leydig differentiation by suppressing the RAS/ERK pathway. Commun. Biol. *5*, 974. https://doi.org/10.1038/s42003-022-03941-5.

Ishida, T., Koyanagi-Aoi, M., Yamamiya, D., Onishi, A., Sato, K., Uehara, K., Fujisawa, M., and Aoi, T. (2021). Differentiation of Human Induced Pluripotent Stem Cells Into Testosterone-Producing Leydig-like Cells. Endocrinology *162*, bqab202. https://doi.org/10.1210/endocr/bqab202.

Iwano, S., Sugiyama, M., Hama, H., Watakabe, A., Hasegawa, N., Kuchimaru, T., Tanaka, K.Z., Takahashi, M., Ishida, Y., Hata, J., et al. (2018). Single-cell bioluminescence imaging of deep tissue in freely moving animals. Science *359*, 935–939. https://doi.org/10.1126/science.aaq1067.

Ji, W., Chen, Y., Wang, L., Xu, Z., Ahmed, J., Ge, R., Chu, M., and Guo, X. (2020). Differentiation of human umbilical cord mesenchymal stem cells into Leydig-like cells with defined molecular compounds. Hum. Cell *33*, 318–329. https://doi.org/10.1007/s13577-020-00324-y.

Jiang, M.H., Cai, B., Tuo, Y., Wang, J., Zang, Z.J., Tu, X., Gao, Y., Su, Z., Li, W., Li, G., et al. (2014). Characterization of Nestin-positive stem Leydig cells as a potential source for the treatment of testicular Leydig cell dysfunction. Cell Res. *24*, 1466–1485. https://doi.org/10.1038/cr.2014.149.

Khera, M., Adaikan, G., Buvat, J., Carrier, S., El-Meliegy, A., Hatzimouratidis, K., McCullough, A., Morgentaler, A., Torres, L.O., and Salonia, A. (2016). Diagnosis and Treatment of Testosterone Deficiency: Recommendations from the Fourth International Consultation for Sexual Medicine (ICSM 2015). J. Sex. Med. *13*, 1787–1804. https://doi.org/10.1016/j.jsxm.2016.10.009.

Knoepfler, P.S. (2009). Deconstructing stem cell tumorigenicity: a roadmap to safe regenerative medicine. Stem Cell. *27*, 1050–1056. https://doi.org/10.1002/stem.37.

Kosti, I., Jain, N., Aran, D., Butte, A.J., and Sirota, M. (2016). Cross-tissue Analysis of Gene and Protein Expression in Normal and Cancer Tissues. Sci. Rep. *6*, 24799. https://doi.org/10.1038/srep24799.

Koyanagi-Aoi, M., Ohnuki, M., Takahashi, K., Okita, K., Noma, H., Sawamura, Y., Teramoto, I., Narita, M., Sato, Y., Ichisaka, T., et al. (2013). Differentiation-defective phenotypes revealed by large-scale analyses of human pluripotent stem cells. Proc. Natl. Acad. Sci. USA *110*, 20569–20574. https://doi.org/10.1073/pnas.1319061110.

Kuroda, T., Yasuda, S., Matsuyama, S., Tano, K., Kusakawa, S., Sawa, Y., Kawamata, S., and Sato, Y. (2015). Highly sensitive droplet digital PCR method for detection of residual undifferentiated cells in cardiomyocytes derived from human pluripotent stem cells. Regen. Ther. *2*, 17–23. https://doi.org/10.1016/j.reth.2015.08.001.

Li, L., Li, Y., Sottas, C., Culty, M., Fan, J., Hu, Y., Cheung, G., Chemes, H.E., and Papadopoulos, V. (2019). Directing differentiation of human induced pluripotent stem cells toward androgen-producing Leydig cells rather than adrenal cells. Proc. Natl. Acad. Sci. USA *116*, 23274–23283. https://doi.org/10.1073/pnas.1908207116.

Li, X., Tian, E., Wang, Y., Wen, Z., Lei, Z., Zhong, Y., and Ge, R.S. (2022a). Stem Leydig cells: Current research and future prospects of regenerative medicine of male reproductive health. Semin. Cell Dev. Biol. *121*, 63–70. https://doi.org/10.1016/j.semcdb.2021.05.007.

Li, Z.-H., Lu, J.-D., Li, S.-J., Chen, H.-L., and Su, Z.-J. (2022b). Generation of Leydig-like cells: approaches, characterization, and challenges. Asian J. Androl. *24*, 335–344. https://doi.org/10.4103/aja202193.

Luo, X., Ikeda, Y., and Parker, K.L. (1994). A cell-specific nuclear receptor is essential for adrenal and gonadal development and sexual differentiation. Cell *77*, 481–490.

Merchant-Larios, H., and Moreno-Mendoza, N. (1998). Mesonephric Stromal Cells Differentiate into Leydig Cells in the Mouse Fetal Testis. Exp. Cell Res. *244*, 230–238. https://doi.org/10.1006/excr.1998.4215.

Mularoni, V., Esposito, V., Di Persio, S., Vicini, E., Spadetta, G., Berloco, P., Fanelli, F., Mezzullo, M., Pagotto, U., Pelusi, C., et al. (2020). Age-related changes in human Leydig cell status. Hum. Reprod. *35*, 2663–2676. https://doi.org/10.1093/humrep/deaa271.

Nagai, T., Ibata, K., Park, E.S., Kubota, M., Mikoshiba, K., and Miyawaki, A. (2002). A variant of yellow fluorescent protein with fast and efficient maturation for cell-biological applications. Nat. Biotechnol. *20*, 87–90. https://doi.org/10.1038/nbt0102-87.

Odeh, H.M., Kleinguetl, C., Ge, R., Zirkin, B.R., and Chen, H. (2014). Regulation of the proliferation and differentiation of Leydig stem cells in the adult testis. Biol. Reprod. *90*, 123. https://doi.org/10.1095/biolreprod.114.117473.

Ohta, R., Niwa, A., Taniguchi, Y., Suzuki, N.M., Toga, J., Yagi, E., Saiki, N., Nishinaka-Arai, Y., Okada, C., Watanabe, A., et al. (2016). Laminin-guided highly efficient endothelial commitment from human pluripotent stem cells. Sci. Rep. *6*, 35680. https://doi.org/10.1038/srep35680.

Orive, G., Santos, E., Poncelet, D., Hernández, R.M., Pedraz, J.L., Wahlberg, L.U., De Vos, P., and Emerich, D. (2015). Cell encapsulation: technical and clinical advances. Trends Pharmacol. Sci. *36*, 537–546. https://doi.org/10.1016/j.tips.2015.05.003.

Shahjalal, H.M., Abdal Dayem, A., Lim, K.M., Jeon, T.-I., and Cho, S.-G. (2018). Generation of pancreatic β cells for treatment of diabetes: advances and challenges. Stem Cell Res. Ther. *9*, 355. https://doi.org/10.1186/s13287-018-1099-3.

Shin, E.-Y., Park, S., Choi, W.Y., and Lee, D.R. (2021). Rapid Differentiation of Human Embryonic Stem Cells into Testosterone-Producing Leydig Cell-Like Cells In vitro. Tissue Eng. Regen. Med. *18*, 651–662. https://doi.org/10.1007/s13770-021-00359-8.

Shin, Y.S., and Park, J.K. (2019). The Optimal Indication for Testosterone Replacement Therapy in Late Onset Hypogonadism. J. Clin. Med. *8*, 209. https://doi.org/10.3390/jcm8020209.

Stellato, R.K., Feldman, H.A., Hamdy, O., Horton, E.S., and McKinlay, J.B. (2000). Testosterone, sex hormone-binding globulin, and the development of type 2 diabetes in middle-aged men: prospective results from the Massachusetts male aging study. Diabetes Care *23*, 490–494. https://doi.org/10.2337/diacare.23.4.490.

Takahashi, K., and Yamanaka, S. (2006). Induction of pluripotent stem cells from mouse embryonic and adult fibroblast cultures by defined factors. Cell *126*, 663–676. https://doi.org/10.1016/j.cell.2006.07.024.

Tsujimura, A., Miyagawa, Y., Takezawa, K., Okuda, H., Fukuhara, S., Kiuchi, H., Takao, T., Yamamoto, R., Nishida, M., Yamauchi-Takihara, K., et al. (2013). Is low testosterone concentration a risk factor for metabolic syndrome in healthy middle-aged men? Urology *82*, 814–819. https://doi.org/10.1016/j.urology.2013.06.023.

Vegas, A.J., Veiseh, O., Gürtler, M., Millman, J.R., Pagliuca, F.W., Bader, A.R., Doloff, J.C., Li, J., Chen, M., Olejnik, K., et al. (2016). Long-term glycemic control using polymer-encapsulated human stem cell–derived beta cells in immune-competent mice. Nat. Med. *22*, 306–311. https://doi.org/10.1038/nm.4030.

Wu, F.C.W., Tajar, A., Pye, S.R., Silman, A.J., Finn, J.D., O'Neill, T.W., Bartfai, G., Casanueva, F., Forti, G., Giwercman, A., et al. (2008). Hypothalamic-pituitary-testicular axis disruptions in older men are differentially linked to age and modifiable risk factors: the European Male Aging Study. J. Clin. Endocrinol. Metab. *93*, 2737–2745. https://doi.org/10.1210/jc.2007-1972.

Yang, Y., Su, Z., Xu, W., Luo, J., Liang, R., Xiang, Q., Zhang, Q., Ge, R.-S., and Huang, Y. (2015). Directed Mouse Embryonic Stem Cells into Leydig-Like Cells Rescue Testosterone-Deficient Male Rats In Vivo. Stem Cell. Dev. *24*, 459–470. https://doi.org/10.1089/scd.2014.0370.

Yang, Y., Zhou, C., Zhang, T., Li, Q., Mei, J., Liang, J., Li, Z., Li, H., Xiang, Q., Zhang, Q., et al. (2020). Conversion of Fibroblast into Functional Leydig-like Cell Using Defined Small Molecules. Stem Cell Rep. *15*, 408–423. https://doi.org/10.1016/j.stemcr.2020.07.002.

Yao, S., Wei, X., Deng, W., Wang, B., Cai, J., Huang, Y., Lai, X., Qiu, Y., Wang, Y., Guan, Y., and Wang, J. (2022). Nestin-dependent mitochondria-ER contacts define stem Leydig cell differentiation to attenuate male reproductive ageing. Nat. Commun. *13*, 4020. https://doi.org/10.1038/s41467-022-31755-w.

Yu, J., Vodyanik, M.A., Smuga-Otto, K., Antosiewicz-Bourget, J., Frane, J.L., Tian, S., Nie, J., Jonsdottir, G.A., Ruotti, V., Stewart, R., et al. (2007). Induced Pluripotent Stem Cell Lines Derived from Human Somatic Cells. Science *318*, 1917–1920. https://doi.org/10.1126/science.1151526.

**Stem Cell Reports, Volume *20***

## Supplemental Information

# Efficient differentiation of human iPSCs into Leydig-like cells capable of long-term stable secretion of testosterone

**Katsuya Sato, Michiyo Koyanagi-Aoi, Keiichiro Uehara, Yosuke Yamashita, Masakazu Shinohara, Suji Lee, Anika Reinhardt, Knut Woltjen, Koji Chiba, Hideaki Miyake, Masato Fujisawa, and Takashi Aoi**

Figure S1

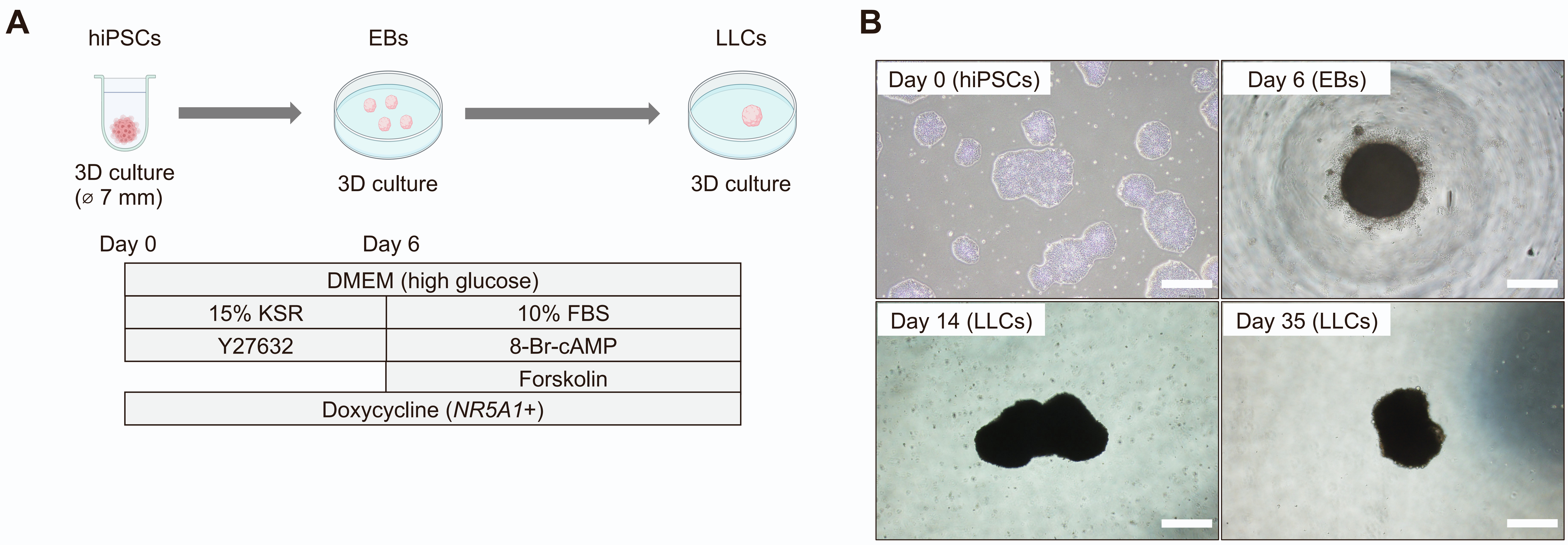
A
hiPSCs
EBs
LLCs
3D culture
(⌀ 7 mm)
3D culture
3D culture
Day 0
Day 6
DMEM (high glucose)
15% KSR
10% FBS
Y27632
8-Br-cAMP
Forskolin
Doxycycline (NR5A1+)
B
Day 0 (hiPSCs)
Day 6 (EBs)
Day 14 (LLCs)
Day 35 (LLCs)

*Figure S2*

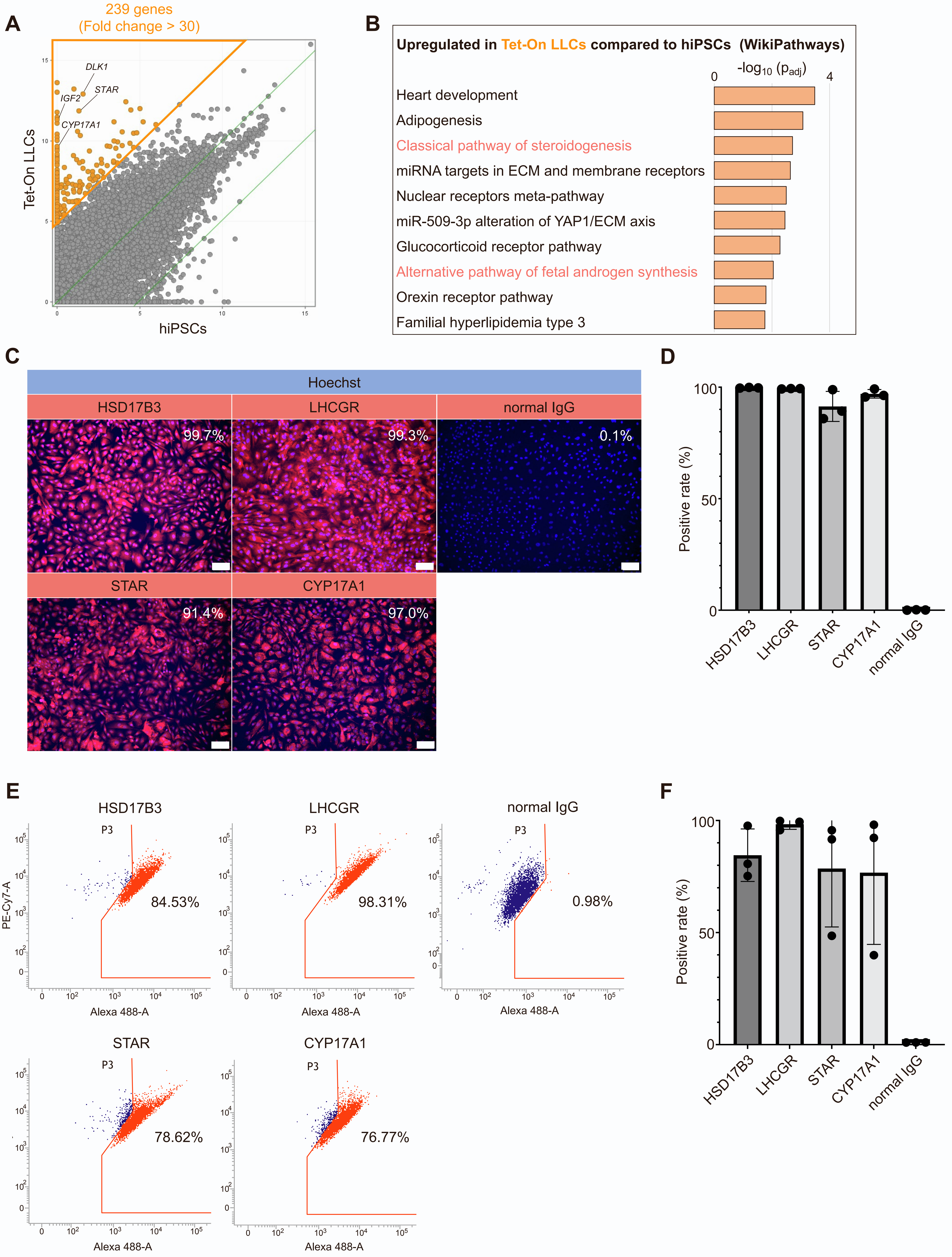
A
239 genes
(Fold change > 30)
DLK1
STAR
IGF2
CYP17A1
Tet-On LLCs
hiPSCs
B
Upregulated in Tet-On LLCs compared to hiPSCs (WikiPathways)
-log10 (padj)
Heart development
Adipogenesis
Classical pathway of steroidogenesis
miRNA targets in ECM and membrane receptors
Nuclear receptors meta-pathway
miR-509-3p alteration of YAP1/ECM axis
Glucocorticoid receptor pathway
Alternative pathway of fetal androgen synthesis
Orexin receptor pathway
Familial hyperlipidemia type 3
C
Hoechst
HSD17B3
LHCGR
normal IgG
99.7%
99.3%
0.1%
STAR
CYP17A1
91.4%
97.0%
D
Positive rate (%)
HSD17B3
LHCGR
STAR
CYP17A1
normal IgG
E
HSD17B3
LHCGR
normal IgG
P3
84.53%
98.31%
0.98%
PE-Cy7-A
Alexa 488-A
STAR
CYP17A1
78.62%
76.77%
F
Positive rate (%)
HSD17B3
LHCGR
STAR
CYP17A1
normal IgG

***Figure S3***

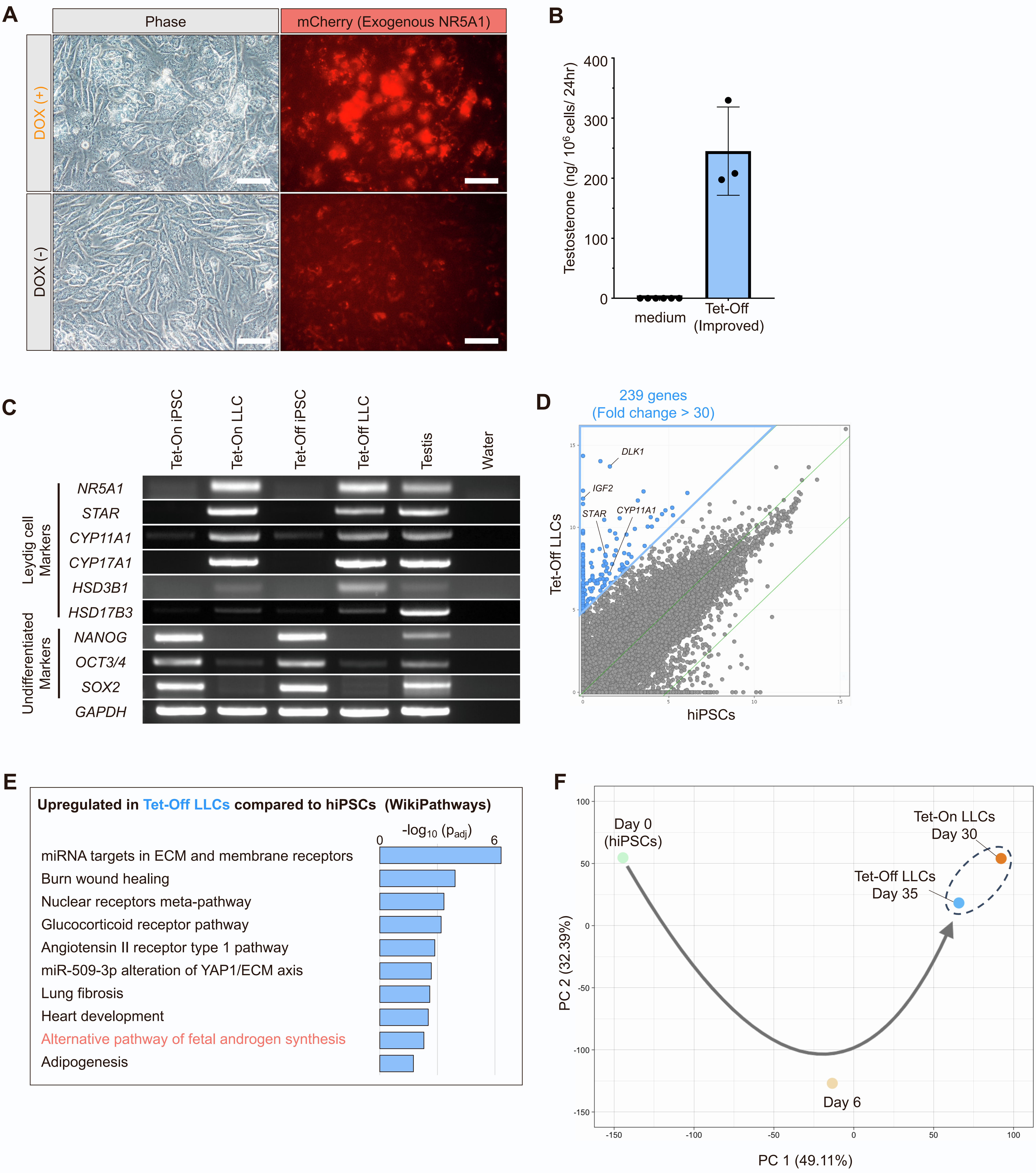
A
Phase
mCherry (Exogenous NR5A1)
DOX (+)
DOX (-)
B
Testosterone (ng/ 10^6 cells/ 24hr)
400
300
200
100
0
medium
Tet-Off (Improved)
C
Tet-On iPSC
Tet-On LLC
Tet-Off iPSC
Tet-Off LLC
Testis
Water
Leydig cell Markers
NR5A1
STAR
CYP11A1
CYP17A1
HSD3B1
HSD17B3
Undifferentiated Markers
NANOG
OCT3/4
SOX2
GAPDH
D
239 genes
(Fold change > 30)
DLK1
IGF2
STAR
CYP11A1
Tet-Off LLCs
hiPSCs
E
Upregulated in Tet-Off LLCs compared to hiPSCs (WikiPathways)
-log10 (padj)
0
6
miRNA targets in ECM and membrane receptors
Burn wound healing
Nuclear receptors meta-pathway
Glucocorticoid receptor pathway
Angiotensin II receptor type 1 pathway
miR-509-3p alteration of YAP1/ECM axis
Lung fibrosis
Heart development
Alternative pathway of fetal androgen synthesis
Adipogenesis
F
Day 0
(hiPSCs)
Tet-On LLCs
Day 30
Tet-Off LLCs
Day 35
Day 6
PC 2 (32.39%)
PC 1 (49.11%)
100
50
0
-50
-100
-150
-150
-100
-50
0
50
100

**Figure S4**

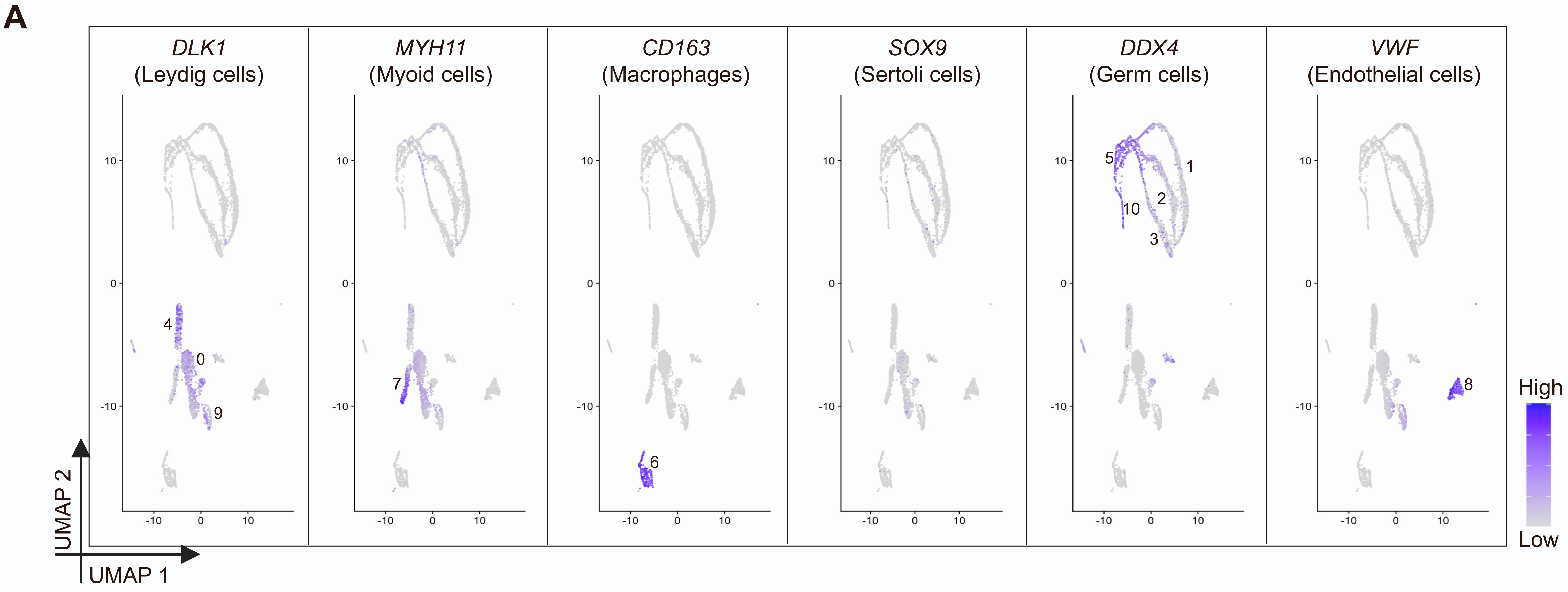


B

| Subset 1 > Subset 2 | Subset 2 > Subset 1 |
|---|---|
| *TEX30* | *MMRN1* |
| *PTRH1* | *CXCL3* |
| *NRK* | *CCL21* |
| *HSPA12A* | *CXCL2* |
| *PCDH18* | *FLI1* |
| *KANK2* | *NES* |
| *MMP23B* | *PDLIM1* |
| *MIR202HG* | *WFDC2* |
| *HSPA5* | *LAPTM5* |
| *CYP11A1* | *SORBS2* |

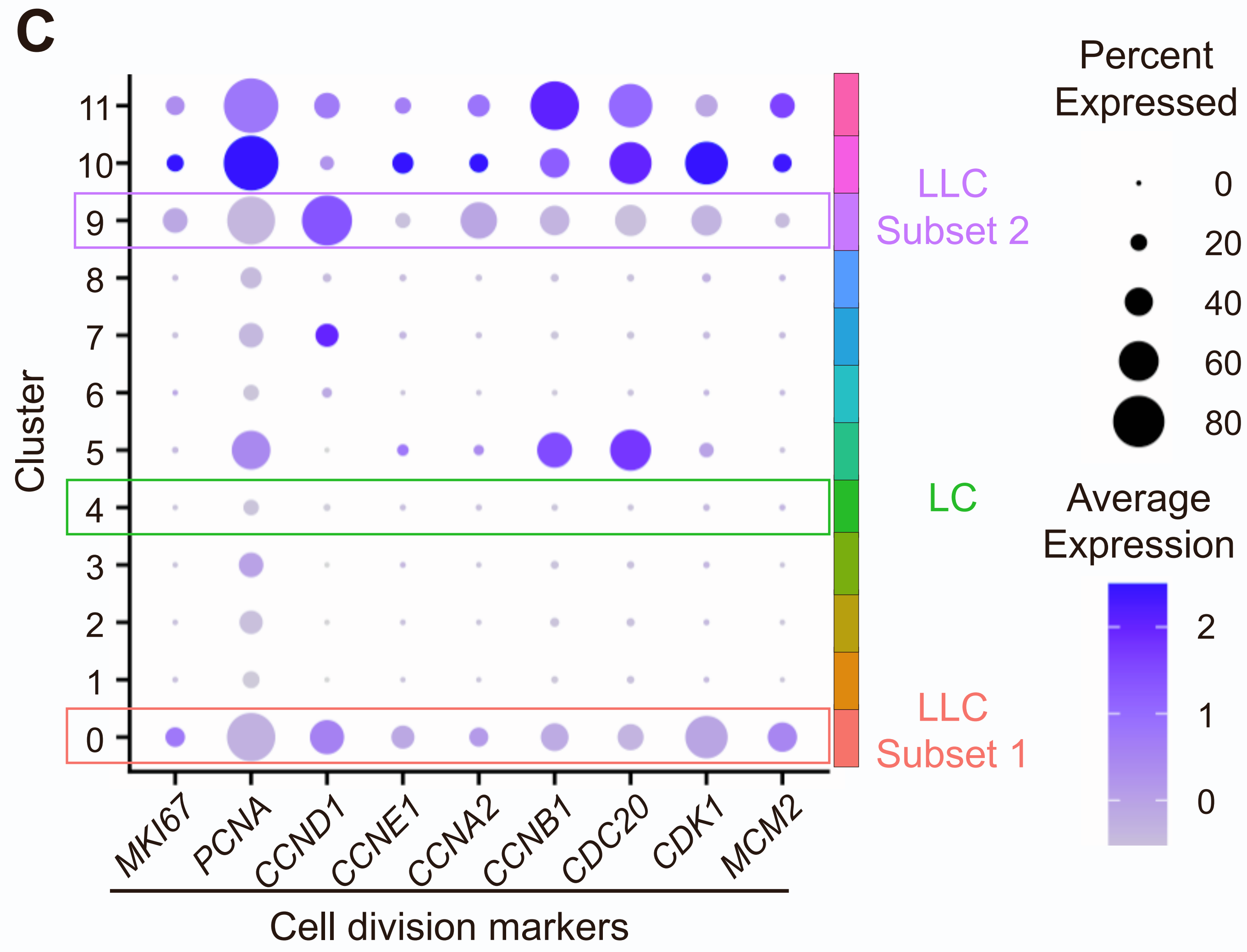


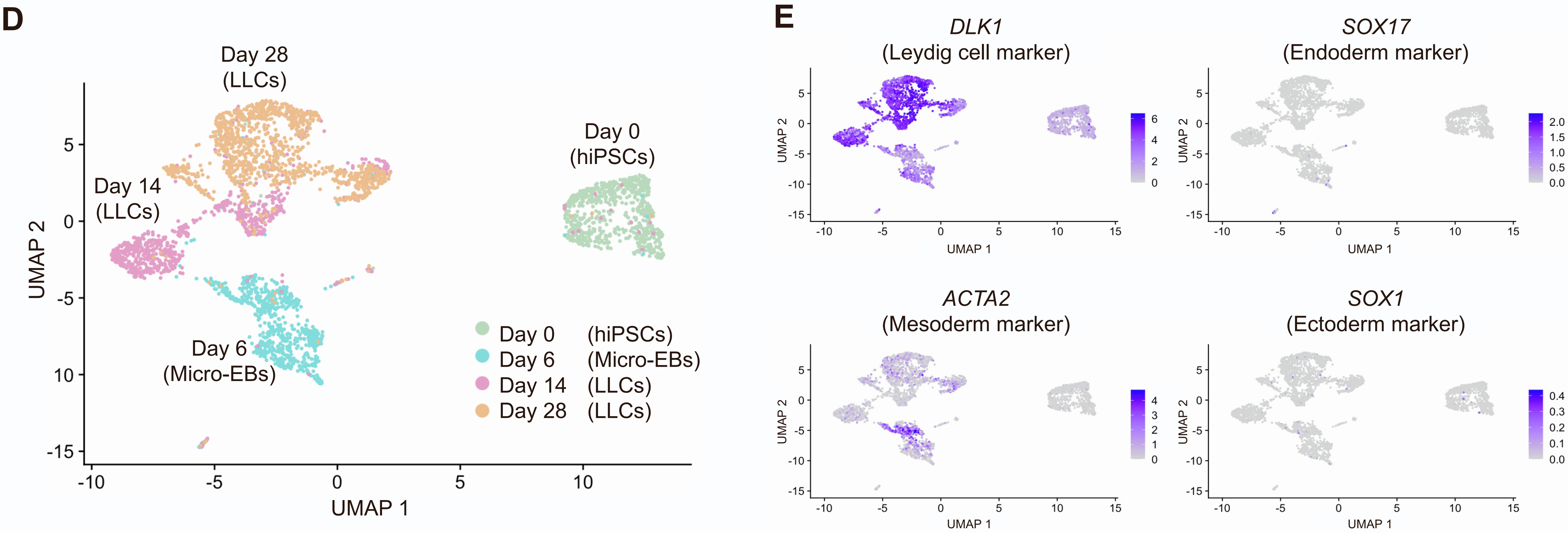

Figure S5

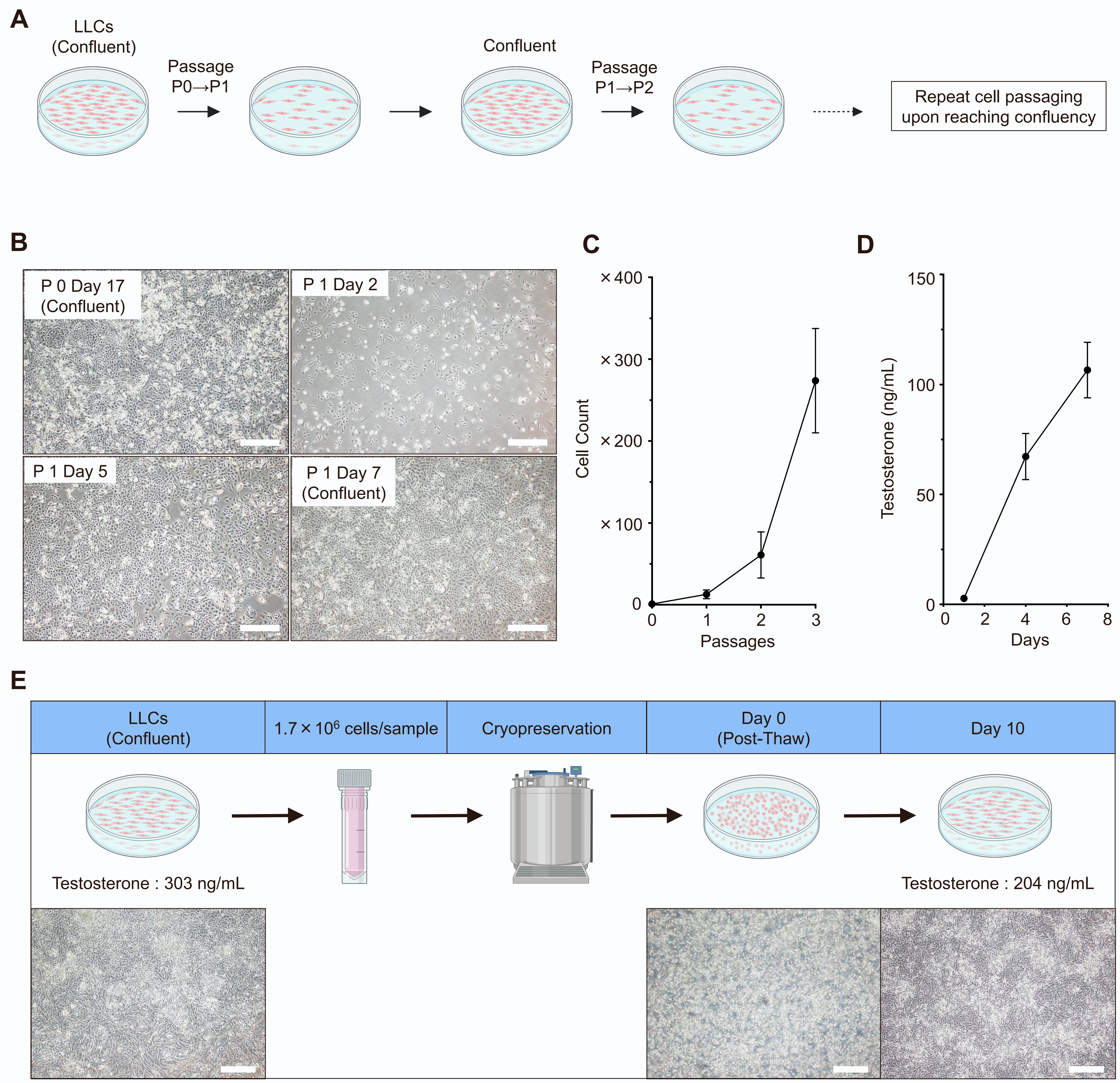

A
LLCs
(Confluent)
Passage
P0→P1
Confluent
Passage
P1→P2
Repeat cell passaging
upon reaching confluency
B
P 0 Day 17
(Confluent)
P 1 Day 2
P 1 Day 5
P 1 Day 7
(Confluent)
C
Cell Count
× 400
× 300
× 200
× 100
0
0
1
2
3
Passages
D
Testosterone (ng/mL)
150
100
50
0
0
2
4
6
8
Days
E
LLCs
(Confluent)
1.7 × 10⁶ cells/sample
Cryopreservation
Day 0
(Post-Thaw)
Day 10
Testosterone : 303 ng/mL
Testosterone : 204 ng/mL

*Figure S6*

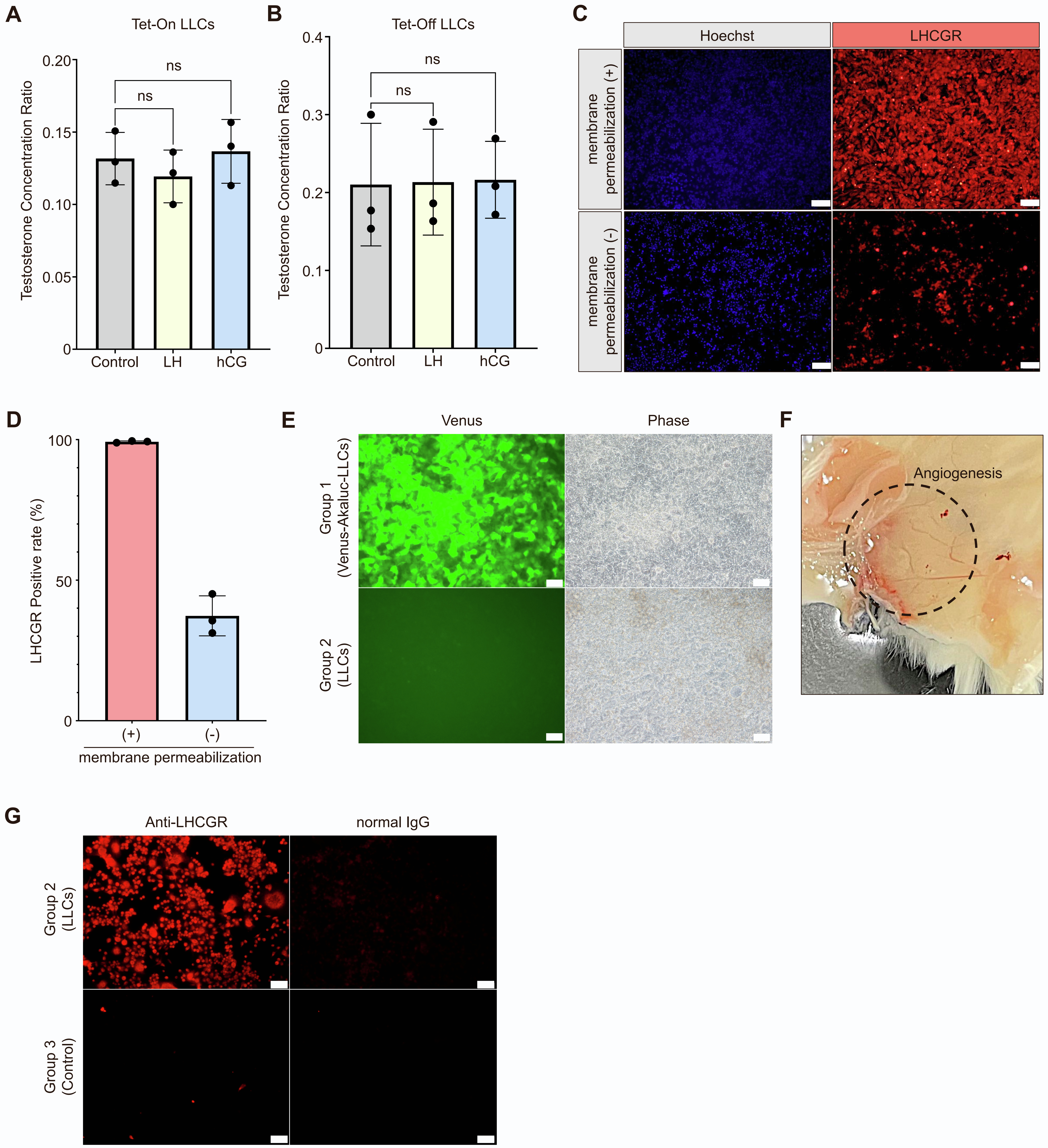

A
Tet-On LLCs
Testosterone Concentration Ratio
0.20
0.15
0.10
0.05
0
ns
ns
Control
LH
hCG
B
Tet-Off LLCs
Testosterone Concentration Ratio
0.4
0.3
0.2
0.1
0
ns
ns
Control
LH
hCG
C
Hoechst
LHCGR
membrane permeabilization (+)
membrane permeabilization (-)
D
LHCGR Positive rate (%)
100
50
0
(+)
(-)
membrane permeabilization
E
Venus
Phase
Group 1 (Venus-Akaluc-LLCs)
Group 2 (LLCs)
F
Angiogenesis
G
Anti-LHCGR
normal IgG
Group 2 (LLCs)
Group 3 (Control)

## SUPPLEMENTARY FIGURE LEGENDS

### Figure S1. Conventional method of generating hiPSC-derived LLCs

A) Schematic representation of the conventional differentiation protocol for generating LLCs from hiPSCs. This method involves the forced expression of *NR5A1* in hiPSCs, which are then maintained in 3D culture.

B) Morphological changes in cells generated by the conventional method, as observed by phase contrast microscopy on days 0 (hiPSCs), 6 (embryoid bodies), 14, and 35 (LLCs). Scale bars: 500 μm.

### Figure S2. The evaluation of Tet-On LLCs using NGS, immunostaining, and flow cytometry

A) The gene expression of a comprehensive set of 53,827 genes was examined using next-generation sequencing (NGS). Among the examined genes, 239 showed a substantial 30-fold increase in expression on day 14 (LLCs) compared with day 0 (hiPSCs).

B) A pathway analysis was conducted on the 239 genes extracted using the methods described above, leveraging the WikiPathways database, and the top 10 pathways were subsequently identified.

C) Leydig cell marker levels were assessed using an immunofluorescence assay. The percentages presented in the figure are the average positivity rates from three independent experiments, which assessed LLCs at days 28-35 of culture. Scale bars: 100 μm.

D) Statistical analyses of the immunofluorescence assay. Positivity was quantitatively evaluated using the ImageJ software program. Mean value ± SD (n = 3 independent experiments).

E) Quantification of the Leydig cell markers was performed using flow cytometry. The LLCs were analyzed on days 27-48 of cell culture. A two-dimensional analysis was conducted to ascertain the positivity rate.

F) Statistical analyses of flow cytometry. Mean value ± SD (n = 3 independent experiments).

**Figure S3. The evaluation of Tet-Off LLCs**

A) The removal of doxycycline from Tet-On LLCs medium resulted in a decrease in the red fluorescent protein expression of mCherry. This suggested that the expression of exogenous *NR5A1* ceased. Scale bars: 50 µm.

B) Testosterone concentration in the culture supernatant of Tet-Off LLCs was measured. Cells were detached from the culture dish using 0.5X TrypLE Select, and the cell count was measured to calculate the amount of testosterone secreted per million cells in 24 h. The findings are expressed as the mean ± SD from independent experiments, with sample sizes of n = 6 for the control (medium) group, n = 3 for the Tet-Off LLCs group.

C) Semi-quantitative RT-PCR showed that Tet-On LLC and Tet-Off LLC cells express steroid hormone metabolism genes (*STAR*, *CYP11A1*, *CYP17A1*, *HSD3B1*, and *HSD17B3*). The testis was used as a positive control for the marker genes.

D) A comprehensive set of 53,827 genes was analyzed for gene expression using NGS. Among the examined genes, 239 showed a 30-fold increase in expression in Tet-Off LLCs on day 35 compared to hiPSCs.

E) A WikiPathway analysis was conducted on the 239 identified genes using the methods described above. The top 10 pathways were identified.

F) A principal component analysis of NGS data from day 0 (hiPSCs), Tet-On day 6 (micro-EBs), Tet-On LLCs (day 30), and Tet-Off LLCs (day 35). The horizontal axis represents the score of principal component 1, and the vertical axis represents the score of principal component 2.

**Figure S4. Characterization of gene expression changes during the induction of LLC differentiation using scRNA-seq**

A) Cluster identification was performed using markers specific to each cell type.

B) The top 10 genes upregulated in each subset are listed when comparing LLC subsets 1 and 2 (See also Table S5).

C) The expression of the genes associated with cell division was compared among the identified clusters and is represented using dotplots.

D) UMAP plots were generated using cell sample data collected at various time points during the Tet-On LLC differentiation induction process (days 0, 6, 14, and 28).

E) UMAP plots indicate changes in the expression of DLK1 (a Leydig cell marker), SOX17 (an endoderm marker), ACTA2 (a mesoderm marker), and SOX1 (an ectoderm marker) on days 0, 6, 14, and 28 after differentiation induction.

**Figure S5. LLCs can be proliferated by passaging and can also be cryopreserved and thawed**

A) Once the LLCs reached confluence, they were passaged at 1/6 cell density in new wells. This was repeated each time the cells reached confluence.

B) On day 17 of differentiation induction, LLCs that had reached confluence were passaged into another well, and images were captured using phase-contrast microscopy on days 2, 5, and 7 post-passaging. Scale bars: 500 µm.

C) Proliferation curves for repeated passages of LLCs. The proliferation rate was evaluated in triplicate, and a curve was plotted. The vertical axis represents the cell count, with the cell count on P0 day 17 set at 1. Mean ± SD, n = 3 independent experiments.

D) Changes in testosterone concentration in culture supernatant after the first passaging. The data represent the mean ± SD of three independent experiments, each performed in triplicate.

E) LLCs that reached confluence were pipetted into tubes at a density of $1.7\times10^{6}$ cells/vial, frozen in liquid nitrogen, thawed, and subsequently cultured again. After thawing, LLCs secreted large amounts of testosterone into the culture supernatant. LLCs were imaged using phase-contrast microscopy pre-cryopreservation, immediately post-thaw (day 0), and on day 10 post-thaw. Scale bars: 500 µm.

**Figure S6. Response of LLCs to gonadotropic hormones and LLCs used for transplantation**

A-B) The vertical axis represents the ratio of the testosterone concentration in the culture supernatant after 3 h of incubation with LH (5 ng/mL) or hCG (1 U/mL)

relative to the baseline testosterone concentration measured during the preceding 24 h (from -24 to 0 h) in Tet-On LLCs (A) and Tet-Off LLCs (B). The values represent the mean ± SD (n = 3 independent experiments).

C) To investigate the localization of LHCGR on the cell surface, we performed immunostaining for LHCGR in Tet-Off LLCs with and without membrane permeabilization (upper and lower panels, respectively). Scale bars: 100 µm.

D) The percentage of LHCGR-positive cells was quantitatively evaluated using ImageJ . The values represent the mean ± SD (n = 3 independent experiments).

E) LLCs transfected with Venus-Akaluc (Group 1) displayed green Venus fluorescence, even after being cultured on PET membranes. Scale bars: 50 µm.

F) The subcutaneous tissue from the mice showed the development of angiogenic vessels in the area, consistent with the implantation of PET membranes.

G) Immunostaining of PET membranes harvested from mice indicated the presence of cells expressing the Leydig cell-specific marker LHCGR on the membranes. Scale bars: 50 µm.

**Video S1. Differentiation of mesodermal embryoid bodies into LLCs.** Micro-EBs transitioned from a 3D culture to an adherent culture to induce differentiation into LLCs on day 6. Images were captured every 5 min using a time-lapse phase-contrast microscope. Scale bar: 200 µm.

## KEY RESOURCES TABLE

| Reagent or Resource | SOURCE | IDENTIFIER |
|---|---|---|
| **Antibodies** | | |
| Rabbit anti-HSD17B3 | Genetex | Cat# GTX114480 |
| Rabbit anti-LHCGR | Genetex | Cat# GTX100008 |
| Rabbit anti-StAR | Genetex | Cat# GTX105716 |
| Rabbit anti-CYP17A1 | Genetex | Cat# GTX56294 |
| Normal Rabbit IgG | FUJIFILM Wako Pure Chemical Corporation | Cat# 148-09551 |
| donkey anti-rabbit IgG, Alexa Fluor 488 | Thermo Fisher Scientific | Cat# A-21206 |
| donkey anti-rabbit IgG, Alexa Fluor 594 | Thermo Fisher Scientific | Cat# A-21207 |
| Hoechst 33342, trihydrochloride | Thermo Fisher Scientific | Cat# H3570 |
| **Chemicals, peptides, and recombinant proteins** | | |
| StemFit AK02N | Ajinomoto | Cat# AJ100 |
| Penicillin-Streptomycin | Life Technologies | Cat# 15140-122 |
| 1X TrypLE Select | Thermo Fisher Scientific | Cat# A12859-01 |
| 0.5 mol/l-EDTA Solution | Nacalai Tesque | Cat# 06894-14 |
| iMatrix-511 silk | Nippi | Cat# 892021 |
| CultureSure Y-27632 | FUJIFILM Wako Pure Chemical Corporation | Cat# 034-24023 |
| DMEM High Glucose | Nacalai Tesque | Cat# 08459-64 |
| KnockOut SR | Life Technologies | Cat# 10828-010 |
| Doxycycline hyclate | Tocris Bioscience | Cat# 4090 |
| Fetal Bovine Serum (FBS) | Biowest | Cat# S1650 |
| 8-Bromoadenosine-3',5'-cyclic Monophosphate Sodium Salt Hydrate | Nacalai Tesque | Cat# 05450-86 |
| Forskolin | Abcam | Cat# ab120058 |
| CHIR99021 | TOCRIS | Cat# 252917-06-9 |
| BMP4 | R&D Systems | Cat# 314-BP-050 |
| VEGF | R&D Systems | Cat# 293-VE-050 |
| TRIzol | Life Technologies | Cat# 15596018 |
| 4% paraformaldehyde phosphate buffer solution | FUJIFILM Wako Pure Chemical Corporation | Cat# 163-20145 |

| | | |
|---|---|---|
| Blocking One | Nacalai Tesque | Cat# 03953-95 |
| Tween 20 | Nacalai Tesque | Cat# 28353-85 |
| Triton X-100 | Nacalai Tesque | Cat# 35501-15 |
| Luteinizing Hormone from human pituitary | Sigma-Aldrich | Cat# L6420-10UG |
| Gonadotropin for Injection, 5000 Units | ASKA Pharmaceutical Co., Ltd. | Cat# 123-00080-2 |
| LR Clonase™ II Plus | ThermoFisher Scientific | Cat# 12538200 |
| OPTI-MEM (1X) | Thermo Fisher Scientific | Cat# 31985-062 |
| FuGENE HD | Roche | Cat# E2311 |
| Hygromycin B | FUJIFILM Wako Pure Chemical Corporation | Cat# 084-07681 |
| Trypan Blue Stain (0.4%) | Life Technologies | Cat# 15250-061 |
| STEM-CELLBANKER | ZENOAQ | Cat# CB047 |
| AkaLumine-HCl | FUJIFILM Wako Pure Chemical Corporation | Cat# 018-26703 |
| **Critical commercial assays** | | |
| TURBO DNA-free kit | Life Technologies | Cat# AM1907 |
| SMARTer stranded total RNA-seq kit | Takara Bio | Cat# 634836 |
| One-Step RT-ddPCR Advanced Kit for Probes | Bio-Rad | Cat# 1864021 |
| PrimeScript II 1st Strand Synthesis Kit | Takara | Cat# 6210A |
| **Experimental models: Organisms/strains** | | |
| PB-TAC-ERN-NR5A1 | Ishida et al., 2021[16] | N/A |
| PB-TA-MCS | Knut Woltjen | N/A |
| PB-CAG-tTA-IREShyg | Knut Woltjen | N/A |
| pcDNA3 Venus-Akaluc | RIKEN | Cat# RDB15781 |
| pMXs retrovirus vector (pMXs-hOCT3/4) | addgene | Cat #17217 |
| NSG mice (NOD.Cg-Prkdcscid Il2rgtm1Wjl/SzJ) | The Jackson Laboratory Japan | N/A |
| Total RNA - Human Adult Normal Tissue: Testis | BioChain | Cat# R1234260-50 |
| **Deposited data** | | |
| NGS data | This paper | GEO: GSE244796 |

| | | |
|---|---|---|
| Single cell RNA-seq for adult human testes | Guo et al., 2018 | GEO: GSE112013 |
| Single cell RNA-seq for Leydig-like cells | This paper | GEO: GSE245553 |
| **Software and algorithms** | | |
| Strand NGS software | Strand Life Sciences | N/A |
| R | N/A | v4.1.2 |
| g:Profiler | Uku Raudvere et al., 2019[50] | https://biit.cs.ut.ee/gprofiler/gost |
| Seurat | Butler et al., 2019[51] | N/A |
| ImageJ Fiji software | National Institutes of Health | https://imagej.net/Fiji |
| QuantaSoft software | Bio-Rad | N/A |
| Living Image software | PerkinElmer | v4.3 |
| The Human Protein Atlas website | N/A | https://www.proteinatlas.org/ |
| **Culture Dish** | | |
| PrimeSurface96M | Sumitomo Bakelite | Cat# MS-9096M |
| PrimeSurface24F | Sumitomo Bakelite | Cat# MS-90240 |
| AggreWell400 (6-well plate) | STEMCELL Technologies | Cat# 34421 |
| Anti-Adherence Rinsing Solution | STEMCELL Technologies | Cat# 07010 |
| Nunc Cell-Culture Treated Multidish (6-well plate) | Life Technologies | Cat# 140675 |
| Nunc Cell-Culture Treated Multidish (24-well plate) | Life Technologies | Cat# 142475 |
| 0.4-µm pore size PET-Track-attached-membrane-12-well format | Corning | Cat# 353180 |
| 12-well TC-treated Polystyrene Permeable Support Companion Plate | Corning | Cat# 353503 |
| **Other** | | |
| BioStudio | Nikon | N/A |
| inverted fluorescence microscope | Olympus Corporation | N/A |
| BD Rhapsody Express Single-Cell Analysis System | BD Biosciences | N/A |
| FACSVerse instrument | BD Biosciences | N/A |
| QX200 Droplet Generator | Bio-Rad | N/A |
| C1000 Touch Thermal Cycler | Bio-Rad | N/A |

| | | |
|---|---|---|
| QX200 Droplet Reader | Bio-Rad | N/A |
| IVIS Lumina LT imaging system | PerkinElmer | N/A |
| Countess3 Automated Cell Counter | Invitrogen | Cat# AMQAX2000 |

## SUPPLEMENTAL EXPERIMENTAL PROCEDURE

### *hiPSC culture*

In this study, two types of hiPSCs (3AB4_NR5A1_hiPSC (Ishida et al., 2021) and 121-3 (Murai et al., 2023)) were used. This study was approved by the Ethics Committee of Kobe University Graduate School of Medicine (No. 1722), and informed consent was obtained from all donors. 3AB4 iPSC line and LLCs generated from this line were used in the experiments in Figures 1B-G, 2A, 2E-H, 3B-F, 4G-H, S1B, S2A-F, S3A, S3C, S4A-E and Video S1. On the other hand, 121-3 iPSC line and LLCs generated from this line were used in Figures 2D-H, 3B, 4A-E, 4G-H, 5B-H, 6A-I, S3B-F, S5B-E and S6A-G.

The hiPSCs were cultured using a previously described method (Nakagawa et al., 2014) with some modifications. In brief, hiPSCs were cultured in StemFit AK02N (Ajinomoto, Tokyo, Japan) supplemented with penicillin (50 units/mL) and streptomycin (50 µg/mL) (Life Technologies, Carlsbad, CA, USA) and maintained at 37°C, 5% $CO_2$. The culture medium was changed every other day and passaged with 0.5X TrypLE Select (1X TrypLE Select [Thermo Fisher Scientific, Waltham, MA, USA] diluted 1:1 with 0.5 mM EDTA Solution [Nacalai Tesque, Kyoto, Japan]/phosphate-buffered saline [PBS]) every 7 days. For cell attachment, culture dishes were precoated with iMatrix-511 silk (Nippi, Tokyo, Japan) at 0.5 µg/cm$^2$, and CultureSure Y-27632 (10 µM; FUJIFILM Wako Pure Chemical Corporation, Osaka, Japan) was added to the medium for 1 day to promote the cell survival and attachment.

### *Conventional method for induction of differentiation into LLCs*

The conventional method for inducing differentiation of hiPSCs into LLCs was

performed with some modifications to the previous literature (Ishida *et al.*, 2021). In brief, for EB formation, 3×$10^4$ 3AB4-NR5A1-hiPSCs were allowed to aggregate in 100 µL of maintenance medium (DMEM High Glucose [Nacalai Tesque] with 15% KnockOut SR [Life Technologies], CultureSure Y-27632 [10 µM], penicillin [50 units/mL], streptomycin [50 µg/mL], and doxycycline hyclate [1.5 µM; Tocris Bioscience, Bristol, UK]) and seeded into each well of non-adherent 96-well culture dishes (**PrimeSurface96M**; Sumitomo Bakelite, Tokyo, Japan) for incubation in a 5% $CO_2$ environment at 37°C for 6 days without replenishing the medium. Subsequently, four EBs were transferred to each well of 24-well culture dishes (**PrimeSurface24F**; Sumitomo Bakelite) containing 500 µL/well differentiation medium (DMEM High Glucose with 10% Fetal Bovine Serum [FBS] [Biowest, Nuaille, France], 8-Bromoadenosine-3',5'-cyclic Monophosphate Sodium Salt Hydrate [8Br-cAMP] [1 mM, Nacalai Tesque], forskolin [100 µM, Abcam, Cambridge, UK], penicillin [50 units/mL], streptomycin [50 µg/mL], and doxycycline hyclate [1.5 µM]). All media were refreshed every two to three days starting on day 6.

### *Sequential imaging of cellular morphological changes*

On day 6, micro-EBs were transferred to adherent culture dishes. A phase-contrast microscope (BioStudio; Nikon, Tokyo, Japan) was used to determine the time-lapse function. High-resolution images were acquired every 5 min for 48 h.

### *Measurement of testosterone concentrations in culture supernatants*

Culture supernatants were collected every three days, and testosterone concentrations were quantitatively measured using an electrochemiluminescence immunoassay

(ECLIA) performed by SRL (Tokyo, Japan).

*RT-PCR analyses*

Total RNA was extracted using TRIzol reagent (Life Technologies), and genomic DNA contamination was eliminated using a TURBO DNA-free kit (Life Technologies). A total of 650 ng of total RNA was reverse-transcribed into cDNA using the PrimeScript II 1st Strand Synthesis Kit (Takara, Shiga, Japan) in accordance with the manufacturer's instructions. Water served as the negative control, whereas total testicular RNA (BioChain, Newark, CA, USA) served as the positive control. The primer sequences used for RT-PCR are outlined below.

| **Gene name** | | **primer (5'-3' orientation)** |
|---|---|---|
| *GAPDH* | forward | accacagtccatgccatcac |
| | reverse | tccaccaccctgttgctgta |
| *OCT3/4* | forward | gacagggggagggggaggagctagg |
| | reverse | cttccctccaaccagttgccccaaac |
| *NANOG* | forward | tgaacctcagctacaaacag |
| | reverse | tggtggtaggaagagtaaag |
| *SOX2* | forward | gggaaatgggaggggtgcaaaagagg |
| | reverse | ttgcgtgagtgtggatgggattggtg |
| *NR5A1* | forward | aggagtacctgtaccacaagc |
| | reverse | tgcagcatttcgatgagcag |
| *STAR* | forward | ggttctcggctggaagagac |
| | reverse | gggacaggacctggttgatg |
| *CYP11A1* | forward | tgggtcgcctatcaccagta |
| | reverse | gttggccttgatgtcctcga |
| *CYP17A1* | forward | tggctctcttgctgcttacc |
| | reverse | gccacgaagacaggaaagga |
| *HSD3B1* | forward | cacatggcccgctccatac |
| | reverse | gtgccgccgtttttcagattc |
| *HSD17B3* | forward | gtcaacaatgtcggaatgcttc |
| | reverse | tgatgttacaatggatgaggctc |

*RNA sequencing*

At various stages of differentiation induction, total RNA was extracted from the cells using TRIzol (Life Technologies) and subsequently treated with the TURBO DNA-free kit (Life Technologies). The treated RNA was then sent to Macrogen (Seoul, South Korea; https://www.macrogen.com) for library preparation using the SMARTer stranded total RNA-seq kit (Takara Bio) according to the manufacturer's instructions. Paired-end RNA sequencing datasets were generated using an Illumina NovaSeq6000 (Illumina, San Diego, CA, USA). Using the Strand NGS software program (Strand Life Sciences, Bangalore, India), the reads were aligned to the human transcriptome reference sequence (hg38). Heat maps were created by converting transcripts per million (TPM) values to z-scores, and a PCA was performed on all differentiated cells using the R software program (Version 4.1.2 (2021-11-01), R Foundation for Statistical Computing, Vienna, Austria). Using the g:Profiler software program, we conducted pathway analyses.

### *Immunofluorescence assays*

Differentiated LLCs cultured in Nunc Cell-Culture-Treated Multidish (24-well plate; Life Technologies) were first fixed using 4% paraformaldehyde Phosphate Buffer Solution (FUJIFILM Wako Pure Chemical Corporation) for 15 min, followed by washing the cells with PBS three times. The cells were then treated with 0.5% Tween 20 (Nacalai Tesque) in 5% Blocking One (Nacalai Tesque) in PBS for 10 min at room temperature. Afterwards, the cells were incubated with primary antibodies overnight at 4°C. The primary antibodies used were anti-HSD17B3 (rabbit polyclonal antibody, dilution 1:100; Genetex, Irvine, CA, USA; #GTX114480), anti-LHCGR (rabbit polyclonal antibody, dilution 1:100; Genetex, #GTX100008), anti-StAR (rabbit polyclonal antibody, dilution 1:100; Genetex, #GTX105716), and anti-CYP17A1 (rabbit polyclonal antibody, dilution 1:50; Genetex,

#GTX56294). Normal rabbit IgG (4 µg/mL; FUJIFILM Wako Pure Chemical Corporation; #148-09551) was used as the isotype control. The following day, the cells were washed thrice with PBS. They were then incubated with secondary antibody (donkey anti-rabbit IgG, Alexa Fluor 594, dilution 1:500; Thermo Fisher Scientific) for 60 min at room temperature. Nuclear staining was performed using Hoechst 33342 (dilution, 1:10,000; Thermo Fisher Scientific). After the final PBS wash, cells were observed using an Olympus IX71 inverted microscope with an Olympus DP73 camera (Olympus Corporation, Tokyo, Japan). Quantitative measurements were performed using the ImageJ Fiji software program (National Institutes of Health, Bethesda, MD, USA) (Schindelin et al., 2012). In immunostaining of LHCGR (Figure S6C), cells without membrane permeabilization were not treated with 0.5% Tween 20.

### *Flow cytometry*

Flow cytometry was also used to evaluate the differentiation efficiency. Specifically, the cell samples were fixed with 4% paraformaldehyde phosphate buffer solution for 15 min at room temperature and subsequently permeabilized with 0.3% Triton X-100 (Nacalai Tesque)/PBS for 30 min at room temperature. The cells were then incubated with primary antibodies or isotype control for 30 min at 4°C, followed by labeling with secondary antibodies for an additional 30 min at 4°C. The primary antibodies and isotype control were the same as those used in the immunofluorescence assay described above, with only the following dilutions adjusted: anti-HSD17B3 (dilution 1:20), anti-LHCGR (dilution 1:20), anti-StAR (dilution 1:20), and anti-CYP17A1 (dilution 1:10). Normal rabbit IgG (20 µg/mL) was used as the isotype control. The secondary antibody used was donkey anti-rabbit IgG Alexa Fluor 488 (dilution 1:25; Thermo Fisher Scientific). The labeled

samples were analyzed using a FACSVerse instrument (BD Biosciences, San Jose, CA, USA).

### *One-step digital droplet PCR*

As previously reported (Kuroda et al., 2015), a one-step digital droplet PCR assay was performed to analyze the expression of *LIN28*, an undifferentiated marker. Total RNA was prepared as previously described. PCR mixtures (20 μL) were prepared using the One-Step RT-ddPCR Advanced Kit for Probes (Bio-Rad, Hercules, CA, USA). The mixture consisted of 5 μL of Supermix, 2 μL of Reverse Transcriptase, 1 μL of 300 mM DTT, 900 nM forward and reverse primers, 250 nM probe, and total RNA sample (100 ng). The primers and probe used for the analysis of the LIN28 gene in this study were designed based on the work of Kuroda et al. Specifically, the forward primer sequence for LIN28 is "CACGGTGCGGGCATCTG", and the reverse primer sequence is "CCTTCCATGTGCAGCTTACTC". Additionally, the probe sequence for LIN28 is "CGCATGGGGTTCGGCTTCCTGTCC". Droplets were generated using a QX200 Droplet Generator (Bio-Rad). RT-PCR was performed on a C1000 Touch Thermal Cycler (Bio-Rad). The thermal cycling conditions were as follows: reverse transcription at 50°C for 60 min, enzyme activation at 95°C for 10 min, and 40 cycles of a thermal profile comprising denaturation at 95°C for 30 s, and annealing/extension at 60°C for 1 min. After PCR amplification, products were denatured at 98°C for 10 min and cooled to 4°C. The fluorescence intensity of each droplet from the samples was measured using a QX200 Droplet Reader (Bio-Rad). Positive droplets containing amplification products were distinguished from negative droplets and counted by applying a fluorescence amplitude threshold in the QuantaSoft software program (Bio-Rad). The threshold was manually

determined at the highest point in the fibroblast sample droplet group. To calculate the positivity rate, the number of positive droplets was divided by the total number of droplets.

*Generation of hiPSC lines with the forced expression of NR5A1 using the Tet-Off system*

*NR5A1* cDNA was introduced into the PB-TA-MCS (KW107) transposon vector using Gateway Cloning (Thermo Fisher Scientific) to create PB-TA-NR5A1. PB-CAG-tTA-IREShyg (KW1526) expresses Tet transactivator (tTA) and a hygromycin resistance gene and was constructed using the MultiSite Gateway Cloning technique with LR Clonase$^{TM}$ II Plus (Thermo Fisher Scientific). These two transposon vectors were then co-delivered into hiPSCs (121-3) along with pCAG-PBase (KW158), which expresses the piggyBac transposase, using FuGENE HD (Roche, Basel, Switzerland), following the manufacturer's instructions. hiPSCs bearing transposons were enriched after 50 µg/ml of Hygromycin B (FUJIFILM Wako Pure Chemical Corporation) was added to the medium for 24 h. hiPSCs with the forced expression of *NR5A1* using the Tet-Off system were generated as described and maintained under the same culture conditions as the original hiPSCs, with the addition of 1 µM doxycycline to the maintenance medium.

*Transmission Electron Microscope (TEM)*

The cell samples for TEM were fixed in phosphate-buffered 2% glutaraldehyde (Electron Microscopy Science) and subsequently post-fixed in 2% osmium tetroxide (Heraeus Chemicals, South Africa) for 2 h in an ice bath. The specimens were then dehydrated in a graded ethanol series and embedded in an epoxy resin (TAAB Laboratories). Ultrathin sections were obtained using an ultramicrotome. Ultrathin sections stained with uranyl

acetate for 15 min and lead staining solution for 2 min were subjected to TEM observation at 100 kV (JEM-1400Flash, JEOL).

*scRNA-seq analyses*

We performed scRNA-seq using the BD Rhapsody Express Single-Cell Analysis System (BD Biosciences) and analyzed the data using the Seurat software program (http://satijalab.org/seurat/, R package version 4.1.2).

First, the LLCs were dissociated by adding 0.5X TrypLE Select to the culture dishes and incubating at 37°C for 2 min. Following the workflow manual, the cells were suspended in 1X PBS and loaded into cartridges along with the beads. Reverse transcription and PCR were performed. cDNA amplification, sequencing library preparation and a sequencing analysis using the TAS-Seq approach was performed by ImmunoGeneTeqs, Inc (Chiba, Japan). Next, we loaded scRNA-seq data from the publicly available dataset of testes from 3 adult males (17, 24, and 25 years old; GSE112013) (Guo et al., 2018) and our generated LLCs dataset. The cells were filtered to retain only those with high-quality expression profiles, setting min_cells = 3 and min_features = 500. For the adult testis dataset, we retained cells with 500 < nFeature_RNA < 5000 and percentage _mt <10. For the LLC dataset, we retained cells with 5000 < nFeature_RNA < 10000 and percent_mt <20. Next, we normalized the data using the LogNormalize method and identified the top 2,000 variable features for each dataset using the variance-stabilizing transformation (VST) method. We then performed unsupervised clustering using the top 10 principal components (PCs) and a resolution of 0.15 for both datasets. For data integration, we merged the two datasets into one Seurat object and prepared it for further analyses. The resulting cell clusters were visualized

using UMAP. In the UMAP plot, we identified distinct cell populations using specific marker genes: DLK1 for Leydig cells, MYH11 for myoid cells, CD163 for macrophages, SOX9 for Sertoli cells, DDX4 for germ cells, and VWF for vascular endothelial cells. This allowed us to compare the generated LLCs with normal testis cells and to understand their transcriptional landscape. Their expression patterns were visualized using feature plots, violin plots, dot plots, and heat maps. The top 50 genes characteristic of LC, LLC subset 1, and LLC subset 2 are also listed. From each set, five genes that were relevant to Leydig cells were identified through a PubMed search, now listed on the right side of the heatmap (Figure 3E). Cells at days 0, 6, and 28 of differentiation induction were also subjected to an scRNA-seq analysis in the same manner, and alterations in gene expression with the progression of differentiation induction days were represented in UMAP plots.

*Passaging, cryopreservation, and thawing of LLCs*

When LLCs reached confluence in 6-well plates, 0.5X TrypLE Select was added, and cells were dissociated by incubation at 37°C for 2 min. The cells were harvested and resuspended in Step 2 medium without cAMP. One well of cells was distributed into six wells for passaging, and once confluence was reached, passaging was repeated in the same manner. Cells were stained with Trypan Blue Stain (Life Technologies) and counted using a Countess3 Automated Cell Counter (Invitrogen). To determine whether or not LLCs could be cryopreserved and thawed, LLCs on day 17 of differentiation induction were dissociated with 0.5X TrypLE Select as described above and suspended in STEM-CELLBANKER (ZENOAQ, Fukushima, Japan) at a concentration of $1.6 \times 10^6$ cells/500 µL. Cell suspensions were dispensed in 500 µL aliquots into cryopreservation vials. After

freezing the vials at -80°C for 24 h, the frozen vials were transferred to a liquid nitrogen storage tank. When thawing cells, vials were warmed in a 37°C water bath, and LLCs were resuspended in cAMP-free Step 2 medium and cultured in 1 well of a 6-well plate, with the medium changed every 2 days.

*Response of LLCs to gonadotropic hormones*

We investigated whether LLCs were capable of secreting testosterone in response to gonadotropic hormonal stimuli. To achieve this, we first measured the baseline testosterone concentration secreted by LLCs over a 24-hour period (-24 to 0 h). At 0 h, 5 ng/mL LH (Sigma Aldrich, St. Louis, MO, USA) or 1 U/mL hCG (gonadotropin; ASKA Pharmaceutical Co., Ltd., Tokyo, Japan) was added to the culture medium. The testosterone concentration in the culture supernatant was measured 3 h after addition.

*Transfection of Venus-Akaluc*

The target gene from pcDNA3 Venus-Akaluc (RIKEN, Japan) was integrated into the pMXs retrovirus vector using an infusion reaction, resulting in the generation of pMXs-Venus-Akaluc. The pcDNA3 Venus-Akaluc was provided by the RIKEN BRC through the National BioResource Project of the MEXT, Japan (cat. RDB15781). The pMXs-Venus-Akaluc plasmid vector was transfected into packaging cells (PLAT-A) using FuGENE HD (Roche) according to the manufacturer's instructions. The culture medium was changed on the day following transfection, and on the second day, the culture supernatant was collected and passed through a 0.45-μm filter. The filtered supernatant was supplemented with 8 μg/mL polybrene. LLCs used for infection were those on the second day (P1Day2) of culture following a single passaging event. Following

transfection, Venus-Akaluc-LLCs were cultured in the Step 2 medium without 8Br-cAMP. On day 21 post-retroviral infection, the expression of green Venus fluorescent proteins was visualized using a fluorescence microscope. The substrate AkaLumine-HCl (FUJIFILM Wako Pure Chemical Corporation) was added to 24-well plates at a concentration of 125 μg/well, and the luminescence of AkaBLI was observed using an IVIS Lumina LT imaging system (PerkinElmer, Waltham, MA, USA). The following parameters were used to capture images: exposure time of 60 s, medium binning of 8, XFOV field of view measuring 24 × 24 cm, and f/stop value of 1. The bioluminescence images produced by the IVIS system were analyzed using the specialized Living Image software program (PerkinElmer).

### *Adherent culture of LLCs on PET membranes*

On day 21 of differentiation induction, LLCs cultured in two wells of the six-well plates were incubated with 0.5X TrypLE Select for 2 min to dissociate from the culture dish. The LLCs were resuspended in 2 mL of Step 2 medium and loaded into one well of a 0.4-μm pore size PET-Track-attached-membrane-12-well format (Corning, Corning, NY, USA; catalog #353180). Subsequently, this membrane was placed in 1 well of a 12-well TC-treated Polystyrene Permeable support anion plate (Corning; catalog #353503) containing 2 mL of Step 2 medium per well and incubated at 37°C, 5% $CO_2$.

### *Procurement and management of immunocompromised mice*

All experimental protocols involving animals were approved by the Animal Experimentation Committee of Kobe University. The approval number for this study is P230206.

Four-week-old female NSG mice (NOD.Cg-Prkdcscid Il2rgtm1Wjl/SzJ; Jackson Laboratory, Kanagawa, Japan) were obtained and maintained in a sterile environment with free access to food and water. A minimum of five days was allowed for acclimation. Mice were divided into four groups: Group 1 received PET membranes with Venus-Akaluc-transfected LLCs, Group 2 received PET membranes with LLCs without Venus-Akaluc, Group 3 received only PET membranes without cells, and Group 4 received no cells or membranes (Figure 5D). On the day of transplantation, the mice were anesthetized with isoflurane, and a small incision was made in the back for cell transplantation. PET membranes were transplanted subcutaneously with the cell-attached side facing the body surface, and the skin was sutured. After the surgery, the mice were monitored for signs of discomfort or distress.

*Measurement of AkaBLI luminescence and serum testosterone levels in mice*

Three days after transplantation, we anesthetized the mice again with isoflurane and injected AkaLumine-HCl (750 μg per body) dissolved in 300 μl of saline into the abdominal cavity. After 10 min in the abdominal cavity, we measured the AkaBLI luminescence levels using an IVIS Lumina LT imaging system. The settings were an exposure time of 60 s, medium binning of 8, XFOV field of view measuring 12.9×12.9 cm, and an f/stop value of 1. Mice were euthanized by cervical dislocation, and blood was immediately drawn from their hearts. Subsequently, serum hormone levels were measured at the Integrated Center for Mass Spectrometry at Kobe University (https://www.med.kobe-u.ac.jp/icms/icms/index.html). The skin of the mice was cut open and observed at the engraftment site. We removed the PET membranes and

immunostained them using an anti-LHCGR antibody as described in the immunofluorescence assay section above.

*Transplantation of LLC clusters into the mice*

Tet-Off LLCs on days 43-67 of differentiation induction, cultured in 6-well plates, were treated with 400 µL/well of 0.5X TrypLE Select and incubated for 2 minutes at 37°C, 5% $CO_2$ incubator. The cells were then rinsed with 1X PBS and detached using a cell scraper (Sumitomo Bakelite) in a scrubbing manner to form cell clusters. These clusters were then subcutaneously transplanted into anesthetized 8- to 12-week-old immunocompromised female mice, as described above. Three days post-implantation, the mice were euthanized by cervical dislocation, and heart blood samples were collected for the measurement of various hormone levels, as previously described. In experiments involving male mice, five-week-old immunocompromised mice were anesthetized using isoflurane, and the bilateral testes were surgically removed. Tet-Off LLCs were subsequently transplanted subcutaneously in the form of cell clusters as described previously. Four days later, blood samples were collected from the heart, and serum concentrations of various hormones were quantified.

*Figures*

Several figures were created using the BioRender software program (BioRender.com). These illustrations were used to depict specific experimental processes and results, adhering to the platform's guidelines for academic and scientific use.

*Declaration of generative AI and AI-assisted technologies in the writing process*

During the preparation of this work the authors used DeepL (https://www.deepl.com/translator) and ChatGPT-4 (https://chat.openai.com/) in order to translate specific sentences from Japanese to English. After using these tools, the authors reviewed and edited the content as needed and take full responsibility for the content of the publication.

## SUPPLEMENTAL REFERENCES


Guo, J., Grow, E.J., Mlcochova, H., Maher, G.J., Lindskog, C., Nie, X., Guo, Y., Takei, Y., Yun, J., Cai, L., et al. (2018). The adult human testis transcriptional cell atlas. Cell Res *28*, 1141-1157. 10.1038/s41422-018-0099-2.

Ishida, T., Koyanagi-Aoi, M., Yamamiya, D., Onishi, A., Sato, K., Uehara, K., Fujisawa, M., and Aoi, T. (2021). Differentiation of Human Induced Pluripotent Stem Cells Into Testosterone-Producing Leydig-like Cells. Endocrinology *162*. 10.1210/endocr/bqab202.

Kuroda, T., Yasuda, S., Matsuyama, S., Tano, K., Kusakawa, S., Sawa, Y., Kawamata, S., and Sato, Y. (2015). Highly sensitive droplet digital PCR method for detection of residual undifferentiated cells in cardiomyocytes derived from human pluripotent stem cells. Regen Ther *2*, 17-23. 10.1016/j.reth.2015.08.001.

Murai, N., Koyanagi-Aoi, M., Terashi, H., and Aoi, T. (2023). Re-generation of cytotoxic γδT cells with distinctive signatures from human γδT-derived iPSCs. Stem Cell Reports *18*, 853-868. 10.1016/j.stemcr.2023.02.010.

Nakagawa, M., Taniguchi, Y., Senda, S., Takizawa, N., Ichisaka, T., Asano, K., Morizane, A., Doi, D., Takahashi, J., Nishizawa, M., et al. (2014). A novel efficient feeder-free culture system for the derivation of human induced pluripotent stem cells. Sci Rep *4*, 3594. 10.1038/srep03594.

Schindelin, J., Arganda-Carreras, I., Frise, E., Kaynig, V., Longair, M., Pietzsch, T., Preibisch, S., Rueden, C., Saalfeld, S., Schmid, B., et al. (2012). Fiji: an open-source platform for biological-image analysis. Nat Methods *9*, 676-682. 10.1038/nmeth.2019.
